# CLK1/CLK2-driven signalling at the *Leishmania* kinetochore is captured by spatially referenced proximity phosphoproteomics

**DOI:** 10.1038/s42003-022-04280-1

**Published:** 2022-11-28

**Authors:** Vincent Geoghegan, Juliana B. T. Carnielli, Nathaniel G. Jones, Manuel Saldivia, Sergios Antoniou, Charlotte Hughes, Rachel Neish, Adam Dowle, Jeremy C. Mottram

**Affiliations:** 1 York Biomedical Research Institute and Department of Biology, University of York, Wentworth Way, Heslington, York, YO10 5DD UK; 2 Novartis Institute for Tropical Diseases, Emeryville, CA USA; 3 Bioscience Technology Facility, Department of Biology, University of York, York, YO10 5DD UK

**Keywords:** Kinases, Parasite biology

## Abstract

Kinetochores in the parasite *Leishmania* and related kinetoplastids appear to be unique amongst eukaryotes and contain protein kinases as core components. Using the kinetochore kinases KKT2, KKT3 and CLK2 as baits, we developed a BirA* proximity biotinylation methodology optimised for sensitivity, XL-BioID, to investigate the composition and function of the *Leishmania* kinetochore. We could detect many of the predicted components and also discovered two novel kinetochore proteins, KKT24 and KKT26. Using KKT3 tagged with a fast-acting promiscuous biotin ligase variant, we took proximity biotinylation snapshots of the kinetochore in synchronised parasites. To quantify proximal phosphosites at the kinetochore as the parasite progressed through the cell cycle, we further developed a spatially referenced proximity phosphoproteomics approach. This revealed a group of phosphosites at the kinetochore that were highly dynamic during kinetochore assembly. We show that the kinase inhibitor AB1 targets CLK1/CLK2 (KKT10/KKT19) in *Leishmania* leading to defective cytokinesis. Using AB1 to uncover CLK1/CLK2 driven signalling pathways important for kinetochore function at G2/M, we found a set of 16 inhibitor responsive kinetochore-proximal phosphosites. Our results exploit new proximity labelling approaches to provide a direct analysis of the *Leishmania* kinetochore, which is emerging as a promising drug target.

## Abstract (web summary)

Combination of proximity biotinylation and protein cross-linking allows proximity phosphoproteomics of the kinetochore in *Leishmania* parasites during the cell cycle and captures perturbations at the kinetochore after treatment with a protein kinase inhibitor.

## Introduction

The kinetochore is a large protein complex present in all eukaryotes that links chromosomes to force-generating spindle microtubules, ensuring accurate chromosome segregation during cell division^1^. The inner kinetochore is bound to a specialised region of chromatin, the centromere, throughout the cell cycle, whilst the outer kinetochore assembles during mitosis. Intriguingly, for a complex mediating a fundamental cellular process, the kinetochore is not universally well conserved across the eukaryote domain of life. A recently discovered example of this plasticity are the inner kinetochore proteins in the related kinetoplastid parasites *Trypanosoma* and *Leishmania* which do not bear any readily detectable sequence homology to kinetochore components in other eukaryotes^2,3^. Another divergent feature is that four protein kinases, KKT2, KKT3, KKT10 (CLK1) and KKT19 (CLK2), are present in the structure of the kinetochore. Studying the molecular detail of the *Leishmania* kinetochore may therefore reveal unique solutions that have evolved to solve the fundamental problem of chromosome segregation and provide information to drug discovery programmes targeting this essential protein complex.

Based on homology with trypanosome kinetochores, *Leishmania* kinetochores are predicted to contain two sets of paralogous kinases: KKT2, KKT3; CLK1 and CLK2^2^. Our previous large-scale study of *Leishmania* protein kinases indicated that KKT2 and KKT3 are each essential for promastigote parasite survival, whilst only one of CLK1 or CLK2 is required for parasite viability^4^. Interestingly, KKT2/KKT3 do not belong to any known eukaryotic protein kinase family based on the primary sequence of the kinase domain and appear to be core members of the inner kinetochore, localising to chromatin throughout the cell cycle via recently characterised zinc finger binding domains^5^. CLK1/CLK2 belong to the CMGC kinase family and are similar in sequence to cdc2-like kinases (CLKs), which phosphorylate and regulate splicing factors in vertebrates. In trypanosomes, CLK1/CLK2 localise to kinetochores and regulate the transition from metaphase to anaphase in late mitosis^6^. CLK1/CLK2 are targets of the recently identified covalent amidobenzimadazole inhibitor AB1, which causes disruption of trypanosome kinetochore function during mitosis, leading to chromosome segregation errors, aneuploidy and parasite death^7^. AB1 is a promising anti-parasite drug candidate to target kinetoplastids, however, its mode of action in *Leishmania* has not been explored.

To gain insight into the kinetochore and kinetochore protein kinases in *Leishmania*, we developed a high-sensitivity proximity biotinylation workflow by combining proximity biotinylation with protein cross-linking (XL-BioID) to investigate the molecular environment around KKT2, KKT3 and CLK2. We endogenously tagged KKT2, KKT3 and CLK2 with the promiscuous biotin ligase BirA* and found that XL-BioID leads to a ~10-fold increase in signal intensity for purified, proximal kinetochore proteins compared to conventional BioID. We detected several kinetochore subunits in proximity to all three kinases as well as members of the chromosome passenger complex, microtubule-binding proteins, subunits of the origin of replication complex and factors regulating chromatin architecture. A fluorescence co-localisation microscopy screen of proximal proteins revealed novel components of the kinetochore, KKT24 and KKT26. In other eukaryotes, the kinetochore is a dynamic structure, assembling on centromeres during the cell cycle so that it is fully formed for the capture of mitotic spindle microtubules when the cell divides. To follow this process in *Leishmania*, we developed a spatially referenced proximity phosphoproteomics workflow. Parasites expressing endogenously miniTurbo-tagged KKT3 or miniTurbo-tagged BDF5 spatial reference were synchronised and XL-BioID was used to take a spatial ‘snapshot’ at S and G2/M phases. We could identify and quantify both proximal proteins and phosphosites from the same sample, enabling us to follow the dynamics of proximal proteins and proximal phosphosites at the kinetochore through the cell cycle, revealing proximal phosphosites with dynamic profiles distinct from that of the parent protein at the complex. Furthermore, we have demonstrated that AB1 specifically targets CLK1/CLK2 in *Leishmania*, allowing us to study the biological role of these redundant protein kinases on cell cycle progression. This revealed that the cell arrest in G2/M caused by CLK1/CLK2 inhibition is mainly due to the blockage of cytokinesis in *Leishmania*. To understand defects at the leishmania kinetochore caused by inhibition of CLK1/CLK2, we used spatially referenced proximity phosphoproteomics to detect a set of kinetochore proximal phosphosites that were significantly reduced in G2/M stage parasites after treatment with AB1. Quantitative information on proximal levels of proteins allowed us to distinguish between proximal phosphosites that reduced due to complex dissociation and those that reduced whilst the corresponding protein remained proximal. Amongst the latter, were 3 phosphosites on KKT2, which is a known substrate for CLK1/CLK2 in *T. brucei*. Finally, to understand the importance of KKT24 and KKT26 for *Leishmania* cell division, we used Cas9 genome editing to insert loxP sites flanking the endogenous genes. Inducible deletion of KKT24 or KKT26 was lethal for parasites, lack of KKT24 led to cells accumulating in mitosis, suggesting a defect at this stage, by contrast deleting KKT26 did not cause cells to accumulate at a particular cell cycle stage.

## Results

### XL-BioID increases in vivo capture of proximal proteins with DSP cross-linking

**Fig. 1:**
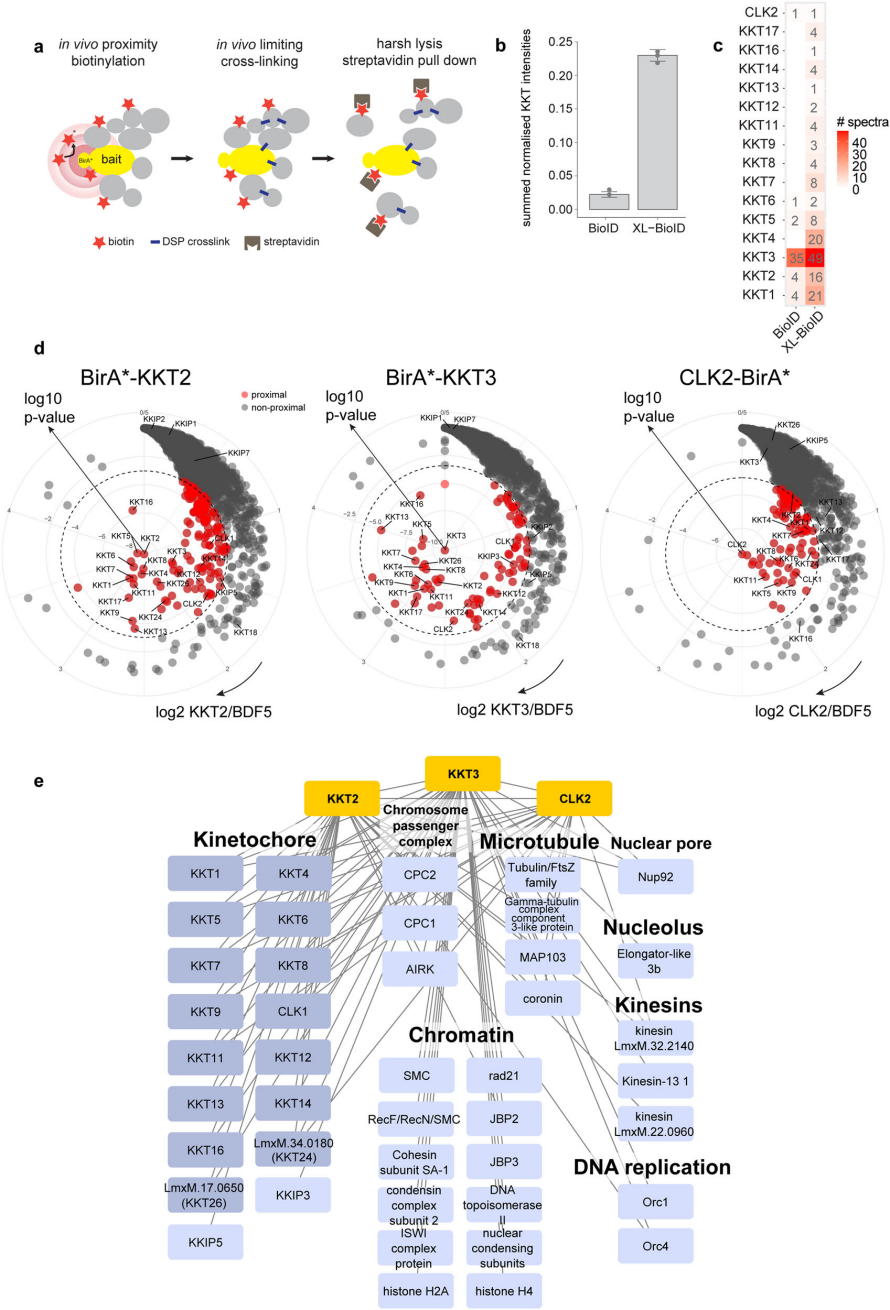
Mapping the proximal environment of kinetochore kinases KKT2, KKT3, CLK2 with XL-BioID. **a** Schematic of XL-BioID. The addition of exogenous biotin initiates in vivo biotinylation of proteins proximal to the BirA* tagged bait protein. A membrane permeable cross-linker, DSP, is then used in a second in vivo proximity capturing step to covalently link any proximal proteins that escaped biotinylation. Biotin tagging and covalent linking survive harsh lysis enabling the complex to be solubilised for affinity purification. **b** Comparison of kinetochore protein amounts purified in conventional BioID and XL-BioID. Label-free intensities of kinetochore proteins (KKTs) were summed and normalised to the amount of purified bait (BirA*::KKT3). Median and standard deviation are shown for three independent replicates. **c** Fragmentation spectra matching KKTs in BioID and XL-BioID. A median number of spectra matching to KKTs from three independent replicates. **d** KKT2, KKT3, CLK2-proximal proteins identified with XL-BioID. Enrichment against a BirA*-BDF5 spatial reference is plotted. Red points are proximal proteins, grey points are non-proximal. The dashed line indicates a 1% false discovery rate (FDR) threshold. **e** Selected KKT2, KKT3 and CLK2-proximal proteins. Ninety-eight proteins were significantly enriched and are classed as proximal at 1% (FDR). Only those proteins with annotated functions are shown. Edges indicate bait-prey associations.

Proximity-dependent biotin identification (BioID) is a powerful method for analysing a complex of interest, however, it does not always give good coverage of a complex and has a labelling radius of ~10 nm^8^. The *Leishmania* kinetochore is expected to be at least 70 nm in length. We, therefore, sought to improve the coverage of proximity biotinylation by adding a second in vivo proximity capturing step. We used dithiobis(succinimidyl propionate) (DSP), a membrane permeable amine-reactive cross-linker with a 12 Å spacer to covalently link proximal proteins that escaped biotinylation; we, therefore, call this approach XL-BioID (Fig. 1a). The covalent links withstand the harsh solubilisation and washing that are a key benefit of the proximity biotinylation workflow. Because capturing proximity information is not solely reliant on protein-crosslinking, lower concentrations of cross-linker can be used to minimise co-purification of non-proximal proteins.

To demonstrate the benefits of XL-BioID, we N-terminally endogenously tagged the *Leishmania* kinetochore protein kinase KKT3 with the promiscuous biotin ligase BirA* using Cas9-mediated genome engineering^9^. We then performed proximity biotinylation with and without the second cross-linking step, assessing the number and label-free intensity of kinetochore proteins identified by mass spectrometry. The sum of kinetochore protein label-free intensities were normalised to the bait intensity, resulting in a normalised label-free intensity of 0.23, which was ~10-fold higher than in conventional BioID (0.022, Fig. 1b). The increased amount of proximal material allowed the identification of 16 out of the 21 expected inner kinetochore proteins in XL-BioID against 6 from conventional BioID without a cross-linker (Fig. 1c and Supplementary Data 1).

### Proximal environment of kinetochore kinases captured by XL-BioID

A distinct feature of kinetoplastid kinetochores is the presence of protein kinases that are apparently core components^2^. To understand the proximal environment of these kinases, we used N-terminally BirA* tagged KKT2, KKT3 and C-terminally tagged CLK2 and XL-BioID to identify proximal proteins (Supplementary Figs. 1, 2). Identified proteins were quantified by precursor intensity-based label-free quantification to calculate enrichment against a control sample derived from parasites expressing a BirA* tagged bromodomain-containing protein BDF5, which also localises to the nucleus, but is primarily found on chromatin at sites of transcription initiation/termination and thus serves as a spatial reference^10^. From the three baits, a total of 229 high-confidence proximal proteins were identified at 1% FDR (Fig. 1d and Supplementary Data 2,3). KKT2 and KKT3 were associated with 149 and 79 proximal proteins, respectively, CLK2 was proximal to 88 proteins. Proximity maps of enriched proteins are similar for KKT2 and KKT3, but the proximity map for CLK2 shows a weaker enrichment for kinetochore subunits (Fig. 1d), KKT2 and KKT3 co-enrich 16 other kinetochore proteins, CLK2 co-enriched with 11 kinetochore subunits, likely due to its more transient association to the kinetochore^2,6^. In total, 20 inner or outer kinetochore proteins were identified, including two novel components, LmxM.34.0180 (KKT24) and LmxM.17.0650 (KKT26). Gene ontology enrichment analysis of proximal proteins identified ‘kinetochore’ and ‘chromosome, centromeric region’ as the most significantly enriched terms, indicating the BirA* tagged protein kinases were sampling the intended set of proximal proteins (Supplementary Fig. 3). Chromosome segregation is driven by the mitotic spindle and spindle-associated proteins such as kinesins, over-representation of the GO terms ‘spindle’ and ‘spindle pole’ indicate these structures became labelled as chromosomes segregated during mitosis. An important group of spindle-associated proteins, the chromosome passenger complex, was identified, including the *Leishmania* orthologue of human Aurora B kinase, AIRK, which is required for the assembly of the mitotic spindle in trypanosomes (Fig. 1e)^11,12^.

KKT2 and KKT3 are constitutively bound to chromatin at the centromere, which is the assembly site for the kinetochore^2,5^. We detected several proximal proteins with activities in the regulation of chromatin structure, such as a rad21, cohesin subunit, condensin subunit 2, SMC and nuclear condensing subunits (Fig. 1e). A key molecule that epigenetically specifies a region of chromatin as the centromere in other organisms is the centromeric histone CENP-A^13^. *Leishmania* and other kinetoplastids are notable in lacking this histone variant, raising the question of how the centromere is defined in these eukaryotes. We found JBP2 proximal to KKT2 and KKT3, JBP3 was also found proximal to KKT3. JBP2 and JBP3 are involved in the synthesis and binding of base-J, respectively, a glycosylated thymidine DNA base found only in kinetoplastids^14^. Base-J has been shown to be enriched at centromeres^15^, the finding that JBP2 and JBP3 are close to the kinetochore raise the possibility of base-J having a role in specifying the kinetochore binding site in *Leishmania*. The centromere in *Leishmania* is also the major origin of replication for chromosomes, explaining the enrichment of the origin of replication proteins Orc1 and Orc4^16^. After replication of DNA, the cohesion of sister chromatids is established, requiring the cohesin complex, which has been shown to accumulate at centromeres in budding yeast^17^. Sister chromatids may also be held together by DNA catenation, which also appears to be concentrated at centromeres, until the resolving action of DNA topoisomerase II at metaphase^18^. We found both cohesin and DNA topoisomerase II to be proximal to the kinetochore, evidence that similar mechanisms underlie chromatid cohesion in *Leishmania*. 64 of the 229 proximal proteins have no annotated function and may represent novel factors involved in chromosome segregation (Supplementary Data 2).

### XL-BioID enables a spatially referenced proximity phosphoproteomics view of kinetochore assembly

**Fig. 2:**
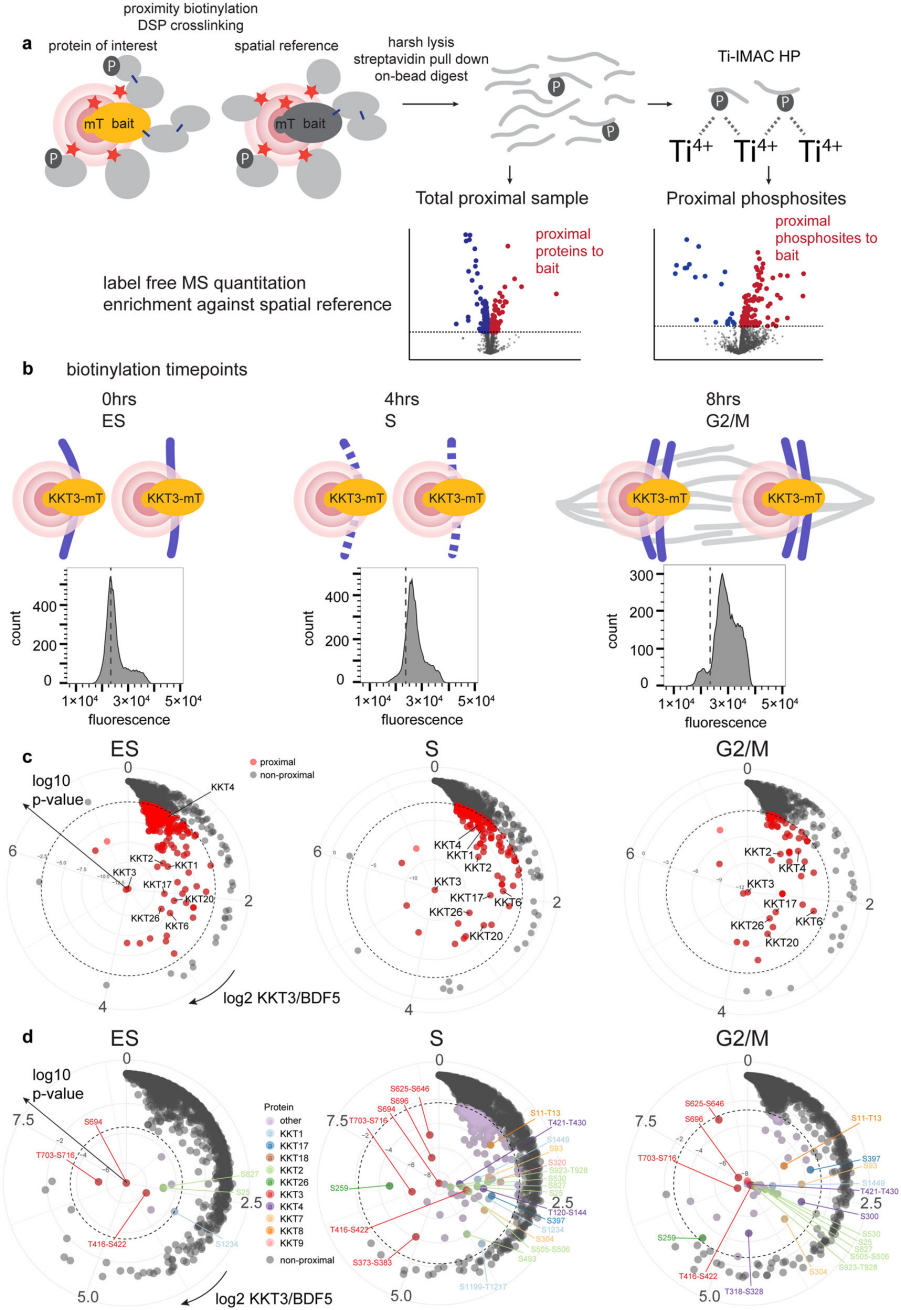
Dynamics of kinetochore proximal phosphosites during kinetochore assembly revealed by spatially referenced proximity phosphoproteomics. **a** Schematic of spatially referenced proximity phosphoproteomics workflow to quantify proximal proteins and proximal phosphosites from the same sample. Proximity biotinylation and DSP protein-crosslinking capture proximal proteins in the protein of interest sample and spatial reference sample. Proteins are on-bead digested into peptides for protein and phosphopeptide identification. Proteins or phosphosites significantly enriched (red dots) compared to the spatial reference sample are classed as proximal. **b** Using KKT3::mT and spatially referenced proximity phosphoproteomics to follow kinetochore assembly during the cell cycle in synchronised parasites. A 30 min biotinylation ‘snapshot’ was taken at three timepoints after release from hydroxyurea synchronisation. BDF5::mT was used as a compartment-matched spatial reference. Histograms of parasite DNA content are shown at each timepoint. The dotted line represents early S (ES) phase **c** Radial plots of proteins enriched in KKT3::mT samples compared to spatial reference BDF5::mT samples at each timepoint. Log2 fold enrichment increases in a clockwise direction, log10 *p* values increase from the centre outwards. The dashed line indicates a 1% FDR cutoff for distinguishing proximal (red points) and non-proximal (grey points) proteins. Label-free quantification was performed on five independent biological replicates. **d** Radial plots of phosphosites enriched in KKT3::mT samples compared to spatial reference BDF5::mT samples at each timepoint. The dashed line indicates a 5% FDR cutoff for distinguishing proximal (coloured points) and non-proximal (grey points) phosphosites. Label-free quantification was performed on five independent biological replicates. Where a phosphosite could not be confidently localised, the possible phosphorylated region is indicated.

Our discovery that XL-BioID purifies ~10-fold more proximal material than the standard BioID workflow suggested to us that it may be possible to enrich phosphosites from proximal proteins isolated by XL-BioID. We designed a workflow which uses XL-BioID to enrich proximal proteins that are digested on streptavidin beads to release the peptides. 60% of this is then used to enrich proximal phosphopeptides using a Ti-IMAC resin designed for highly sensitive phosphopeptide enrichment. The remaining 40% of peptides are used as the ‘Total’ proximal sample, thus from the same sample, both proximal proteins and their phosphosites can be quantified (Fig. 2a). A key component of our spatially referenced proximity phosphoproteomics approach was the use of BDF5 as a spatial reference^10^. BDF5 localises to chromatin, but to different regions than the kinetochore^19^. It, therefore, provides a compartment-matched set of background ‘bystander’ proteins and phosphosites that are captured by the workflow, but are not proximal to the kinetochore (Fig. 2a). As there is minimal knowledge on how the *Leishmania* kinetochore assembles or is regulated by phosphorylation during cell cycle progression, we used spatially referenced proximity phosphoproteomics to follow KKT3 proximal proteins and phosphosites in synchronised parasites as they progressed through the cell cycle. To achieve this, we employed the engineered BirA* variant, miniTurbo, which enables proximity biotinylation at a time resolution of 10 min compared to 18–24 h for BirA*^20^. We endogenously tagged both alleles of the spatial reference BDF5 and KKT3 with miniTurbo, since KKT3 is an essential protein kinase in *Leishmania* this indicated that the tagged protein was functional (Supplementary Figs. 4–7)^4^. Parasites were synchronised with hydroxyurea at early S (ES) phase, then released to resume the cell cycle^15^. A 30 min biotinylation was performed at 0, 4 and 8 h following hydroxyurea release, which samples KKT3 proximal proteins and phosphosites at ES, S and G2/M stages, respectively. At each timepoint, parasites were analysed by flow cytometry, confirming synchronous progression through the cell cycle (Fig. 2b). KKT3 proximal proteins and phosphosites were accurately detected by using label-free quantitation to calculate enrichment relative to samples from parasites expressing miniTurbo-tagged BDF5 (Supplementary Data 4, 5).

The bait itself, KKT3, was the most significantly enriched protein at all timepoints and is, therefore, the central point in proximity maps of quantified proteins (Fig. 2c). Because the extent of enrichment of a protein is not only dependent on proximity, but also copy number at the complex and the number of lysine residues available for biotinylation, relative distances of proteins to the bait cannot be determined from proximity labelling data. However, it is possible to infer changes at the kinetochore complex by comparing enrichment between timepoints of the cell cycle. We found that a set of kinetochore proteins consisting of KKT1, KKT2, KKT4, KKT6, KKT17, KKT20 and KKT26 were proximal to KKT3 early in the cell cycle at ES (Fig. 2c left plot). As the parasites progressed through the cell cycle, the enrichment of KKT2, KKT6, KKT17 and KKT26 remained relatively constant, indicating that these proteins may form part of the stable chromatin-bound inner kinetochore (Fig. 2c middle and right plots). KKT2 and KKT17 have indeed been shown to be constitutively present at the kinetochore in *T. brucei*. Our proximity data showed that KKT4 and KKT20 became 1.4-fold and 2.4-fold more enriched, respectively, through the cell cycle. KKT4 is the only kinetoplastid kinetochore protein demonstrated to bind microtubules and, therefore, likely plays an important role in attaching the kinetochore to the mitotic spindle^21^. This process takes place later in the cell cycle, which is consistent with our proximal data showing KKT4 reaching peak enrichment at G2/M (Fig. 2c right plot). KKT20 showed the largest increase in proximal enrichment through the cell cycle, which is consistent with fluorescence localisation data for this protein from *T. brucei*^22^. In contrast, KKT1 was proximal at ES and S phase, but not at G2/M phase. Microscopy data suggest KKT1 remains localised to the kinetochore throughout the cell cycle, a discrepancy that could be due to such studies being limited in resolution to ~300 nm, whilst proximity biotinylation can report on localised re-arrangements at a much higher spatial resolution. Overall, the set of KKT3 proximal proteins decreases in complexity through the cell cycle and may reflect the fact that the kinetochores are proximal to the origins of replication which have the highest activity at the early S phase.

Proximal phosphosites to KKT3 show a different trend during the cell cycle, being a limited set at ES and peaking in diversity at the S phase. The most significantly enriched phosphosite at all timepoints was S694 on the bait itself (Fig. 2d). At ES, nine proximal phosphosites on six proteins were detected. The only detected kinetochore phosphosites proximal to KKT3 were KKT1 (S1234) and KKT2 (S25, S827), suggesting the kinetochore was in a hypophosphorylated state (Fig. 2d, left plot). By S phase, the phosphorylation landscape surrounding KKT3 had dramatically changed. About 153 proximal phosphosites on 118 proteins could be detected. Proximal phosphosites could now be identified on KKT1-KKT4, KKT7-9, KKT17-18 and KKT26, as well as a set of non-kinetochore proteins. At G2/M, 43 proximal phosphosites were detected on 28 proteins. Most of the kinetochore phosphorylation sites remained proximal at G2/M, whilst the number of non-kinetochore proximal phosphosites had reduced.

### Dynamics of kinetochore phosphorylation during the cell cycle

**Fig. 3:**
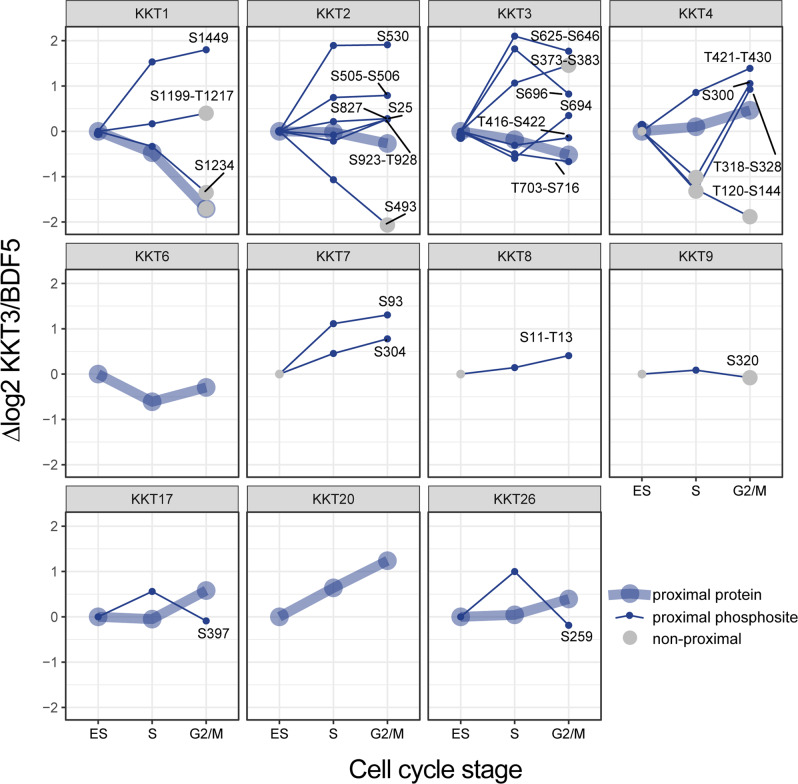
Proximal profile plots of kinetochore proteins and phosphosites during the cell cycle. Kinetochore proteins and phosphosites proximal in at least one of the timepoints are shown. At each timepoint, mean log2 fold enrichments against BDF5::mT spatial reference are normalised to the log2 fold enrichment at time 0, *n* = 5. Where a phosphosite could not be confidently localised, the possible phosphorylated region is indicated.

Since both protein level and phosphosite level, proximal information is collected from the same sample in our spatially referenced proximity phosphoproteomics workflow, we combined both sets of quantitative data to visualise changes in subunit quantity and phosphorylation state at the kinetochore complex during cell cycle progression (Fig. 3). This permitted us to distinguish different classes of phosphosites, those that changed in abundance in a similar manner to the protein, and more dynamic phosphosites whose levels were not simply coupled to the amount of protein at the complex. Examples of the former class are KKT1(S1234) and KKT2(S25, S505-S506, S827 and S923-T928), as their abundance profile matched the protein profile. Examples of the latter class include KKT2(S493, S530) and KKT4(T120-S144, S300, T318-S328 and T421-T430). Because these phosphosites show dynamic behaviour de-coupled from the parent protein, we hypothesise that these sites may be particularly important in the regulation of kinetochore activity, for example, by controlling the recruitment of other proteins. We noted that for KKT7, KKT8 and KKT9 we could detect proximal phosphosites but not proximal protein in the ‘Total’ sample, indicating that these proteins are only enriched enough for their proximity to be detected after phosphopeptide purification.

A recent large-scale study of cell cycle-associated protein and phosphosite dynamics in synchronised *T. brucei* detected a total of 49 phosphosites on kinetochore proteins^23^. Our proximity phosphoproteomics detected 16 kinetochore phosphosites, this lower number reflecting the challenge of identifying phosphosites on the limited amount of material purified in proximity proteomics experiments. To identify phosphosites, which are conserved between *L. mexicana* and *T. brucei*, we performed multiple sequence alignments of KKTs detected in our proximity proteomics work (Supplementary Data 6). Within these KKTs, we have highlighted a total of 46 phosphosites identified by our work, the *T. brucei* dataset, or both. Of these phosphosites, we found that 19 (41%) were conserved between *L. mexicana* and *T. brucei*. The relatively low proportion of conserved phosphorylation sites is likely a consequence of the high degree of evolutionary plasticity at the primary sequence level between KKTs in kinetoplastid species^24^. Our proximity phosphoproteomics work identified seven of these conserved phosphosites: KKT1(S1234, S1449), KKT2(S25, S530, S923), KKT4(S300) and KKT7(S304), which may represent particularly important sites for regulation of kinetochore function.

### AB1 targets the redundant kinetochore protein kinases CLK1/CLK2 in *Leishmania* leading to defective cytokinesis

**Fig. 4:**
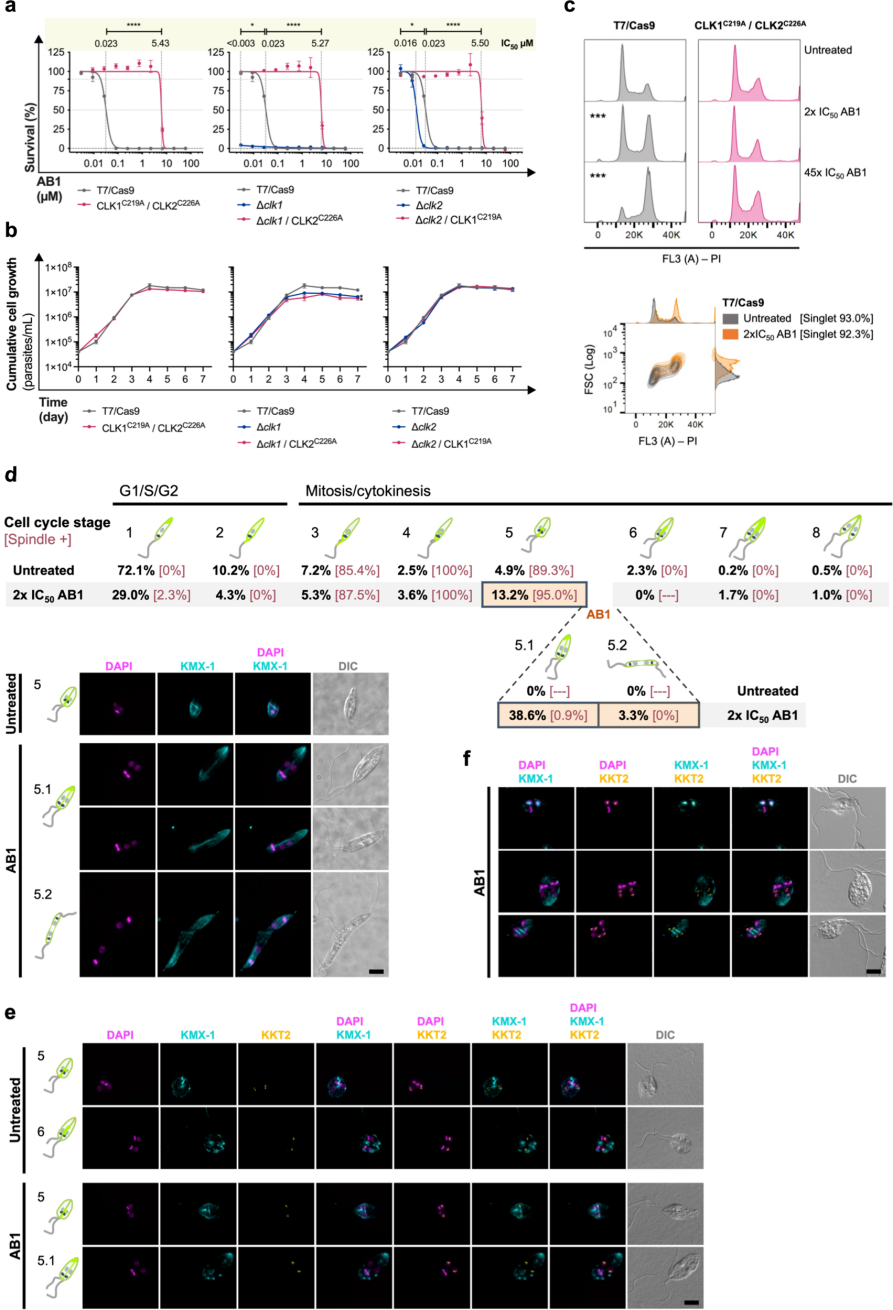
AB1 inhibits CLK1/CLK2 in *Leishmania* leading to defective cytokinesis. **a** CRISPR-Cas9-mediated precise genome editing was used to replace the C219 and C226 in *Leishmania* CLK1 and CLK2, respectively, with Alanine to assess AB1 specificity. The viability of the mutants and their progenitor lines after 96 h treatment with AB1 (0–60 µM) was assessed by measuring the conversion of resazurin (Alamar blue) to resorufin. Values are mean ± SEM. Fitting of dose-response curves was carried out using GraphPad Prism v7.0a, considering the untreated control for each cell line as 100% viability. AB1 half maximal inhibitory concentration (IC_50_) for each line is shown on the top. *P* values were calculated using unpaired two-tailed Student’s *t*-tests comparing each cell line with the T7/Cas9 progenitor. *p value <0.05 and ****p value <0.0001. T7/Cas9, progenitor line for CLK1^C219A^/CLK2^C226A^, Δ*clk1* and Δ*clk2*; Δ*clk1*, progenitor line for Δ*clk1*/CLK2^C226A^; and Δ*clk2*, progenitor line for Δ*clk2*/CLK1^C219A^. **b** Growth curve of *L. mexicana* promastigotes (mean ± SEM). The growth rate was calculated in the logarithmic area of the growth curve (0–96 h). The difference in growth rate was calculated using the non-parametric Mann–Whitney *U*-test, comparing the mutants with their progenitor line (*p value <0.05). **c** Representative cell cycle-profile histogram of cells stained with propidium iodide after 6 h of the treatment course with 2x IC_50_ and 45x IC_50_ AB1. Untreated parasites cultured for the same time were used as control. The FlowJo v.10.6.2 cell cycle algorithm Watson model was used to measure the percentage of cells in each cell cycle stage. *P* values were calculated using two-tailed Student’s *t*-tests comparing G2/M percentage with untreated control: ***p < 0.001. Bottom panel, adjunct histograms of cell cycle-profile and forward scatter (FSC) to analyse cell size throughout different cell cycle stages. **d** Fluorescence microscopy of promastigotes stained with KMX-1 to recognise β-tubulin and counterstained with DAPI to visualise DNA. Top panel, schematic of kinetoplast/nucleus configuration and β-tubulin distribution through the cell cycle in *L. mexicana*. Percentage of cells in each stage [percentage of cell with mitotic spindle] were measured for parasites growing in the presence or absence of AB1 after 6 h treatment (*n* > 300 cells). Bottom panel, representative fluorescence micrographs showing parasites in anaphase (5) for untreated control and cells with defective cytokinesis (5.1 and 5.2) after AB1 treatment. **e** Localisation of the inner kinetochore protein KKT2 after CLK1/CLK2 inhibition by AB1 in promastigotes. Representative fluorescence microscopy micrographs showing parasites endogenously expressing KKT2::mNG, stained with KMX-1 and counterstained with DAPI. Cells in anaphase (5), telophase (6) and with defective cytokinesis (5.1) are shown for parasites grown in the presence or absence of AB1 after 6 h treatment. **f** Multinucleated cells were observed after longer course CLK1/CLK2 inhibition (24 h). Representative fluorescence microscopy micrographs showing parasites endogenously expressing KKT2::mNG, stained with KMX-1 and counterstained with DAPI. Scale bars, 5 µM. DIC, the Nomarsky differential interference contrast.

The recently discovered kinase inhibitor AB1 is a potent growth inhibitor of *T. brucei* that was shown to covalently react with C215 in the ATP-binding pocket of CLK1^7^. Our cell viability assay in *L. mexicana* promastigote stage showed that *CLK1* or *CLK2 -*deficient mutants (Δ*clk1* and Δ*clk2* respectively^4^) are significantly more susceptible to AB1 than the T7/Cas9 progenitor, pinpointing CLK1 and CLK2 as AB1 targets in *Leishmania* (Fig. 4a). To verify if AB1 also binds to the equivalent cysteine residue in *Leishmania* CLK1 and CLK2 as *T. brucei* CLK1^C215^ these residues were identified (CLK1^C219^ and CLK2^C226^) and CRISPR-Cas9-mediated precise genome editing was then employed to replace the cysteine codon with an alanine codon. This mutation was also introduced in the Δ*clk1* and Δ*clk2* lines to investigate further AB1 targeting of these redundant protein kinases. Although Δ*clk1* and Δ*clk1* / CLK2^C226A^ lines showed a small but significantly lower growth rate when compared with T7/Cas9, no difference was observed between Δ*clk1* and Δ*clk1* / CLK2^C226A^ lines, suggesting that the replacement of cysteine by alanine does not cause loss of fitness for the parasite (Fig. 4b). AB1 target specificity to CLK1 and CLK2 was established by the refractory phenotype displayed by the mutant lines, revealing that replacement of the cysteine in the ATP-binding domain prevented binding of the covalent Michael-acceptor in AB1. It was observed that replacement with Alanine in just one of the genes is enough to make a parasite ~230 times more resistant than T7/Cas9 progenitor (Fig. 4a). Although it was observed that CLK1 deletion increases parasite susceptibility to AB1 more than CLK2 deletion, the refractory phenotype of the Δ*clk1* / CLK2^C226A^ mutants shows that CLK2 is also inhibited by AB1.

Once we confirmed the specificity of AB1 against CLK1/CLK2 we used this compound to perform chemical inhibition and study the biological role(s) of these kinases in *Leishmania*. The effect of CLK1/CLK2 inhibition with 2x IC_50_ AB1 on cell cycle progression was then examined, and a significant G2/M arrest was detected after 6 h treatment, which was not observed in the CLK^C219A^/CLK2^C226A^ line (Fig. 4c top). Cells arrested in G2/M under AB1 treatment were bigger than G2/M cells from untreated control (Fig. 4c bottom). We next used fluorescence microscopy with an antibody that recognises the mitotic spindle (KMX-1) to identify cells at different stages of the cell cycle (Fig. 4d, stage 1–8) and investigated further the cells arrested in G2/M due to CLK1/CLK2 inhibition. We observed that CLK1/CLK2 inhibition led to the accumulation of cells (36%) with segregated nuclei and kinetoplasts that were blocked in cytokinesis (Fig. 4d, stage 5.1). It is noteworthy that spindle formation was not disrupted upon CLK1/CLK2 inhibition. A small number of aberrant cells (3%) with segregated nuclei and kinetoplasts, but with a more elongated body and with the duplicated flagella positioned at opposite ends of the cell, was also observed (Fig. 4d, stage 5.2). Together, these results suggest that CLK1/CLK2 activity is essential for the completion of cytokinesis in *Leishmania*.

Inhibition of CLK1 in *T. brucei* causes dispersal of the canonical centromere kinetochore protein KKT2 foci, leading to disruption of kinetochore function^25^. To assess if this is also the case in *Leishmania*, we endogenously C-terminally tagged both alleles of KKT2 with mNeonGreen and checked its localisation in the presence and absence of AB1. The defined KKT2 foci remained unchanged after CLK1/CLK2 inhibition in *Leishmania* (Fig. 4e and Supplementary Fig. 8). Moreover, after 24 h treatment with AB1, most cells were multinuclear (≥4 nuclei): some with aneuploidy and some with the mitotic spindle, indicating they carried out further cycles of organelle replication despite the disruption of cytokinesis (Fig. 4f).

### Spatially referenced proximity phosphoproteomics and chemical inhibition reveals CLK1/CLK2-dependent phosphorylation at the kinetochore

**Fig. 5:**
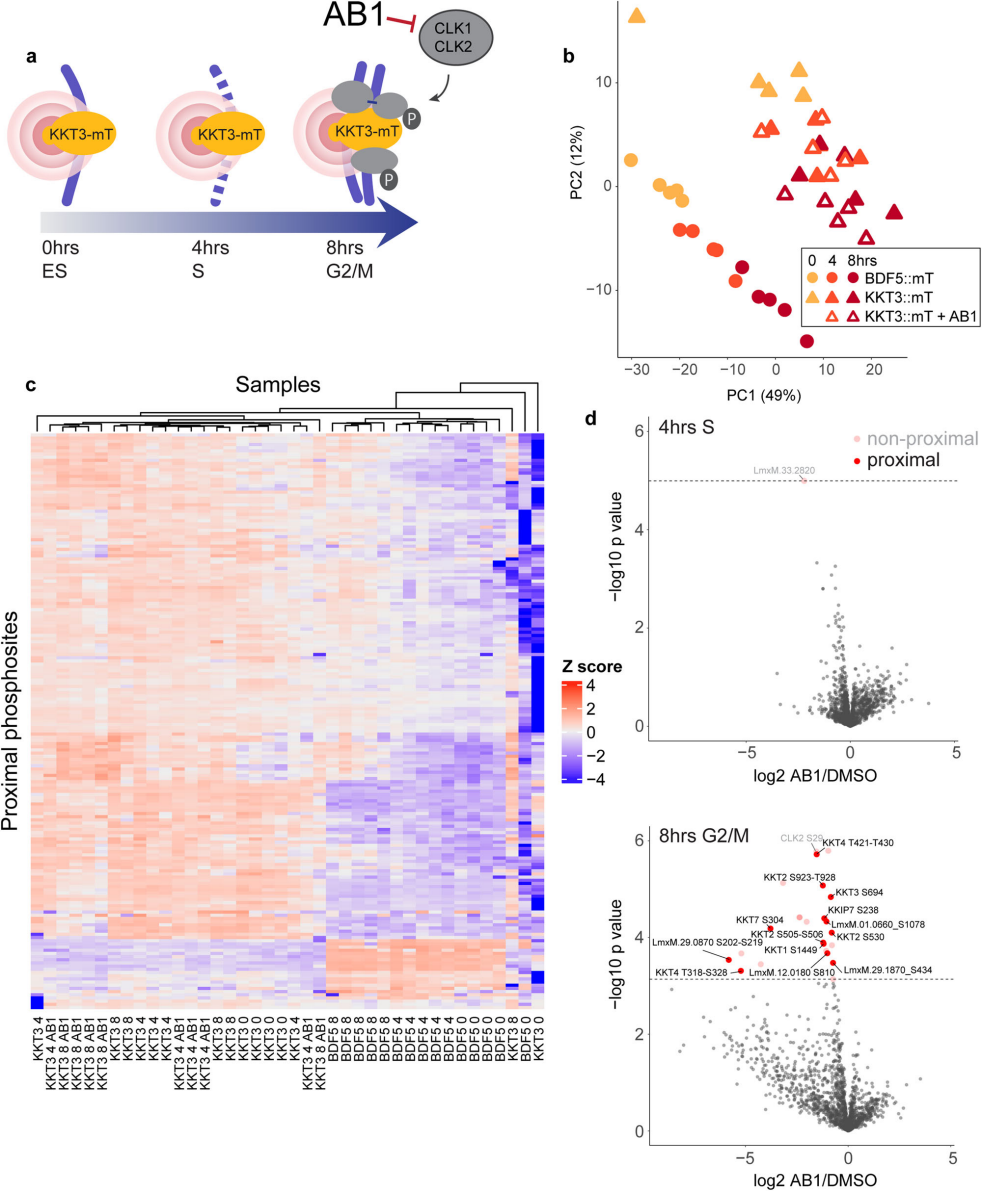
Kinetochore proximal phosphosites reduced after AB1 inhibition of the kinetochore kinases CLK1/CLK2. **a** Spatially referenced proximity phosphoproteomics was used as in Fig. 2 but with AB1 treatment from 0 h after synchronisation release. **b** Principal component analysis (PCA) of phosphopeptides quantified by spatially referenced proximity phosphoproteomics. PCA was performed on label-free quantified phosphopeptides with an ANOVA *q* value <0.1. **c** Heatmap of 191 proximal phosphosites. Each row is a phosphosite proximal to KKT3 or the spatial reference BDF5 in at least one timepoint at 5% FDR. Phosphosites are clustered by label-free abundance similarity across the samples. Samples are also clustered by similarity. **d** Label-free quantification of all phosphopeptides identified in AB1-treated vs. DMSO-treated parasites at 4 h after AB1 treatment and synchronisation release (top) and 8 h (bottom). Significantly changing phosphosites after AB1 treatment are either non-proximal to KKT3 (pink) or proximal to KKT3 (red). The dashed line indicates 5% FDR, *n* = 5.

CLK1/CLK2 localise to kinetochores and can phosphorylate kinetochore proteins in vitro^6^. Having demonstrated the target specificity of AB1 in *Leishmania*, we sought to use it in our spatially referenced proximity phosphoproteomics workflow, to uncover CLK1/CLK2-dependent signalling pathways at the kinetochore. To do this, parasites expressing miniTurbo-tagged KKT3 were synchronised with hydroxyurea, released and treated with 2x IC_50_ AB1 or DMSO. A 30 min biotinylation was carried out at 0, 4 and 8 h after hydroxyurea release and proximal proteins and phosphosites were quantified as before (Fig. 5a and Supplementary Data 4,5). Principal component analysis (PCA) of quantified phosphosites shows two well-separated groups of samples, demonstrating that the miniTurbo-tagged spatial reference BDF5 and miniTurbo-tagged KKT3 sample a different set of proximal phosphosites (Fig. 5b). Each of these groups is further divided into three distinct groups corresponding to the three timepoints in the cell cycle, AB1-treated samples cluster closely with corresponding untreated samples indicating that only a subset of identified phosphosites were affected by AB1. Clustering BDF5 or KKT3 proximal phosphosites by their intensity profiles across the samples also shows that the phosphorylation ‘landscape’ local to each protein is quite distinct, with a much larger group of phosphosites proximal to KKT3 (Fig. 5c).

**Fig. 6:**
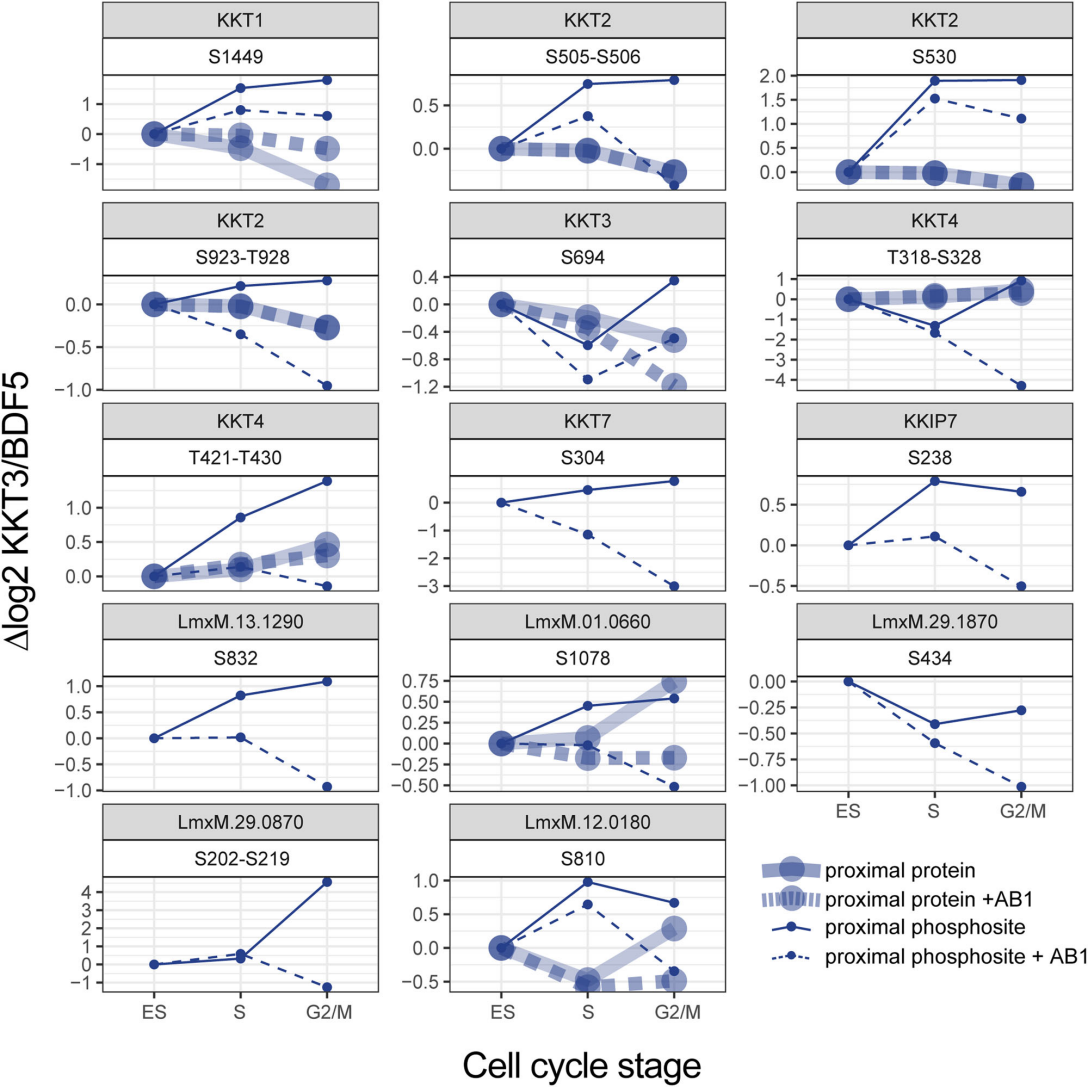
Proximal profile plots of AB1 responsive phosphosites and protein levels. At each timepoint, mean log2 fold enrichments against BDF5 spatial reference are normalised to the log2 fold enrichment at time 0, *n* = 5. Where a phosphosite could not be confidently localised, the possible phosphorylated region is indicated.

To reveal the individual proximal phosphosites affected by AB1 inhibition of CLK1/CLK2, the label-free intensity of phosphopeptides was compared between AB1-treated and DMSO-treated parasites at 4 and 8 h. At 4 h, only one phosphosite on a regulatory subunit of protein kinase A (LmxM.33.2820) was reduced. But by 8 h, a set of phosphosites that decreased after AB1 treatment became apparent (Fig. 5d). Because in these inhibition experiments, we record an additional layer of information, the proximity to KKT3, we further screened AB1 affected phosphosites to determine those that are at or near the kinetochore. Spatially referenced proximity phosphoproteomics allowed us to define 13 proximal phosphosites reduced by AB1, nine of which were on the kinetochore itself (Fig. 6). The most statistically significant decrease in phosphorylation occurred at S29 on CLK2, however, this phosphosite was not sufficiently enriched in the KKT3 sample, relative to the BDF5 spatial reference, to be classed as proximal. Of the proximal phosphosites affected by AB1, 3 occurred on KKT2, (S505, S530, S923-T926), which is a known substrate of CLK1/CLK2 in *T. brucei*^25^, two occurred on KKT4 and one on KKT7, which have both previously been shown to be phosphorylated in a CLK1/CLK2-dependent manner in *T. brucei*^6^. For the significantly reduced proximal phosphosites, we determined if there was any change in the levels of the corresponding proximal protein. KKT2 levels at the kinetochore were unchanged by AB1 treatment, which agreed with our analysis of KKT2 foci by microscopy. Thus, phosphosite decreases, in this case, reflect reductions in the phosphorylation state of kinetochore localised protein. Proximal KKT4 protein levels also do not change significantly after AB1 treatment, but 2 phosphosites, at T318-S328 and T421-T430, decrease significantly. Phosphorylation of KKT1 at S1449 is decreased after AB1 treatment, however proximal KKT1 levels are significantly increased, thus together indicating a large decrease in the proportion of S1449 phosphorylated KKT1 at the kinetochore. We observed that AB1 caused a decrease in levels of KKT3 by 0.63-fold at 8 h and a similar decrease in S694 phosphorylation. Since this is the bait protein and is attached to the biotin ligase, biotinylation and enrichment of KKT3 itself is not dependent on proximity within the kinetochore. Thus, both free and kinetochore-associated KKT3 are biotinylated and enriched. AB1 caused a decrease in levels of KKT3, but not levels of other kinetochore members, KKT1, KKT2 or KKT4. We, therefore, conclude that levels of KKT3-miniTurbo at the kinetochore carrying out proximity labelling are not significantly affected by AB1.

### KKT24 and KKT26 are novel essential *Leishmania* kinetochore components

**Fig. 7:**
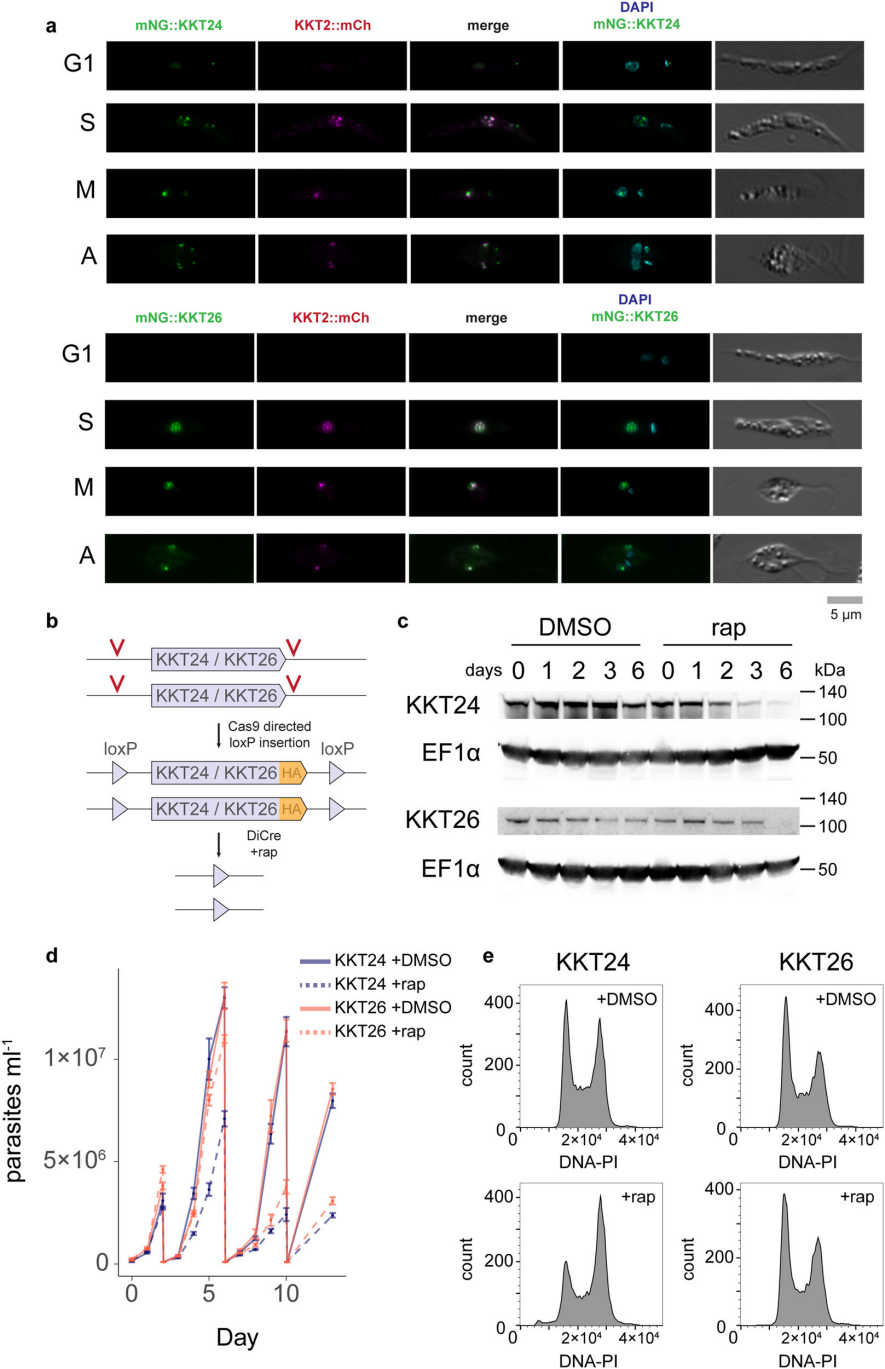
Localisation and essentiality of KKT24 and KKT26, novel essential components of the *Leishmania* kinetochore. **a** Fluorescence co-localisation microscopy of KKT24 (LmxM.34.0180) or KKT26 (LmxM.17.0650) with KKT2. KKT24 and KKT26 were mNeonGreen (mNG) tagged and localisations compared to mCherry (mCh) tagged KKT2 at different phases of the cell cycle: G1, S, M (metaphase), A (anaphase). **b** Strategy for inducible deletion of *KKT24* or *KKT26*. Homology-directed repair after Cas9-induced breaks was used to insert LoxP sites flanking the endogenous genes at both alleles. Addition of rapamycin-induced dimerisation of DiCre recombinase, which excised *KKT24* or *KKT26*. **c** Depletion of C-terminally HA-tagged KKT24 or KKT26 following gene excision, shown by Western blotting using an anti-HA antibody. EF1ɑ was used as a loading control. **d** Parasite growth after KKT24-HA or KKT26-HA excision. Parasites were diluted to 1 × 10^6^ ml^−1^ at days 2, 6 and 10 after the addition of rapamycin (rap) or DMSO, median and standard deviation of four replicates are shown. **e** Cell cycle profile of parasites after KKT24-HA or KKT26-HA excision. Parasites 6 days after the addition of rapamycin or DMSO were stained with propidium iodide for DNA content and analysed by flow cytometry.

Our XL-BioID analysis of the *Leishmania* kinetochore protein kinases identified a group of unknown proximal proteins. To determine if any of these were previously undescribed components of the kinetoplastid kinetochore, we endogenously tagged uncharacterised proteins with mNeonGreen and performed a fluorescence co-localisation screen in parasites expressing mCherry KKT2. We screened a total of 25 uncharacterised proteins, 24 of these were found to be predominantly localised to the nucleus indicating a good level of compartment specificity in the XL-BioID experiment (Supplementary Figs. 9–15). Amongst the nuclear localised proteins, we observed two proteins, LmxM.34.0180 and LmxM.17.0650, which formed sets of multiple nuclear punctae in the S phase, a localisation pattern that is characteristic of kinetochore proteins. The punctae then appear to coalesce into mostly individual puncta at metaphase and anaphase (Fig. 7a), co-localising with KKT2 across the cell cycle. LmxM.34.0180 has since been identified as an orthologue of kinetochore protein KKT24 in *Trypanosoma brucei*^3^ and we designate LmxM.17.0650 as a novel kinetochore protein KKT26. Interestingly, KKT24 also localises to the kinetoplast, a specialised region containing the mitochondrial DNA.

Attempts to delete *KKT24* or *KKT26* using CRISPR- Cas9 in *L. mexicana* promastigotes were unsuccessful, indicating these kinetochore proteins are essential to parasite survival. We, therefore, opted for an inducible gene deletion strategy, using Cas9-directed insertion to introduce loxP sites flanking the endogenous genes^26^. The addition of rapamycin induces dimerisation of DiCre recombinase, which then excises the loxP-flanked gene (Fig. 7b and Supplementary Figs. 16, 17). We monitored protein levels after gene excision and found that KKT24 became depleted from day 3 post-induction whilst KKT26 levels depleted more slowly (Fig. 7c and Supplementary Fig. 18). We found that while both proteins are required for parasite growth, the onset of growth inhibition was earlier after KKT24 excision, matching the more rapid protein depletion kinetics compared to KKT26 (Fig. 7d). Because the kinetochore is essential for chromosome segregation and normal cell cycle progression, we measured DNA content of parasites by flow cytometry 6 days after KKT24 or KKT26 excision. Deleting KKT26 did not lead to defects in the cell cycle profile; by contrast, deletion of KKT24 caused parasites to accumulate in the G2/M phase and generated parasites containing sub-genome equivalents of DNA (Fig. 7e).

## Discussion

The segregation of duplicated chromosomes during mitosis, driven by kinetochore-mediated attachment of chromosomes to the mitotic spindle, is a tightly orchestrated sequence of events. This reduces the possibility of errors occurring that would ultimately lead to an incorrect complement of genetic material being segregated into daughter cells, with potentially catastrophic effects. Kinetochore-mediated chromosome segregation must therefore integrate seamlessly into the wider events of the cell cycle^27^. The role of the kinetochore as a signalling hub plays a central part in this co-ordination. In mammals, a network of protein kinases (e.g. Aurora B and MPS1) and phosphatases (PP1 and PP2) monitor the sequence of molecular steps at the kinetochore^28^. A precise balance between kinase and phosphatase activity sets a threshold that determines whether the next stage should be attempted or whether the cell cycle should be paused to allow any errors to be corrected, such as defective kinetochore-microtubule attachments. Here we have applied an optimised proximity biotinylation approach (XL-BioID) and spatially referenced proximity phosphoproteomics with chemical inhibition (AB1), to examine the role of protein kinases at the *Leishmania* kinetochore and understand the consequences of disturbing this balance at the *Leishmania* kinetochore during the cell cycle.

Our XL-BioID results indicate that KKT2 and KKT3 form a core part of the *Leishmania* kinetochore, as we were able to proximity capture most of the expected kinetochore subunits using these protein kinases as baits. The cdc2-like kinases CLK1 - and CLK2 - associate with kinetochores more transiently during the cell cycle, associating at S phase and dissociating at anaphase in trypanosomes^6^, probably leading to the more limited coverage of kinetochore subunits by XL-BioID when CLK2 was used as bait. Whilst at the sequence level, the kinetoplastid kinetochore appears quite distinct^2^, our proximity map of the kinetochore revealed several expected proteins and complexes normally associated with chromosome segregation in other eukaryotes, such as the chromosome passenger complex, condensin and cohesin, suggesting there are at least some molecular processes common to eukaryotes governing the separation of chromosomes in *Leishmania*.

The two novel subunits of the kinetochore revealed by XL-BioID, KKT24 and KKT26, do not have detectable sequence similarity with kinetochore components outside the kinetoplastids, further supporting the uniqueness of the kinetoplastid kinetochore complex. In common with many of the kinetoplastid kinetochore components, these new subunits do not have any predicted protein domains. KKT24 is a large protein and is predicted to fold into a coiled-coils tertiary structure which may give it load-bearing strength^3^. It could therefore form part of a complex with a role similar to that of the ndc80 complex, which is formed from coiled-coils containing proteins and links the inner kinetochore to the mitotic spindle^29^. The kinetoplastid kinetochore has now been interrogated by numerous immunoprecipitation and proximity biotinylation experiments which have likely identified the majority, if not all, the core components^2,3,22,30,31^. It will now be important to carry out structural studies to understand the architecture of the kinetoplastid kinetochore and how this underpins the mechanism of chromosome segregation.

**Fig. 8:**
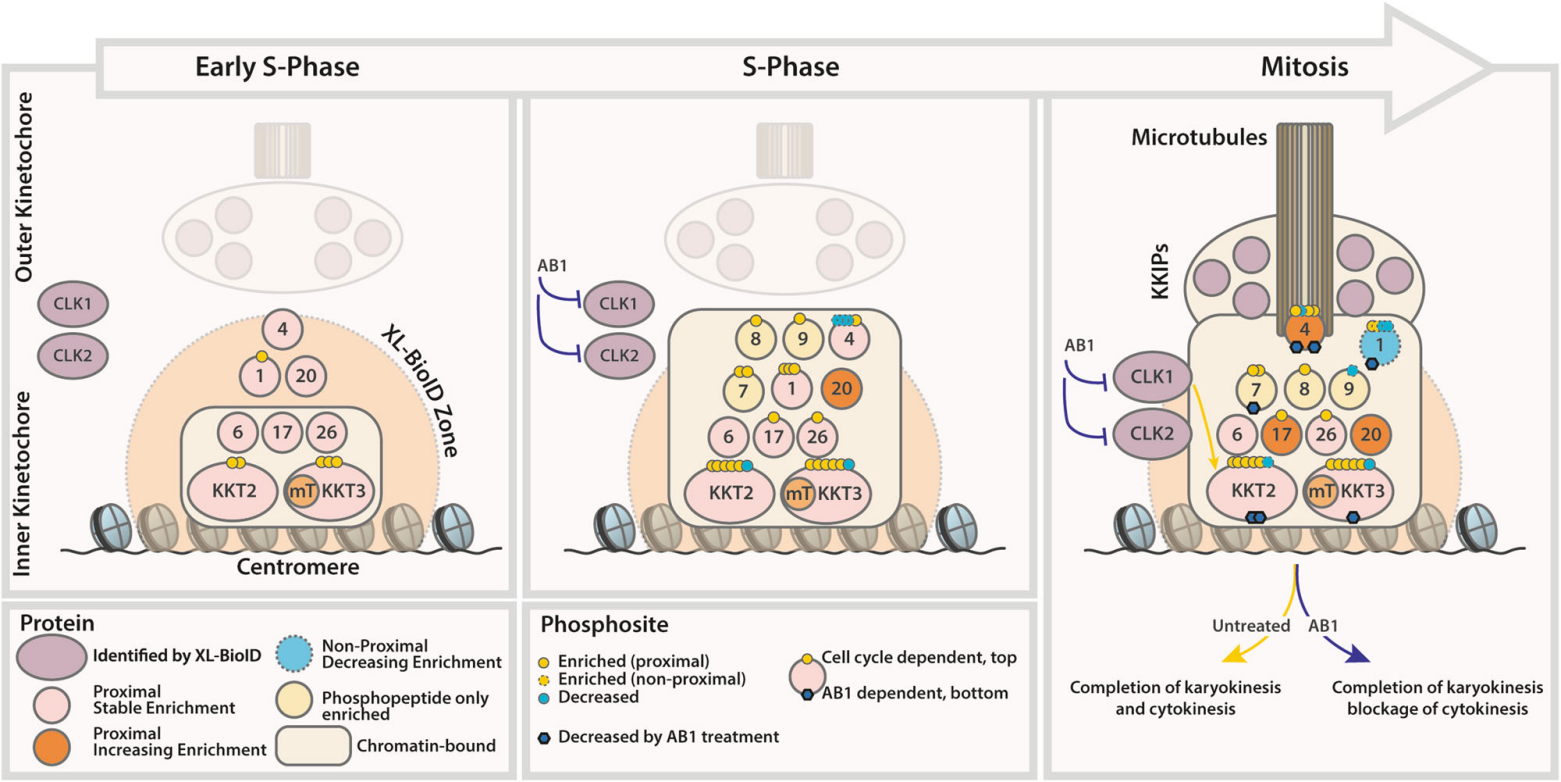
Schematic representation of *Leishmania* kinetochore assembly and effect of CLK1/CKL2 inhibition by AB1, as inferred from XL-BioID proximity biotinylation data. MiniTurbo-tagged KKT3 (KKT3-mT, the bait) detects a core cohort of kinetochore subunits already present in the early S phase, which likely form part of the inner kinetochore bound to centromeric chromatin. Of these subunits, only KKT1 and KKT2 have detectable phosphorylation. Progression to S phase sees a large increase in phosphorylated kinetochore subunits and increasing enrichment of KKT4 and KKT20, but no detectable impact of CLK1/CLK2 inhibition by AB1. In mitosis, the kinetochore is fully formed and attached to microtubules of the mitotic spindle, possibly via KKT4, which has reached peak enrichment. At this stage, the consequences of inhibiting CLK1/CLK2 activity are detected as a reduction in phosphorylation of kinetochore subunits KKT1, KKT2, KKT3 and KKT4. Reduced phosphorylation of KKT7 is also observed, but we cannot exclude the possibility that this is due to reduced recruitment to the kinetochore. AB1-treated *Leishmania* can continue to complete nuclear division (karyokinesis) but are then arrested at cell division (cytokinesis).

We were able to follow the *Leishmania* kinetochore by proximity biotinylation as it assembles during the cell cycle by using a faster biotin ligase variant. We discovered a set of proteins already present early in the cell cycle, which may be analogous to the constitutive centromere-associated network (CCAN) in other eukaryotes. Some of the *Leishmania* kinetochore components, such as KKT4 and KKT20 are recruited in increased amounts as the cell cycle proceeds and could be important for the attachment of the kinetochore to the mitotic spindle. The mammalian kinetochore is a dynamic complex whose assembly during the cell cycle is directed by key phosphorylation events that control the assembly of modules (e.g. CCAN-KMN), as well as modulating the configuration of proteins within modules (e.g. CENP-C in the CCAN)^32,33^. Our spatially referenced proximity phosphoproteomic analysis indicates that the *Leishmania* kinetochore undergoes significant changes in phosphorylation of inner kinetochore proteins KKT1, KKT2, KKT3 and KKT4 during the cell cycle (Fig. 8). Because two of the dynamically phosphorylated proteins, KKT2 and KKT3, are themselves essential protein kinases, some of these phosphorylation events may be regulators of kinetochore assembly and function.

If kinetochores fail to attach properly, there is a risk that chromosomes will not be partitioned equally into daughter cells. The role of the spindle assembly checkpoint (SAC) in other eukaryotes is to check for kinetochore-spindle attachments and raise a ‘stop’ signal to delay mitotic progression until any attachment errors have been resolved. Kinetoplastids appear to lack homologues for the key SAC kinases Mps1 and Bub1, yet there is likely a signalling mechanism at the kinetochore to monitor attachment to the spindle. CLK1/CLK2 may form part of this signalling pathway since inhibition of CLK1 activity in trypanosomes leads to mitotic arrest and CLK1/CLK2 knockdown leads to delayed metaphase to anaphase progression^6,7^. However, we show here that CLK1/CLK2 inhibition by AB1 does not block spindle formation in *Leishmania*, which corroborates the findings in *T. brucei* when CLK1 was chemically inhibited or knocked down^7,25^. Our spatially referenced proximity phosphoproteomics experiments uncovered a subset of phosphorylation sites at the kinetochore impacted by AB1 inhibition of CLK1/CLK2; these changes occurred later on in the cell cycle during M phase (Fig. 8). Interestingly, AB1 treatment also decreased KKT3 levels, which may have been caused by an increase in turnover or decrease in the synthesis of KKT3, whilst the level of KKT3 associated with the kinetochore appeared unaffected. At the cellular level, we found that phosphorylation defects at the kinetochore did not affect the KKT2 foci pattern, but were associated with cytokinesis failure in parasites. *Leishmania* appears to be able to continue in the cell cycle after CLK1/CLK2 inhibition until the point of cytokinesis (Figs. 3,6), as well as going through further nuclear division cycles in the non-divided cell.

CLK1/CLK2 are identical in sequence across the kinase domain, only differing at their N-termini. They appear to be functionally redundant in procyclic but not bloodstream form *T. brucei*^6,34^. Our results indicate that while CLK1/CLK2 are functionally redundant in *Leishmania*, differences in AB1 sensitivity between CLK1 or CLK2 deletion strains suggest that CLK1 may be the more functionally dominant kinase at the kinetochore under normal conditions. CLK2 can replace functions in the absence of CLK1, but in this state, parasites become extremely sensitive to the inhibitor AB1.

In animal cells, malfunction of kinetochores can cause cytokinesis failure, either by physically blocking the formation of the ingressing furrow due to lagging chromosomes, or by activation of the abscission checkpoint^35^. The abscission checkpoint, also called the ‘NoCut’ pathway, is an Aurora B kinase-driven signalling pathway that delays cytokinesis. Incompletely segregated chromosomes cause CLKs to activate Aurora B kinase, which then phosphorylates and blocks abscission factors from operating. CLKs act as upstream activators of Aurora B and their activity is thus essential for the operation of the abscission checkpoint^35^. The blockage of cytokinesis in *Leishmania* when CLK1/CLK2 are inhibited could be due to physical defects or activation of a distinct abscission checkpoint pathway which does not require the activity of CLK1/CLK2.

Overall, we have characterised the components and dynamics of the *Leishmania* kinetochore using a sensitive proximity biotinylation method, discovered two novel essential components and gained detailed insight into the phosphosite-level impact of the promising anti-parasitic compound AB1 on the kinetochore.

## Methods

### Cell culture

*Leishmania mexicana* (MNYC/BZ/62/M379) promastigotes were cultured at 25 °C in HOMEM medium (modified Eagle’s medium) supplemented with 10% (v/v) heat-inactivated foetal calf serum (GIBCO) and 1% Penicillin/Streptomycin (Sigma-Aldrich). CRISPR-cas9 edited parasites were supplemented with the appropriate selection antibiotics: BirA*::BDF5, KKT2::BirA*, BirA*::KKT3 or CLK2::BirA*: 4 μg ml^−1^ puromycin (Invivogen), BDF5::mT and KKT3::mT: 4 μg ml^−1^ puromycin, 10 μg ml^−1^ blasticidin, loxP-flanked KKT24, KKT26: 4 μg ml^−1^ puromycin, 10 μg ml^−1^ blasticidin, 15 μg ml^−1^ G418, 32 μg ml^−1^ hygromycin B, mNG::KKT24, mNG::KKT26 KKT2::mCh: 4 μg ml^−1^ puromycin, 10 μg ml^−1^ blasticidin.

### Growth curve

The promastigote form of *L. mexicana* was set up at 4 × 10^4^ parasites mL^−1^ in HOMEM medium supplemented with 10% heat-inactivated FBS, and the cumulative cell growth was measured daily by cell counting in a Neubauer chamber. The growth rate was calculated in the logarithmic area of the growth curve (0–96 h).

### Generation of CRISPR-cas9 edited lines

Primers used to generate sgRNA expression cassettes and repair cassettes were designed using http://www.leishgedit.net^9^ or manually for the loxP lines^26^ and are reported in Supplementary Data 7. Cassettes were generated by PCR, precipitated with glycogen, resuspended in 10 μl water for each transfection and heat sterilised for 5 min at 95 °C. About 2 μl of this was then used to transfect 1 × 10^6^ promastigotes using a 4D nucleofector (Lonza). Parasites used for transfections were: Cas9 T7^9^ and DiCre Cas9 T7^36^. Eighteen hours after transfection, selection antibiotics were added and lines were cloned by limiting dilution. The KKT2::mCh line was generated by C-terminally tagging endogenous KKT2 in Cas9 T7 according to the procedure outlined above but with puromycin selection on a population of parasites. The mNG::KKT24 and mNG::KKT26 lines were generated by endogenously tagging genes at the N-terminus in Cas9 T7 with blasticidin selection on a population. Lines were verified by western blot and/or diagnostic PCR. For loxP lines, clones were also screened for excision efficiency by diagnostic PCR of genomic DNA. For Cas9-mediated precise genome editing, mutants were generated by replacing the target codon encoding cysteine with an alanine residue^37^. The validation of the mutants was performed by Sanger sequencing. Oligonucleotide sequences are provided in Supplementary Data 7.

### Parasite susceptibility to AB1

A dose-response curve was set with 1 × 10^4^ parasites mL^−1^ treated with the AB1 in a range concentration varying from 0 to 100 μM, in a 96-well plate. The viability of treated and untreated control was assessed after 96 h by the addition of 50 µL of 0.0125% (w/v) resazurin prepared in PBS. Cells were incubated for an additional 2–4 h at 37 °C. Fluorescence emission of the reduced resazurin was detected using a CLARIOstar^®^ reader (BMG LABTECH; excitation filter at 540 nm and emissions filter at 590 nm). Fitting of dose-response curves and IC_50_ calculation were carried out using GraphPad Prism v.9.3.1, considering the untreated control for each cell line as 100% viability.

### Cell cycle analysis

Promastigote cells growing in the presence or absence of AB1 for 6 h were washed in PBS-EDTA (PBS supplemented with 5 mM of EDTA) and resuspended in 70% methanol. After overnight incubation at 4 °C, cells were washed once with PBS-EDTA and then resuspended in 1 ml PBS-EDTA containing 10 µg mL^−1^ of propidium iodide and 10 µg mL^−1^ of RNase A. Cells were incubated for 45 min at 37 °C in the dark until FACS analysis. Cells were analysed for FACS using CyAn^TM^ cytometer (Beckman Coulter) and the data were analysed using FlowJoTM 10.6.2 cell cycle algorithm: Watson model.

### Immunofluorescence

KKT2::mCh, mNG::KKT24 and mNG::KKT26 were washed once in PBS before fixation with 1% paraformaldehyde (PFA) for 10 min at RT. PFA was quenched with 0.5 M Tris pH8.5 for 5 min at RT. About 1 × 10^6^ parasites were prepared for each line. Parasites were allowed to adhere to microscope slides (SuperFrost, Thermo Scientific) for 15 min, permeabilised with 0.5% Triton X-100 for 10 min and blocked with 5% FBS in PBS for 15 min. Anti-mCherry antibody (mCherry Monoclonal Antibody 16D7, Alexa Fluor 647, Invitrogen) at 1:50 in 5% FBS in PBS was added for 1 h at RT. Parasites were washed 3x in PBS for 5 min each wash before mounting in ProLong Diamond (Thermo scientific). Images were acquired on an inverted Zeiss AxioObserver with a 100x lens. Image Z-stacks were blind deconvolved with the Microvolution plugin for Fiji using 100 iterations.

Mitotic spindle staining: promastigotes grown in the presence or absence of AB1 (2x IC_50_, 60 nM) for 6 or 24 h were washed twice with PBS (1400×*g* for 10 min at room temperature). About 10^6^ cells in PBS were left to adhere to a poly-l-lysine pre-treated coverslip for 15 min at 37 °C. The cell line endogenously expressing KKT2 was submitted to an additional incubation step with 1 mM DSP/PBS for 10 mins at 37 °C to perform in vivo cross-linking. The attached parasites were then fixed for 15 min, at room temperature (RT), in 4% paraformaldehyde/PBS, followed by 5 min incubation with 0.1 M glycine/PBS, pH 7.6, to quench the fixation. The fixed cells were washed twice with PBS and then permeabilized with 0.5% Triton X-100/PBS for 15 min. Cells were then incubated with a blocking buffer (5% bovine serum albumin, 0.01% saponin in PBS) for 1 h. Cells were immune-stained for 1 h with mouse anti- b-tubulin antibody clone KMX-1 (MAB3408, Sigma-Aldrich) diluted 1:1000 in the blocking buffer. After three washes with 0.1% Triton X-100/PBS, cells were incubated for 1 h with an Alexa Fluor 647-conjugated goat anti-mouse IgG (ab150119, Abcam, used at 1:2000 in blocking solution) secondary antibody. Following three washes with 0.1% Triton X-100/PBS, cells were counterstained with 20 µg mL^−1^ DAPI/PBS for 30 min and washed once with PBS. The slides were mounted in ProLong diamond antifade mountant (Invitrogen), following the manufacturer’s instructions. Images were acquired as mentioned above.

### XL-BioID

BirA*::BDF5, KKT2::BirA*, BirA*::KKT3, CLK2::BirA* parasites were grown to 4 × 10^6^ ml^−1^, at which point biotinylation was initiated in three replicates of each line, by adding biotin to 150 μM for 18 h. BDF5::mT and KKT3::mT parasites were grown to 2 × 10^6^ ml^−1^ and then synchronised by adding hydroxyurea to 0.4 mg ml^−1^ for 18 h^15^. Synchronised parasites were washed twice in a culture medium and resuspended at 4 × 10^6^ ml^−1^ to proceed with the cell cycle, five replicates of each line and condition were used. For inhibition experiments, AB1 was added to a concentration of 60 nM. Biotinylation timepoints were taken at 0, 4 and 8 h after hydroxyurea wash-off. At each timepoint, biotin was added to 0.5 mM for 30 min biotinylation and 4 × 10^6^ parasites were taken for flow cytometry analysis.

After in vivo biotinylation, parasites were washed twice in PBS and resuspended to a density of 4 × 10^7^ ml^−1^ in pre-warmed PBS. DSP crosslinker was added to 1 mM and in vivo cross-linking proceeded for 10 min at 25 °C. Cross-linking was quenched for 5 min by the addition of Tris-HCl pH 7.5 to a concentration of 20 mM. Parasites were harvested by centrifugation and pellets were stored at −80 °C until lysis. A pellet of 4 × 10^8^ parasites, corresponding to a total protein mass of 1.9 mg, was used for each affinity purification which was lysed in 500 μl ice-cold RIPA buffer containing 0.1 mM PMSF, 1 μg ml^−1^ pepstatin A, 1 μM E-64, 0.4 mM 1-10 phenanthroline. In addition, every 10 ml of RIPA (0.1% sodium dodecyl sulfate, 0.5% sodium deoxycholate, 1% IgePal-CA-630, 0.1 mM EDTA, 125 mM NaCl, 50 mM Tris pH 7.5) was supplemented with 200 μl proteolytic protease inhibitor cocktail containing w/v 2.16% 4-(2-aminoethyl)benzenesulfonyl fluoride hydrochloride, 0.047% aprotinin, 0.156% bestatin, 0.049% E-64, 0.084% Leupeptin, 0.093% Pepstatin A (Abcam), three tablets complete protease inhibitor EDTA free (Roche) and one tablet PhosSTOP (Roche). Lysates were sonicated with a microtip sonicator on ice for three rounds of 10 s each at an amplitude of 30. One microlitre of Benzonase (250 Units, Abcam) was added to each lysate, digestion of nucleic acids proceeded for 10 mins at RT followed by 50 mins on ice. Lysates were clarified by centrifugation at 10,000 g for 10 min at 4 °C. For enrichment of biotinylated material, 100 μl of magnetic streptavidin bead suspension (1 mg of beads, Resyn Bioscience) was used for each affinity purification from 4 × 10^8^ parasites. Biotinylated material was affinity purified by end-over-end rotation at 4 °C overnight. Beads were washed in 500 μl of the following for 5 min each: RIPA for four washes, 4 M urea in 50 mM triethyl ammonium bicarbonate (TEAB) pH8.5, 6 M urea in 50 mM TEAB pH8.5, 1 M KCl, 50 mM TEAB pH8.5. Beads from each affinity purification were then resuspended in 200 μl 50 mM TEAB pH8.5 containing 0.01% ProteaseMAX (Promega), 10 mM TCEP, 10 mM Iodoacetamide, 1 mM CaCl_2_ and 500 ng Trypsin Lys-C (Promega). The on-bead digest was carried out overnight at 37 °C while shaking at 200 rpm. Supernatant from digests was retained and beads were washed for 5 min in 50 μl water which was then added to the supernatant. Digests were acidified with trifluoroacetic acid (TFA) to a final concentration of 0.5% before centrifugation for 10 min at 17,000×*g*. The supernatant was desalted using in-house prepared C18 desalting tips, elution volume was 60 μl. Desalted peptides were either dried for MS analysis (BirA* experiments) or used for the enrichment of proximal phosphopeptides (miniTurbo experiments).

### Proximal phosphopeptide enrichment

Desalted peptides were pipette mixed to ensure a homogenous sample and 40% was removed and dried down for the ‘Total’ proximal protein sample. To the remaining 36 μl was added 51.2 μl acetonitrile, 10 μl 1 M glycolic acid and 5 μl TFA. Magnetic Ti-IMAC-HP beads (ReSyn Biosciences) were washed three times for 1 min in a 2x volume of loading buffer (0.1 M glycolic acid, 80% acetonitrile-ACN, 5% TFA). Ten microlitres of bead suspension (200 μg beads) were used to enrich phosphopeptides from each affinity purification. Peptides were added to beads and incubated with shaking at 800 rpm for 40 min at RT. Beads were washed for 2 min at 800 rpm in 100 μl loading buffer, then 100 μl 80% ACN 1% TFA and finally 100 μl 10% ACN 0.2% TFA. Phosphopeptides were eluted in 2 × 40 μl 1% NH_4_OH for 10 min shaking at 800 rpm. About 2 μl TFA was added and peptides dried down for MS analysis.

### Mass spectrometry data acquisition

For total proteome XL-BioID analysis, peptides were loaded onto an UltiMate 3000 RSLCnano HPLC system (Thermo) equipped with a PepMap 100 Å C_18_, 5 µm trap column (300 µm × 5 mm, Thermo) and a PepMap, 2 µm, 100 Å, C_18_ EasyNano nanocapillary column (75 mm × 500 mm, Thermo). Separation used gradient elution of two solvents: solvent A, aqueous 1% (v:v) formic acid; solvent B, aqueous 80% (v:v) acetonitrile containing 1% (v:v) formic acid. The linear multi-step gradient profile was: 3–10% B over 8 min, 10–35% B over 115 min, 35–99% B over 30 min and then proceeded to wash with 99% solvent B for 4 min. For analysis of samples prepared to measure dynamics of proximal proteins and phosphosites during kinetochore assembly, peptides were loaded onto an mClass nanoflow UPLC system (Waters) equipped with a nanoEaze M/Z Symmetry 100 Å, C18, 5 µm trap column (180 µm × 20 mm, Waters) and a PepMap, 2 µm, 100 Å, C18 EasyNano nanocapillary column (75 mm × 500 mm, Thermo). Separation used gradient elution of two solvents: solvent A, aqueous 0.1% (v:v) formic acid; solvent B, acetonitrile containing 0.1% (v:v) formic acid. The linear multi-step gradient profile for ‘Total’ protein was: 3–10% B over 8 min, 10–35% B over 115 min, 35–99% B over 30 min and then proceeded to wash with 99% solvent B for 4 min. For phosphopeptide enriched samples, the following gradient profile was used: 3–10% B over 7 min, 10–35% B over 30 min, 35–99% B over 5 min and then proceeded to wash with 99% solvent B for 4 min. In all cases, the trap wash solvent was aqueous 0.05% (v:v) trifluoroacetic acid and the trapping flow rate was 15 µL/min. The trap was washed for 5 min before switching flow to the capillary column. The flow rate for the capillary column was 300 nL/min and the column temperature was 40 °C. The column was returned to initial conditions and re-equilibrated for 15 min before subsequent injections.

The nanoLC system was interfaced with an Orbitrap Fusion hybrid mass spectrometer (Thermo) with an EasyNano ionisation source (Thermo). Positive ESI-MS and MS^2^ spectra were acquired using Xcalibur software (version 4.0, Thermo). Instrument source settings were: ion spray voltage, 1,900 V; sweep gas, 0 Arb; ion transfer tube temperature, 275 °C. MS^1^ spectra were acquired in the Orbitrap with 120,000 resolution, scan range: *m/z* 375–1500; AGC target, 4e^5^; max fill time, 100 ms. Data-dependent acquisition was performed in top speed mode using a fixed 1 s cycle, selecting the most intense precursors with charge states 2–5. Easy-IC was used for internal calibration. Dynamic exclusion was performed for 50 s post precursor selection and a minimum threshold for fragmentation was set at 5e^3^. MS^2^ spectra were acquired in the linear ion trap with: scan rate, turbo; quadrupole isolation, 1.6 *m/z*; activation type, HCD; activation energy: 32%; AGC target, 5e^3^; first mass, 110 *m/z*; max fill time, 100 ms. Acquisitions were arranged by Xcalibur to inject ions for all available parallelizable time.

### Mass spectrometry data analysis

Peak lists in.raw format were imported into Progenesis QI (Version 2.2., Waters) for peak picking and chromatographic alignment. A concatenated product ion peak list was exported in.mgf format for database searching against the *Leishmania mexicana* subset of the TriTrypDB (8,250 sequences; 5,180,224 residues) database, appended with common proteomic contaminants. Mascot Daemon (version 2.6.1, Matrix Science) was used to submit searches to a locally-running copy of the Mascot programme (Matrix Science Ltd., version 2.7.0.1). Search criteria specified: Enzyme, trypsin; Max missed cleavages, 2; Fixed modifications, Carbamidomethyl (C); Variable modifications, Oxidation (M), Phospho (STY), Acetyl (Protein N-term, K), Biotin (Protein N-term, K); Peptide tolerance, 3 ppm (# 13 C = 1); MS/MS tolerance, 0.5 Da; Instrument, ESI-TRAP. Peptide identifications were passed through the percolator algorithm to achieve a 1% false discovery rate as assessed empirically by reverse database search, and individual matches were filtered to require minimum expected scores of 0.05. The Mascot.XML results file was imported into Progenesis QI, and peptide identifications associated with precursor peak areas were mapped between acquisitions. Relative protein abundances were calculated using precursor ion areas from non-conflicting unique peptides. For total proteome data, only non-modified peptides were used for protein-level quantification. Normalisation and statistical testing was performed in Progenesis QI, with the null hypothesis being peptides are of equal abundance among all samples^38^. ANOVA-derived *p* values from QI were converted to multiple test-corrected *q* values within QI. For phosphopeptide identifications, Mascot-derived site localisation probabilities were used. To associate quantification data with site localisation probabilities, Mascot search results, including raw spectral details and QI quantification data were exported separately in.csv format. Empty rows in the Mascot.csv were removed in R (3.6.1) before joining the data from the two.csv files using KNIME Analytics Platform (4.3.1 KNIME AG). The combined.csv was stripped of non-quantified peptides before calculating Hochberg and Benjamini FDR *q* values from the original QI ANOVA *p* values.

For BirA* experiments, normalised protein label-free peak areas were exported from Progenesis LFQ and those proteins with at least two unique peptides identified were retained for downstream analysis. Missing values were imputed by drawing values from a left-shifted normal log2 intensity distribution to model low abundance proteins (mean = 14, sd = 1.2). Proximal proteins were determined with the limma package^39^ using options trend = TRUE and robust = TRUE for the eBayes function. Multiple testing correction was carried out according to Benjamini & Hochberg, the false discovery rate for identified proximals was 1%.

For miniTurbo total proximal data, peptide ion quantification data were exported from Progenesis LFQ and peptides with ≥2 missing values across the five biological replicates were removed. Missing value imputation was performed for each sample group, drawing values from a left-shifted normal log2 intensity distribution to model low abundance proteins (mean = 12.1, sd = 1.5). For each protein, a mean intensity profile across all samples was calculated from peptide ion intensities. For each peptide ion, a Pearson correlation coefficient was calculated between the mean protein intensity profile and the individual peptide ion intensity profile. Peptide ions with a correlation coefficient >0.4 were then summed to calculate the label-free intensity of the parent protein. One peptide was required for protein quantification and one phosphopeptide for phosphosite quantification. Protein intensities were log2 transformed and proximal proteins were determined with the limma package^39^ using options trend = TRUE and robust = TRUE for the eBayes function. Protein intensities in the KKT2, KKT3 and CLK2 samples were compared to those in the BDF5 control sample to determine proximal proteins. Multiple testing correction was carried out according to Benjamini & Hochberg, the false discovery rate for identified proximals was 1%.

For miniTurbo phosphosite proximal data, label-free intensities were exported from Progenesis LFQ and phosphopeptides with ≥2 missing values across the 5 biological replicates were removed. Missing values were imputed by drawing values from a left-shifted normal log2 intensity distribution to model low abundance phosphopeptides (mean = 4, sd = 1.2). Phosphosites were aggregated by summing intensities which were then log2 transformed. Proximal phosphosites were determined with the limma package^39^ using options trend = TRUE and robust = TRUE for the eBayes function. Multiple testing correction was carried out according to Benjamini & Hochberg, false discovery rate for proximal phosphosites was 5%.

### KKT sequence alignment

Protein sequences were collected from TriTrypDB either by using the *Leishmania mexicana* gene ID and finding orthologous sequences as listed by TriTrypDB, or the *Trypanosoma brucei* gene ID and finding orthologous sequences as listed by TriTrypDB. Sequences were collected from *T. brucei*, *T. cruzi, T. vivax, L. mexicana, L. amazonensis, L. braziliensis, L. major, L. donovani, L. infantum* and *L. tarentolae* where available. If multiple gene IDs were available for one organism, sequences that were listed on TriTrypDB as being syntenic and from reference strains were chosen preferentially. The entire protein sequence, as listed on TriTrypDB was used for a Clustal Omega (v1.2.4) Multiple Sequence Alignment (MSA) with default settings. All the alignments were generated with the same methodology and settings.

MSAs with a range of *Trypanosomatidae* species were used over pairwise alignments between *T. brucei* and *L. mexicana* to improve the alignment qualities and to gain a better understanding of which phosphosites had evolutionary importance in *Leishmania*, trypanosomes or both.

Alignments are shown with the colours as generated by the Clustal Omega MSA, indicating similar biochemical properties. Residues highlighted in yellow indicate a phosphosite, as identified by our data. Residues highlighted in cyan indicate a phosphosite identified from other data in the literature. Annotations of the sites are listed in boxes below the relevant line of the alignment. Where multiple sites are present on one line, annotations are listed in order from left to right.

### Statistics and reproducibility

Statistical analysis of mass spectrometry data was performed in R 3.6.1 using the limma package. For the BirA* XL-BioID experiments, three replicates were used in each sample group, to achieve 1% FDR amongst proximal proteins, the following *p* value thresholds were used: KKT2 proximals *p* ≤ 0.0009411193, KKT3 proximals *p* ≤ 0.0003713605, CLK2 proximals *p* ≤ 0.001076162. For miniTurbo XL-BioID experiments, five replicates were used in each sample group. To achieve 1% FDR amongst KKT3 proximal proteins, the following *p* value thresholds were used at each timepoint: ES *p* ≤ 0.00280400, S *p* ≤ 0.001802582, G2/M *p* ≤ 0.000724. To achieve 5% FDR amongst KKT3 proximal phosphosites, the following *p* value thresholds were used at each timepoint: ES *p* ≤ 2.613059e-04, S *p* ≤ 0.005294131, G2/M *p* ≤ 0.001837755, G2/M + AB1 *p* ≤ 0.0007286275. All replicates are independent biological replicates.

### Reporting summary

Further information on research design is available in the Nature Portfolio Reporting Summary linked to this article.

## Supplementary information

Supplementary Information

Description of Additional Supplementary Files

Supplementary Data 1

Supplementary Data 2

Supplementary Data 3

Supplementary Data 4

Supplementary Data 5

Supplementary Data 6

Supplementary Data 7

Supplementary Data 8

reporting summary

## Data Availability

Mass spectrometry data sets and proteomic identifications are available to download from MassIVE (MSV000087750), [10.25345/C5G543] and ProteomeXchange (PXD027080). Data for plotting graphs are provided in Supplementary Data 8. Uncropped blots are provided in Supplementary Figs. 17, 18.

## Code availability

Data analysis code has been deposited in Zenodo, 10.5281/zenodo.7316445.
