# Supplementary material for: CLK1/CLK2-driven signalling at the Leishmania kinetochore is captured by spatially referenced proximity phosphoproteomics
Source: Commun Biol. 2022 Nov 28;5:1305. doi: 10.1038/s42003-022-04280-1 (PMC9701682; doi:10.1038/s42003-022-04280-1)
Supplement: Supplementary file 1 — Supplementary Information [file 42003_2022_4280_MOESM1_ESM.pdf]

Supplementary Information

CLK1/CLK2-driven signalling at the *Leishmania* kinetochore is captured by spatially referenced proximity phosphoproteomics

Vincent Geoghegan[1,+], Juliana B T Carnielli[1,+], Nathaniel G. Jones[1], Manuel Saldivia[2,], Sergios Antoniou[1], Charlotte Hughes[1], Rachel Neish[1], Adam Dowle[3], Jeremy C. Mottram[1*]

[1] York Biomedical Research Institute and Department of Biology, University of York, Wentworth Way, Heslington, York YO10 5DD, UK

[2] Novartis Institute for Tropical Diseases, Emeryville, CA, USA

[3] Bioscience Technology Facility, Department of Biology, University of York, York, YO10 5DD, UK

[+] These authors contributed equally

*Corresponding author: jeremy.mottram@york.ac.uk

Supplementary Figures

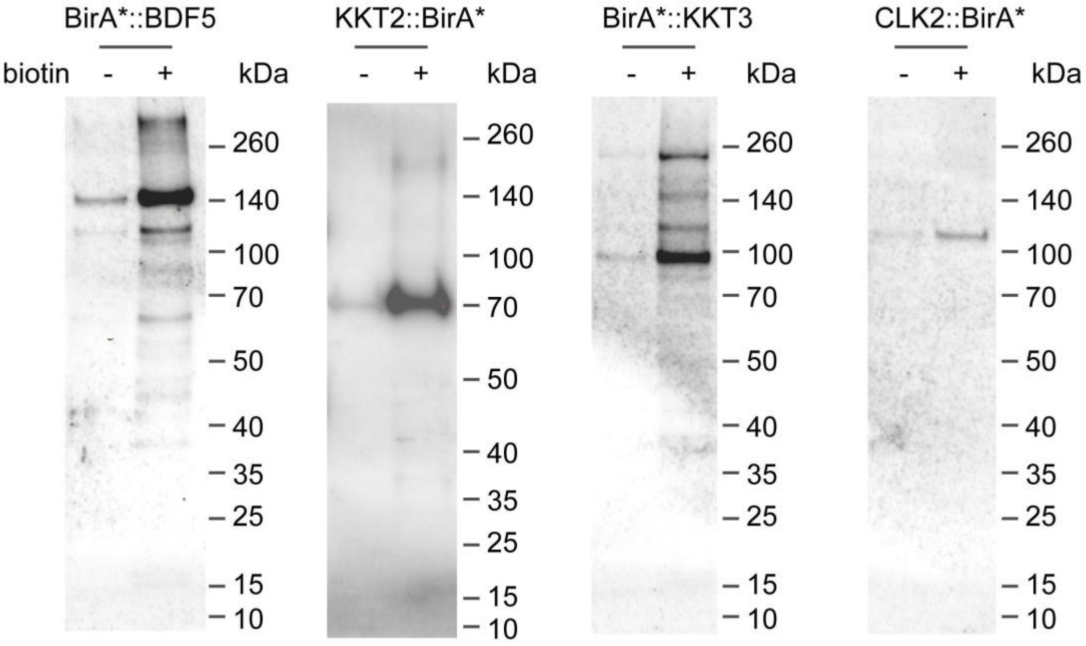

Supplementary Figure 1. Biotinylation of BirA* endogenously tagged proteins used for XL-BioID analysis of the *Leishmania* kinetochore. Parasites were cultured with DMSO (-) or 150μM biotin (+) for 18hrs to induce biotinylation, then processed according to the XL-BioID workflow to enrich biotinylated material. Enrichment of the biotinylated bait protein was assessed by western blotting with anti-myc antibody. Predicted molecular weights: BirA*::BDF5 110kDa, KKT2::BirA* 180kDa, BirA*::KKT3 182kDa, CLK2::BirA* 88kDa.

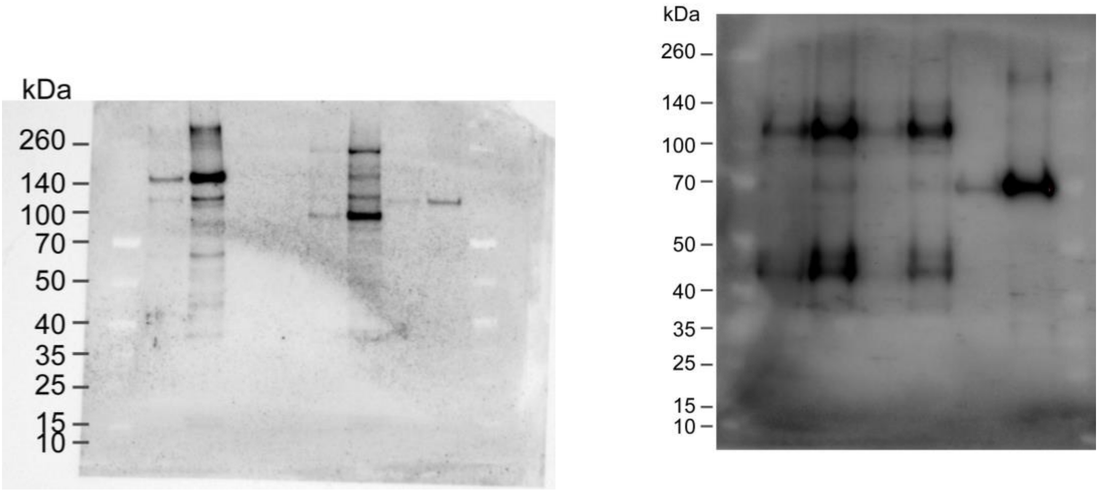

Supplementary Figure 2. Uncropped western blots relating to Supplementary Figure 1. Spectra broad range protein ladder (Thermo Scientific) was used as a marker.

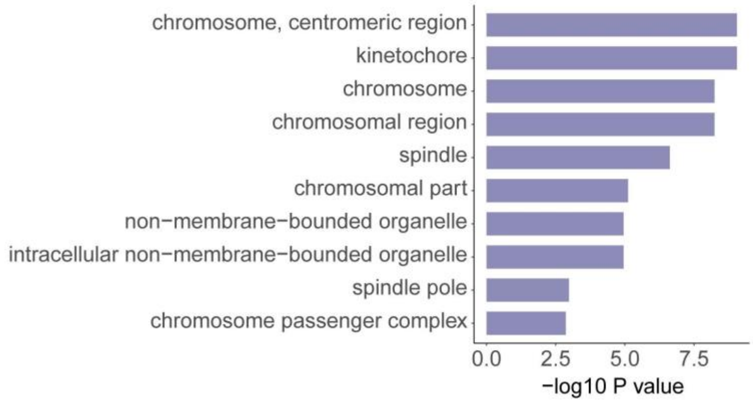

Supplementary Figure 3. Gene ontology analysis of KKT2, KKT3, CLK2 proximal proteins. Top 10 significantly enriched cellular component gene ontology terms.

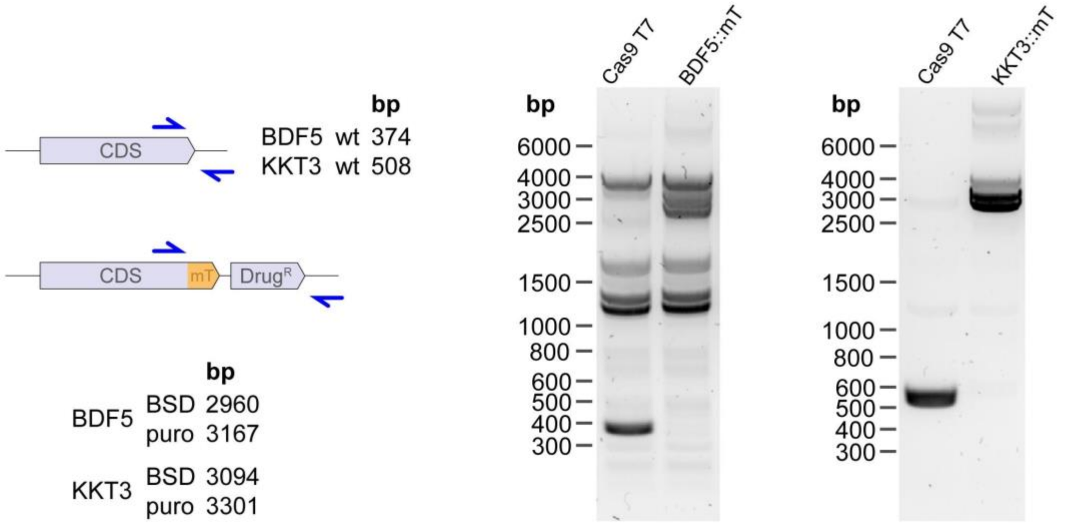

Supplementary Figure 4. Double allele endogenous miniTurbo (mT) tagging of BDF5 and KKT3. Diagnostic PCR was performed on genomic DNA using indicated primers (blue arrows).

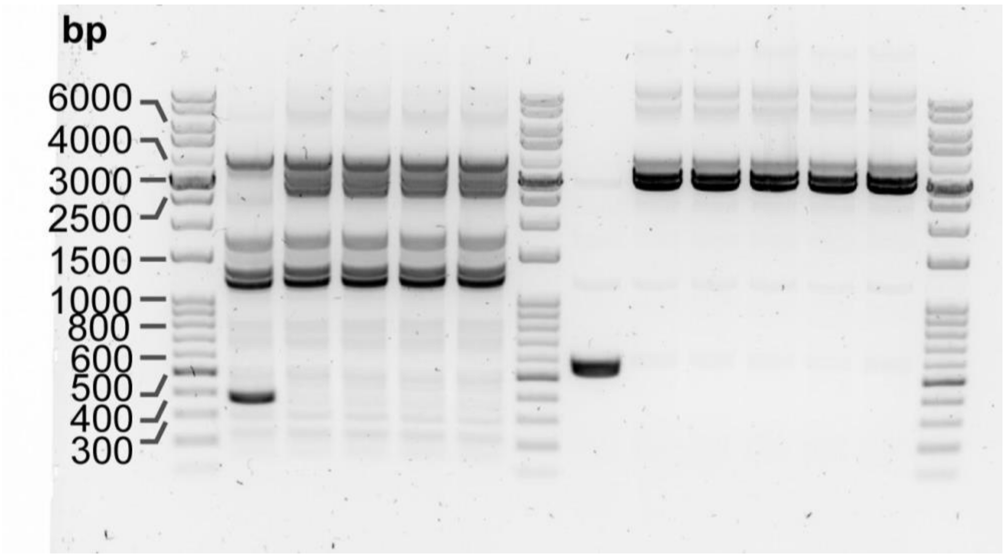

Supplementary Figure 5. Uncropped source image for Supplementary Figure 4.

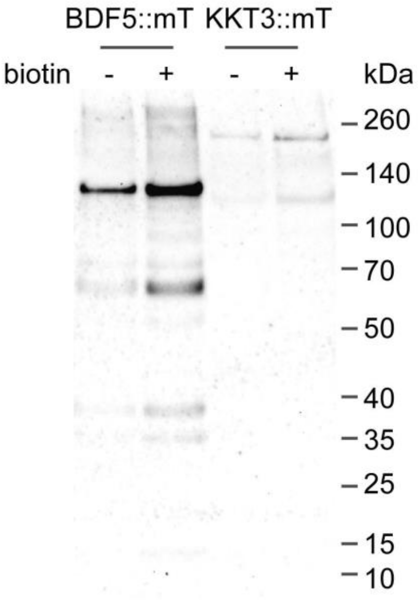

Supplementary Figure 6. Biotinylation of mT tagged proteins. Parasites were cultured with DMSO (-) or 500μM biotin (+) for 30 mins to induce biotinylation, then processed according to the XL-BioID workflow to enrich biotinylated material. Enrichment of the biotinylated bait protein was assessed by western blotting with anti-myc antibody. Predicted molecular weights: BDF5::mT 103kDa, KKT3::mT 175kDa.

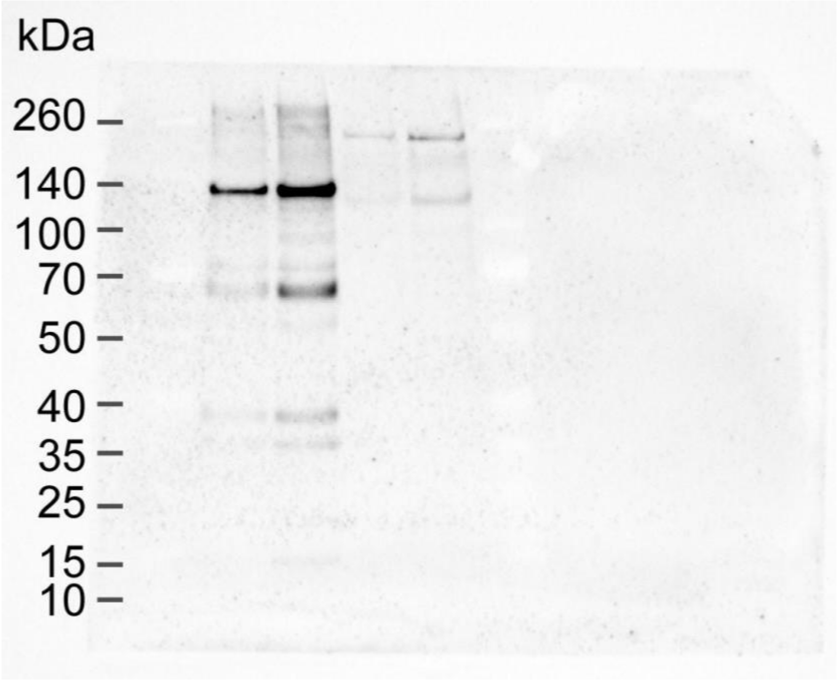

Supplementary Figure 7. Uncropped source image for Supplementary Figure 6.

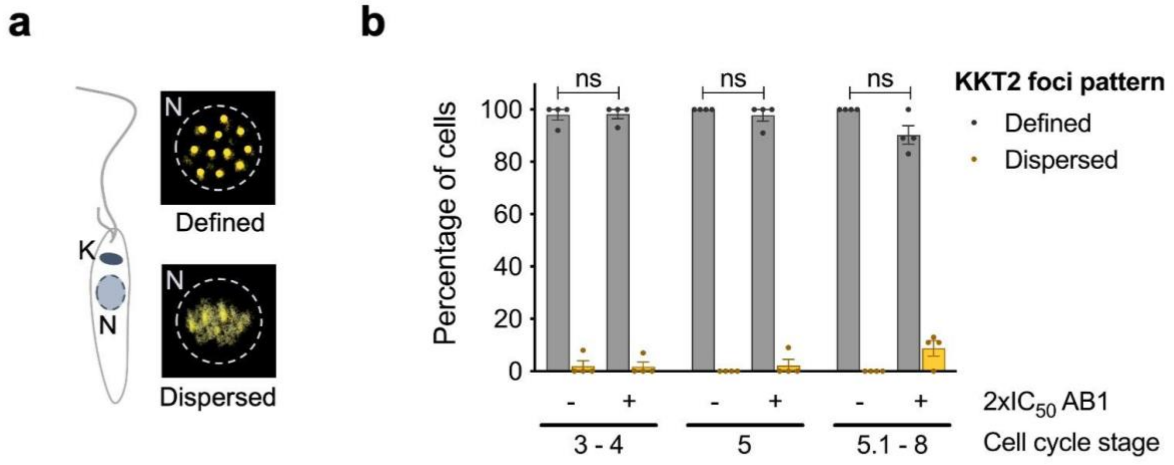

Supplementary Figure 8. CLK1/CLK2 inhibition does not disrupt the foci pattern of the canonical centromere kinetochore protein KKT2. Promastigote-form parasites endogenously expressing KKT2 C-terminally tagged with mNeonGreen (KKT2::mNG) in both alleles were or were not incubated for 6 hrs with 2x IC_{50} AB1 and then processed for immunofluorescence: KMX-1 antibody was used to recognize the mitotic spindle (cells in mitosis); and cells were counterstained with DAPI to visualise DNA. **a,** Scheme of the KKT2 foci pattern assessment by immunofluorescence. **b,** Percentage of cells in stages 3 – 8, according to main figure 3.d, showing a defined kinetochore localization before and after AB1 treatment (n. ≥40 cells in each stage). Error bars, standard errors of the means (SEM); ns, not significant (Mann-Whitney U test).

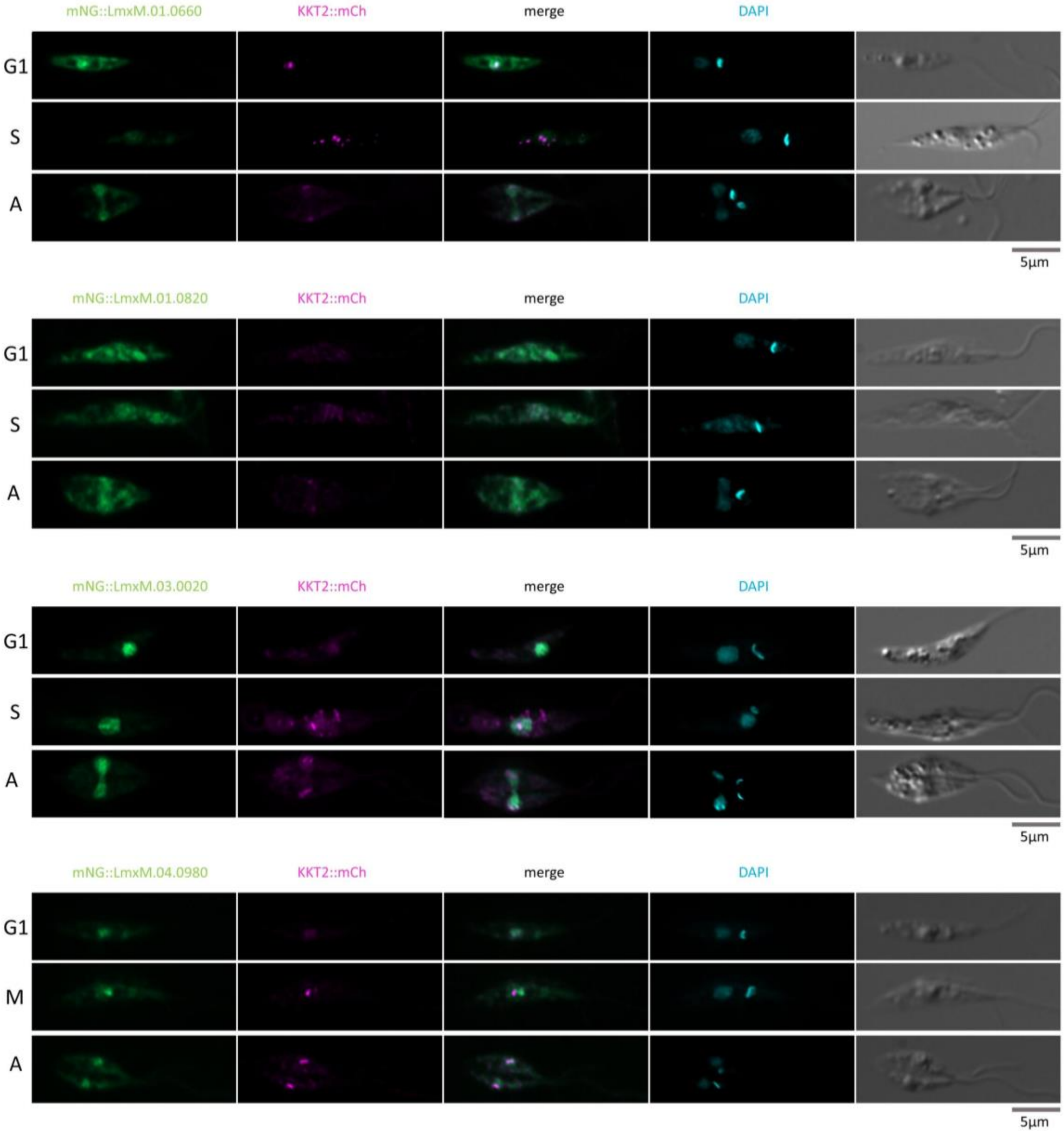

Supplementary Figure 9. Fluorescent co-localisation microscopy screening of kinetochore proximal proteins identified by XL-BioID. LmxM.01.0660, LmxM.01.0820, LmxM.03.0020, LmxM.04.0980 were endogenously tagged with mNeonGreen at the N-terminus, in parasites expressing KKT2 endogenously tagged at the C-terminus with mCherry. Asynchronous, log stage parasites were imaged and G1, S, M (metaphase), A (anaphase) parasites were identified based on KKT2 localisation and DAPI staining of the kinetoplast.

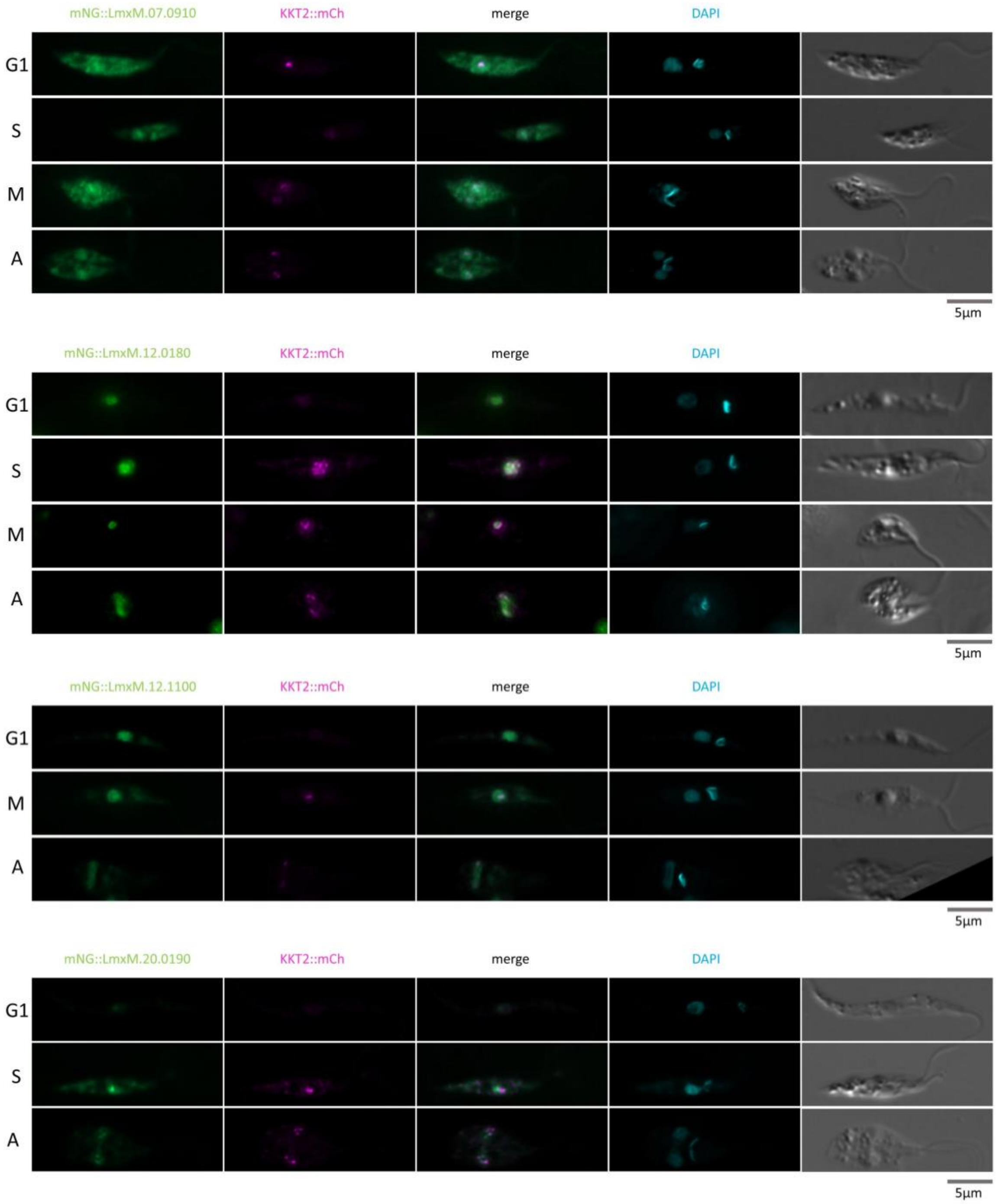

Supplementary Figure 10. Fluorescent co-localisation microscopy screening of kinetochore proximal proteins identified by XL-BioID. LmxM.07.0910, LmxM.12.0180, LmxM.12.1100, LmxM.20.0190 were endogenously tagged with mNeonGreen at the N-terminus, in parasites expressing KKT2 endogenously tagged at the C-terminus with mCherry. Asynchronous, log stage parasites were imaged and G1, S, M (metaphase), A (anaphase) parasites were identified based on KKT2 localisation and DAPI staining of the kinetoplast.

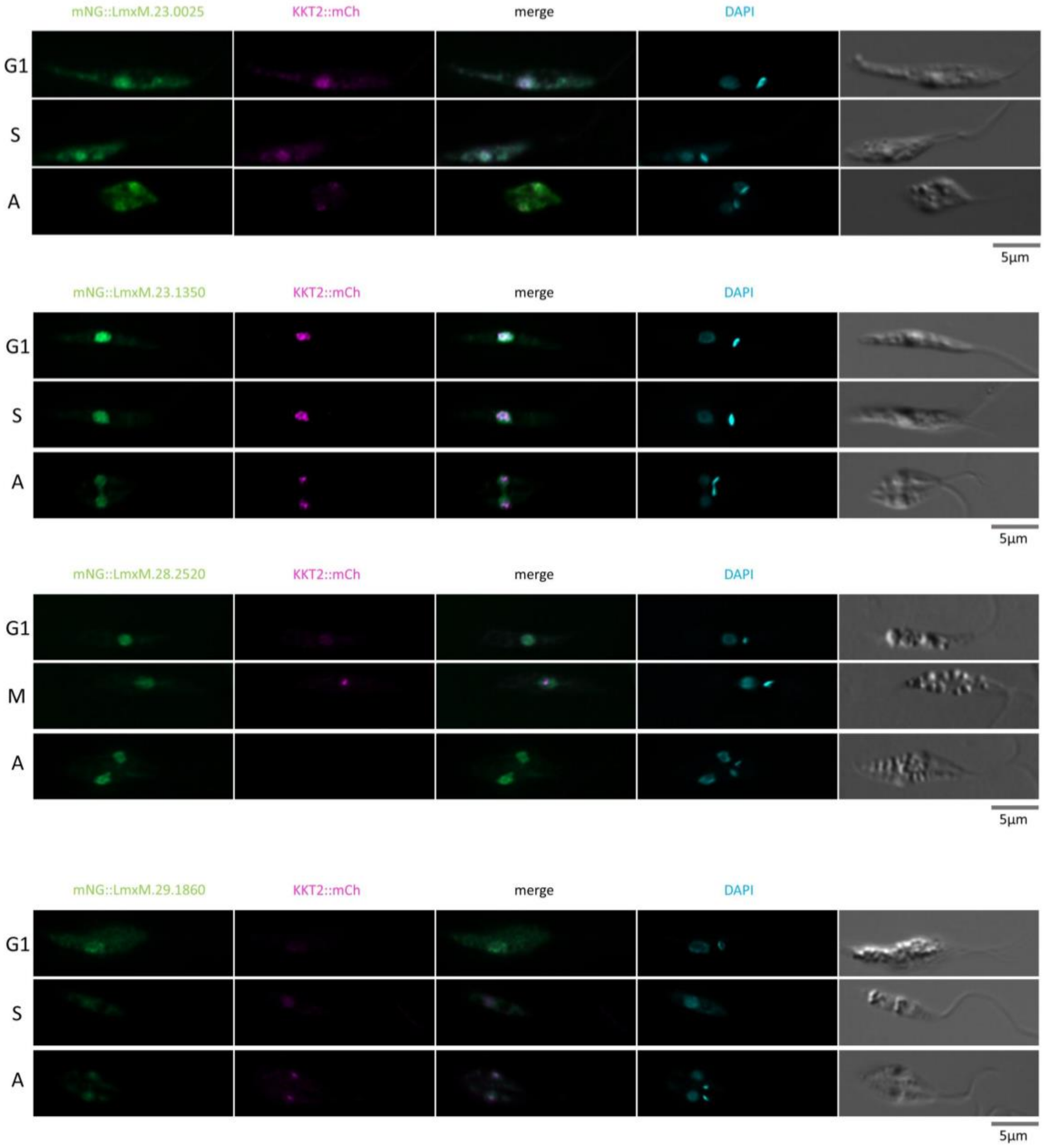

Supplementary Figure 11. Fluorescent co-localisation microscopy screening of kinetochore proximal proteins identified by XL-BioID. LmxM.23.0025, LmxM.23.1350, LmxM.28.2520, LmxM.29.1860 were endogenously tagged with mNeonGreen at the N-terminus, in parasites expressing KKT2 endogenously tagged at the C-terminus with mCherry. Asynchronous, log stage parasites were imaged and G1, S, M (metaphase), A (anaphase) parasites were identified based on KKT2 localisation and DAPI staining of the kinetoplast.

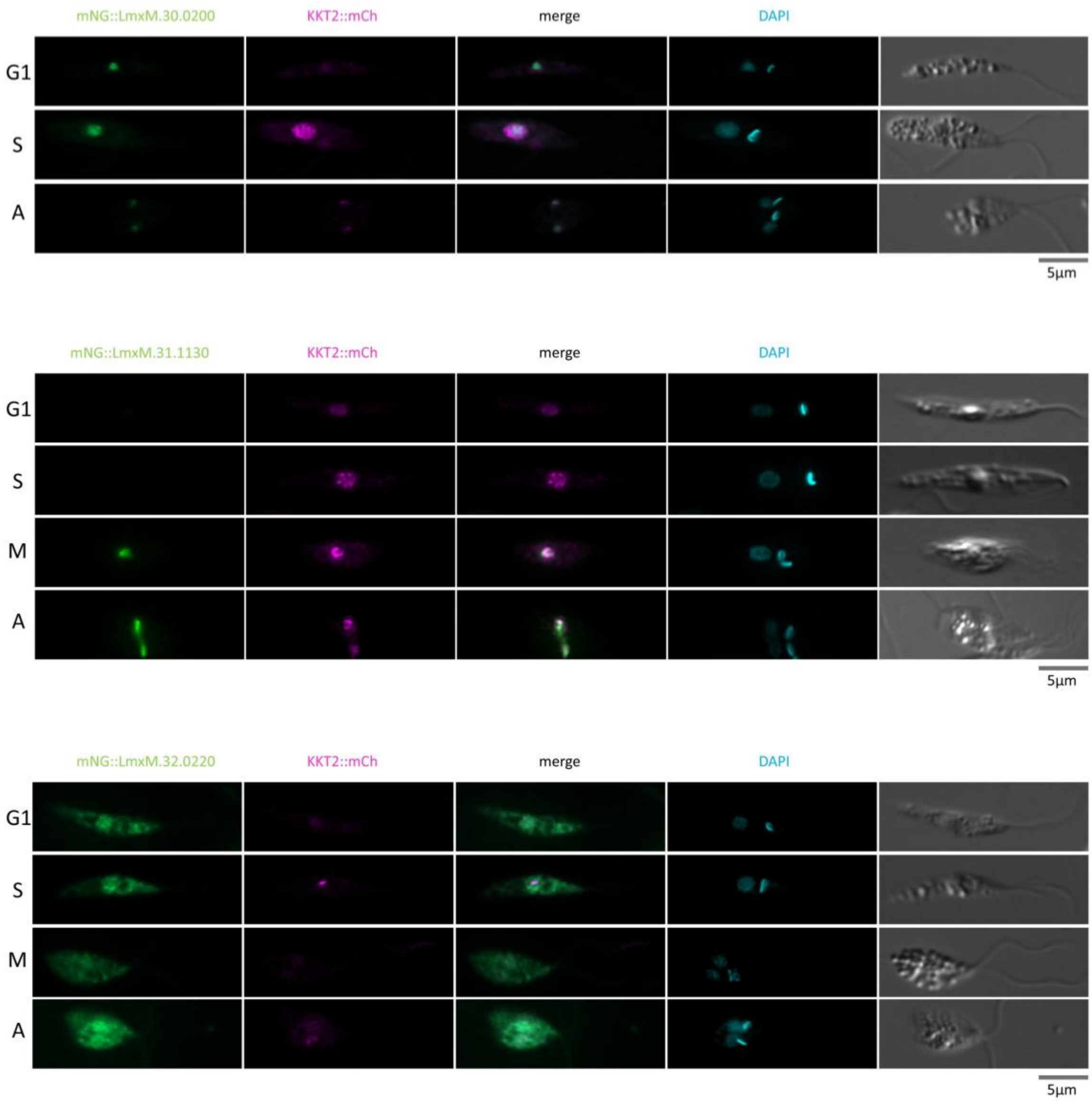

Supplementary Figure 12. Fluorescent co-localisation microscopy screening of kinetochore proximal proteins identified by XL-BioID. LmxM.30.0200, LmxM.31.1130, LmxM.32.0220 were endogenously tagged with mNeonGreen at the N-terminus, in parasites expressing KKT2 endogenously tagged at the C-terminus with mCherry. Asynchronous, log stage parasites were imaged and G1, S, M (metaphase), A (anaphase) parasites were identified based on KKT2 localisation and DAPI staining of the kinetoplast.

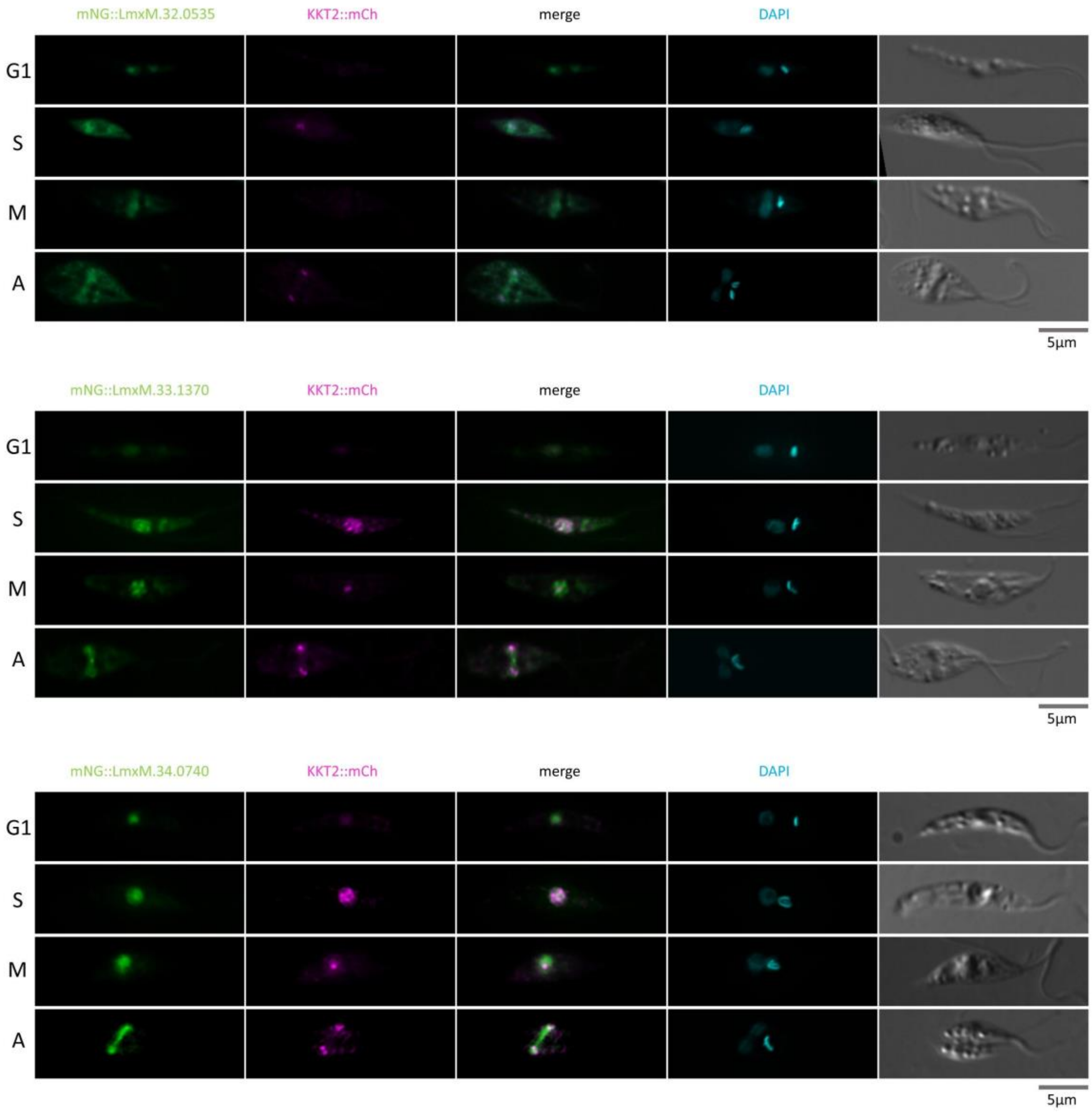

Supplementary Figure 13. Fluorescent co-localisation microscopy screening of kinetochore proximal proteins identified by XL-BioID. LmxM.32.0535, LmxM.33.1370, LmxM.34.0740 were endogenously tagged with mNeonGreen at the N-terminus, in parasites expressing KKT2 endogenously tagged at the C-terminus with mCherry. Asynchronous, log stage parasites were imaged and G1, S, M (metaphase), A (anaphase) parasites were identified based on KKT2 localisation and DAPI staining of the kinetoplast.

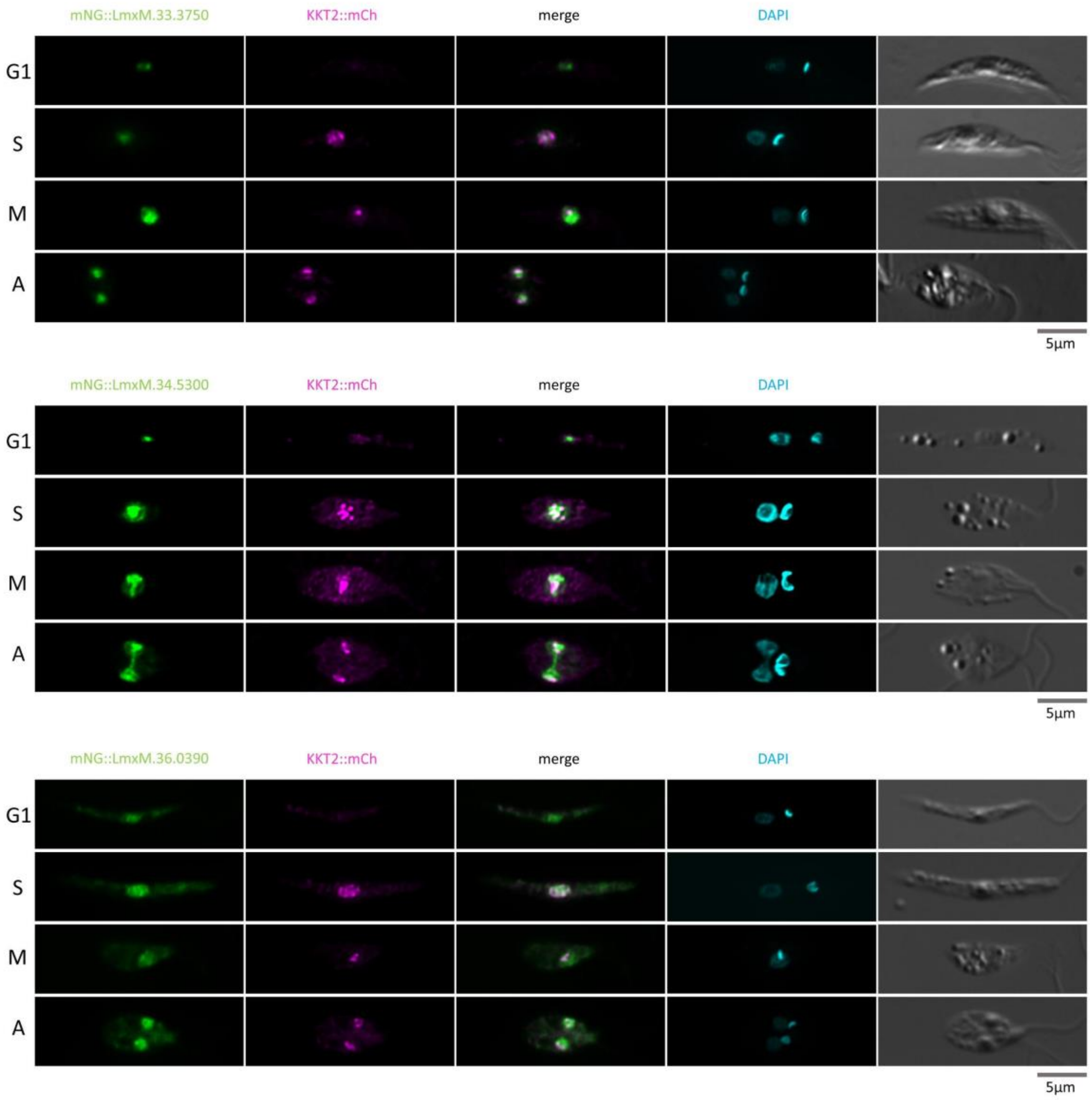

Supplementary Figure 14. Fluorescent co-localisation microscopy screening of kinetochore proximal proteins identified by XL-BioID. LmxM.32.0535, LmxM.33.1370, LmxM.34.0740 were endogenously tagged with mNeonGreen at the N-terminus, in parasites expressing KKT2 endogenously tagged at the C-terminus with mCherry. Asynchronous, log stage parasites were imaged and G1, S, M (metaphase), A (anaphase) parasites were identified based on KKT2 localisation and DAPI staining of the kinetoplast.

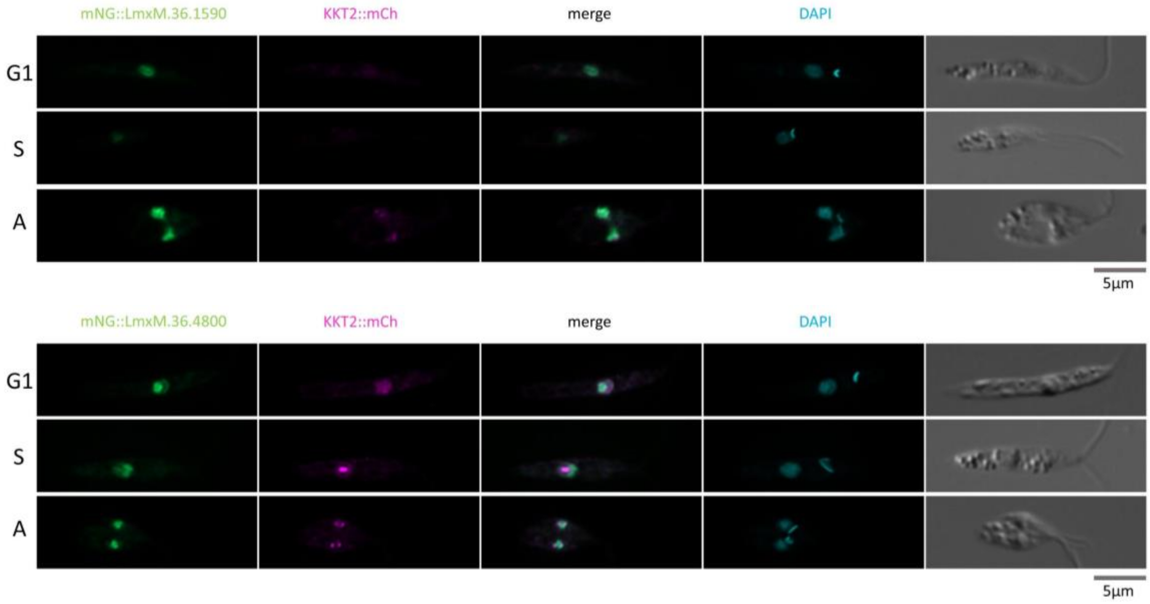

Supplementary Figure 15. Fluorescent co-localisation microscopy screening of kinetochore proximal proteins identified by XL-BioID. LmxM.36.1590, LmxM.36.4800 were endogenously tagged with mNeonGreen at the N-terminus, in parasites expressing KKT2 endogenously tagged at the C-terminus with mCherry. Asynchronous, log stage parasites were imaged and G1, S, M (metaphase), A (anaphase) parasites were identified based on KKT2 localisation and DAPI staining of the kinetoplast.

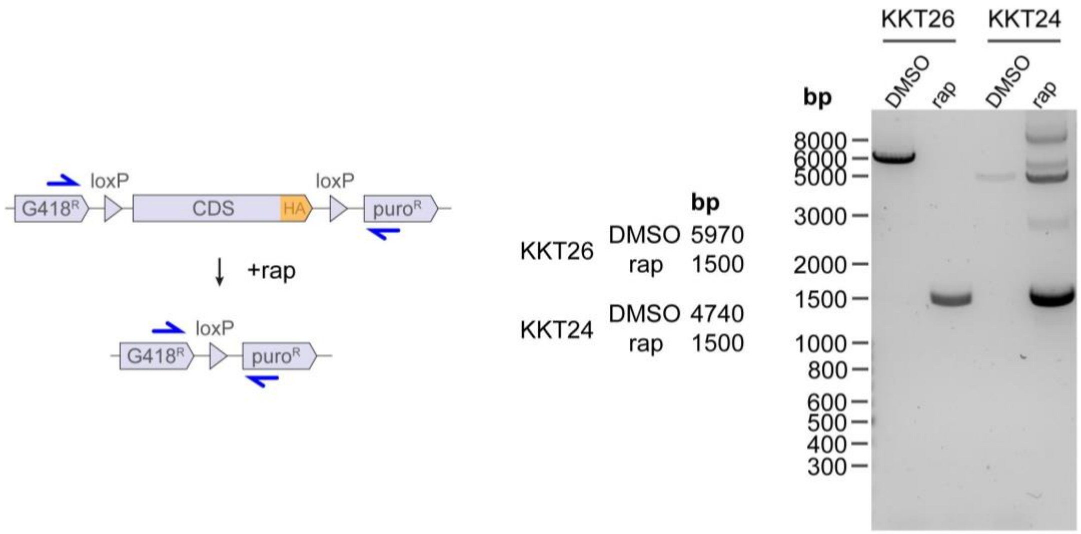

Supplementary Figure 16. Rapamycin induced excision of KKT24 or KKT26. Parasites containing loxP flanked KKT24 or KKT26 were incubated with DMSO or 300nM rapamycin for 48hrs to induce gene excision. Diagnostic PCR was performed on genomic DNA using indicated primers (blue arrows).

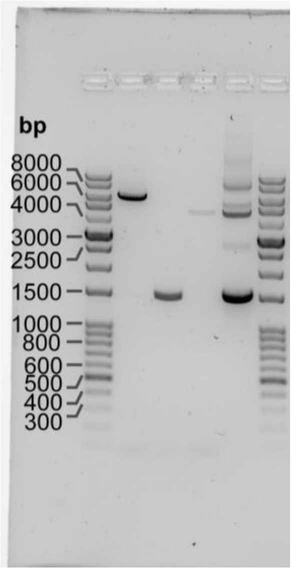

Supplementary Figure 17. Uncropped source image for Supplementary Figure 9.

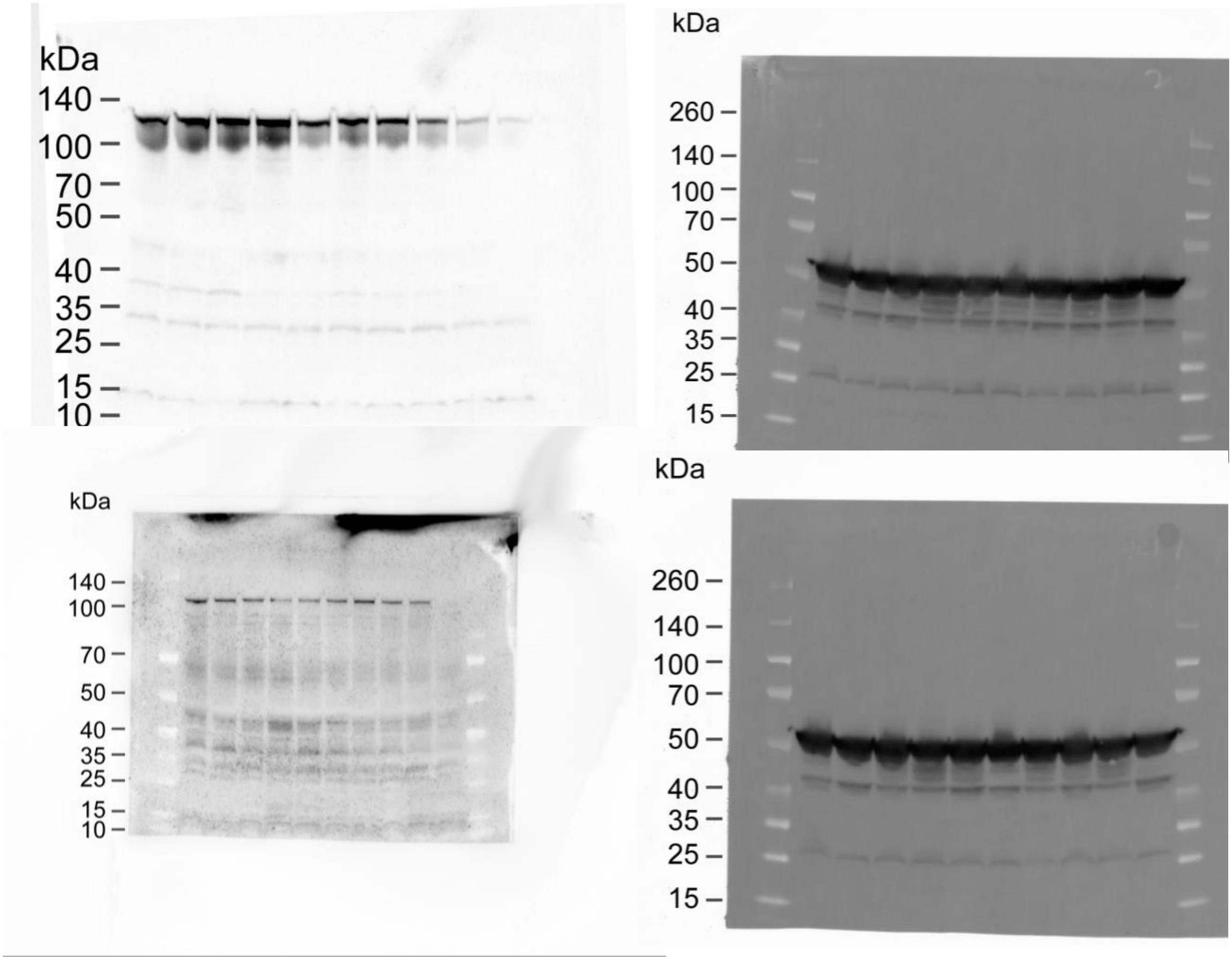

Supplementary Figure 18. Uncropped source images for Figure 6c. Spectra broad range protein ladder (Thermo Scientific) was used as a marker. Immunoblot with anti-HA antibody (Invitrogen 2-2.2.14) and Anti-EF1α Antibody (Millipore clone CBP-KK1).
