# Supplementary material for: CLK1/CLK2-driven signalling at the Leishmania kinetochore is captured by spatially referenced proximity phosphoproteomics
Source: Commun Biol. 2022 Nov 28;5:1305. doi: 10.1038/s42003-022-04280-1 (PMC9701682; doi:10.1038/s42003-022-04280-1)
Supplement: Supplementary file 2 — Description of Additional Supplementary Files [file 42003_2022_4280_MOESM2_ESM.docx]

File name: Supplementary Data 1

Description: BioID vs XL-BioID label free protein intensities

File name: Supplementary Data 2

Description: Proteins classed as proximal to KKT2, KKT3 or CLK2

File name: Supplementary Data 3

Description: All quantified proteins in KKT2, KKT3 and CLK2 proximity biotinylation experiments, with limma results

File name: Supplementary Data 4

Description: KKT3 proximity biotinylation label free protein intensities and limma results

File name: Supplementary Data 5

Description: KKT3 proximity biotinylation label free phosphosite intensities and limma results

File name: Supplementary Data 6

Description: Sequence alignment of KKTs containing phosphosites detected in this work

File name: Supplementary Data 7

Description: Oligonucleotides used

File name: Supplementary Data 8

Description: Data underlying graphs
