# Supplementary material for: CLK1/CLK2-driven signalling at the Leishmania kinetochore is captured by spatially referenced proximity phosphoproteomics
Source: Commun Biol. 2022 Nov 28;5:1305. doi: 10.1038/s42003-022-04280-1 (PMC9701682; doi:10.1038/s42003-022-04280-1)
Supplement: Supplementary file 8 — Supplementary Data 6 [file 42003_2022_4280_MOESM8_ESM.docx]

# **KKT1**

Tb927.10.6330 --MDSFASY--NTRNQVRRRAASVTARSSLSSGADPSLARSRRQM------SISQQPRRL        50
TcCLB.507641.190 --MDLFSATNRSA--------RSQ-QSNSTANGHHSS-------VSSAASRSAAPAQSRR        42
LbrM.35.2090 MVLNLFSGAALNGHGNTHRRERAASSRSSTGTGRRLQQQRRQASSSATGDASVQAEGAGQ        60
LtaP36.1880 MVLNLFSGAALNGHGRTHRRGRASSSLSSTGTGRRPQQQRHQGNRGVTATASMQTDVAEQ        60
LmjF.36.1900 MVLNLFSGAALNGHGSTHRRGRASSSLSSTGTGCRPQQQRYQASRSATDGASMQADGAEQ        60
LmxM.36.1900 MVLNLFSGAALNGHGSTHRRGRASSSLNSTDTGRRPQQQRRQASRSTTYGASMQTDGAEQ        60
LdBPK.36.2.001980 MVLNLFSGAALNGHGSTNRRGRASSSLSSTGTGRRPQQQRRQASRSATDGASMQTDGAEQ        60
LINF_360025400 MVLNLFSGAALNGHGSTNRRGRASSSLSSTGTGRRPQQQRRQASRSATDGASMQTDGAEQ        60
 :: *:. . : .* .* . *

Tb927.10.6330 TVRDLWERAPEEDRLLFTQCVRRVQCRLKESLSSMNFLRELASYYTRTAPLINGFPFCVS        110
TcCLB.507641.190 SGRELLETTSEEDKSLFVRCVEQVQRQLKPNINSPSTLHTLASYYTREEPYVEGRPFCVS        102
LbrM.35.2090 SG-SGVHSDIADDRVLFDNCVAQVQRHLKTHADSPSTLHTLASYYTKTEPFIEGRPFCVA        119
LtaP36.1880 SG-TEVRTEVAEDRVLFNNCVAQVQRHLKAHADSPSTLHTLASYYTKTEPVIEDRPFCVA        119
LmjF.36.1900 SG-SGVRAEAAEDRVLFNNCVAQVQRHLKTHADSPSTLHTLASYYTKTEPFIEGRPFCVT        119
LmxM.36.1900 SG-SGLRAEAAEDRVLFNNCVAQVQRHLKTHADSPSTLHTLASYYTKTEPFIEGRPFCVT        119
LdBPK.36.2.001980 SG-SGVRAEAAEDRVLFNNCVAQVQRHLKTHADSPSTLHTLASYYTKTEPFIEGRPFCVT        119
LINF_360025400 SG-SGVRAEAAEDRVLFNNCVAQVQRHLKAHADSPSTLHTLASYYTKTEPFIEGRPFCVT        119
 : . :*: ** .** :** :** .* . *: ******: * ::. ****:

Tb927.10.6330 LSYATFLFHMQMPHVCIEDIQRYAQLIATAIDIIPDTQRSEHPFVRDVIRGDVFGLSSPS        170
TcCLB.507641.190 LSYATFLFHMQMARVSESDVQLYAHLINAILAQISDDARMTHPFVLRVLRDAVFGLPSPT        162
LbrM.35.2090 LSYATFLFHMQMARISVADVELYVQLLTSILAQLTEDDQLHHPFVQQVLRDHVFGLPSPT        179
LtaP36.1880 LSYATFLFHMQMARISLSDVELYVQLLTSVLSQINEDDQLHHPFVQQVLRDHVFGLPSPT        179
LmjF.36.1900 LSYATFLFHMQMARISVADVELYVQLVISALSQITEDDQLHHPFVQQVLRDHVFGLPSPT        179
LmxM.36.1900 LSYATFLFHMQMARISVTDVELYVQLLTSILSQITEDDQLHHPFVQQVLRDHVFGLPSPT        179
LdBPK.36.2.001980 LSYATFLFHMQMARISVADVELYVQLVTSVLSQITEDDQLHHPFVQQVLRDHVFGLPSPT        179
LINF_360025400 LSYATFLFHMQMARISVADVELYVQLVTSVLSQITEDDQLHHPFVQQVLRDHVFGLPSPT        179
 ************ ::. *:: *.:*: : : : : : **** *:*. **** **:

Tb927.10.6330 LEGPAHYVVLVPSLQYRAFSLLAVTLVEHGVVPVDIINQWQRKLRELCRATSSLVSNRAL        230
TcCLB.507641.190 CVGGAHCVALVPPLQYRAFACLATSLVDLRIVPVDILYQWQDKLEALCDAQSPLVANRAL        222
LbrM.35.2090 CRGAAHSVVLLSPQQYRAFATMTTALISLAVVPLSILYQFQDRLETYCECASPLVANRAL        239
LtaP36.1880 CRGAAHSVVLLPPQQYRAFATMATALISLAVVPLSVVYQFHDRLETYCECTSPLVANRAL        239
LmjF.36.1900 CRGAAHSVVLLSPQQYRAFATMTTALISLAVVPLSIVYQFHDRLETYCECASPLVANRAL        239
LmxM.36.1900 CRGAAHSVVLLSPQQYRAFATMTTALISLAVVPLSIVYQFHDRLETYCECASPLVANRAL        239
LdBPK.36.2.001980 CRGAAHSVVLLSPQQYRAFATMTTALISLAVVPLSIVYQFHDRLETYCECASPLVANRAL        239
LINF_360025400 CRGAAHSVVLLSPQQYRAFATMTTALISLAVVPLSIVYQFHDRLETYCECASPLVANRAL        239
 * ** *.*: *****: ::.:*:. :**:.:: *:: :*. * . * **:****

Tb927.10.6330 TLILKTAEEVDREEQVEVLMNVIRSSPRRMHVEQIVACYERLKRAAPCISTGPMYGRSLS        290
TcCLB.507641.190 TLIVHTIGTIRMDEQIAALQYVLKTRPRKMNVDFLLACYERLKRAVPEPVRGPMYGRAIS        282
LbrM.35.2090 ALLVQTVGEVRMDEQVTALQYVLKTKPVKMNVDFLLACYERLKRAVMDPAHGPTFGRALS        299
LtaP36.1880 ALLVQTVGEVRMDEQVTALQYVLKTKPVKMNVDFLLACYERLKRAVVDPAHGPTFGRALS        299
LmjF.36.1900 ALLVQTVGEVRMDEQVTALQYILKTKPVKMNVDFLLACYERLKRAVIDPAHGPSFGRALS        299
LmxM.36.1900 ALLVQTVGEVRMDEQVTALQYVLKTKPVKMNVDFLLACYERLKRAVMDPAHGPSFGRALS        299
LdBPK.36.2.001980 ALLVQTVGEVRMDEQVTALQYVLKTKPVKMNVDFLLACYERLKRAVIDPAHGPSFGRALS        299
LINF_360025400 ALLVQTVGEVRMDEQVTALQYVLKTKPIKMNVDFLLACYERLKRAVIDPAHGPSFGRALS        299
 :*:::* : :**: .* :::: * :*:*: ::*********. ** :**::*

Tb927.10.6330 IRLTSVFLTLRSPIRKEFVESFLYPSLCSEDVKDTLNQPAVRMHLYRKLLRLCTPGMGTS        350
TcCLB.507641.190 IHCSELFLRFRSPIRREYVERFLYPSLSGADMQSLVQIPATRQHLCGELLRQCTPGMSPS        342
LbrM.35.2090 IHCSELFLRFRSPVRRDYVERFLYPSLCHSDMAGFLEIPATRKHLLHELLSQCTPGMGTM        359
LtaP36.1880 IHCSELFLRFRSPVRRDYVERFLYPSLCHSDMASFLEIPATRKHLLRELLSQCTPGMGTM        359
LmjF.36.1900 IHCSELFLRFRSPVRRDYVERFLYPSLCHSDMASFLEIPATRKHLLRELLSKCTPGMGTM        359
LmxM.36.1900 IHCSELFLRFRSPVRRDYVERFLYPSLCHSDMASFLEIPATRKHLLRELLSQCTPGMGTM        359
LdBPK.36.2.001980 IHCSELFLRFRSPVRRDYVERFLYPSLCHSDMAGFLEIPATRKHLLRELLSQCTPGMGTM        359
LINF_360025400 IHCSELFLRFRSPVRRDYVERFLYPSLCHSDMAGFLEIPATRKHLLRELLSQCTPGMGTM        359
 *: :.:** :***:*:::** ******. *: . :: **.* ** :** *****.

Tb927.10.6330 NPYYLCICAIIQPFLDDVTEGSLEIVSLVKCLMPHAAYFVATLTLDTRLQAEVLAGIVVS        410
TcCLB.507641.190 NPYYLCLCAVMQWAFEDEIDGAMEMVELINCQMPHAAYFAATLAVDTRMSVAMFAKIIIA        402
LbrM.35.2090 NPFYMCLCAVLQSCFDNETDGALETVALINCHMPHAAYFMSTLAIDSHMSVPMFAKVMIS        419
LtaP36.1880 NPFYMCLCAVLQSRFDNETDGALETVALINCHMPHAAYFMSALAVDSHMSVPMFAKVMIS        419
LmjF.36.1900 NPFYMCLCAVLQSCFDNETDGALETVALIDCHMPHAAYFMSTLAVDSHMSVPMFAKVMIS        419
LmxM.36.1900 NPFYMCLCAVLQSCFDNETDGALETVALINCHMPHAAYFMSTLAVDSHMSVPMFAKVMIS        419
LdBPK.36.2.001980 NPFYMCLCAVLQSCFDNETDGALETVALINCHMPHAAYFMSTLAVDSHMSVPMFAKVMIS        419
LINF_360025400 NPFYMCLCAVLQSCFDNETDGALETVALINCHMPHAAYFMSTLAVDSHMSVPMFAKVMIS        419
 **:*:*:**::* ::: :*::* * *:.* ******* ::*::*:::.. ::* ::::

Tb927.10.6330 LVGSCARVMKRVLMNSPVARAV-ESPATVYSILFLLREVVRGCSISSSERATLMLQCIGA        469
TcCLB.507641.190 LVRGAGLAMTGREVSDDVATAL-RNRTNVYSVLFLLREVVRNCSITASRRATDMHKALSI        461
LbrM.35.2090 LARGAGMAMTGRETPDEVAASINENRTSVYNVLFLLREVVRSCSITASRRAIDMLKALRV        479
LtaP36.1880 LARGAGMAMTGRETPDEVAASINENRTSVYNVLFLLREVVRSCSTTASRRATDMLKALRV        479
LmjF.36.1900 LARGAGMAMTGRETPDEVAASINENRTSVYNVLFLLREVVRSCSTTASRRATDMLKALRV        479
LmxM.36.1900 LARGAGMAMTGRDTPDEVAASINENRTSVYNVLFLLREVVRSCSTTASRRATDMLKALRV        479
LdBPK.36.2.001980 LARGAGMAMTGRETPDEVAASINENRTSVYNVLFLLREVVRSCSTTASRRATDMLKALRV        479
LINF_360025400 LARGAGMAMTGRETPDEVAASINENRTSVYNVLFLLREVVRSCSTTASRRATDMLKALRV        479
 *. ... .*. . ** :: .. :.**.:*********.** ::*.** * :.:

Tb927.10.6330 VANPSVMKTLGKVTKEAFEKFLPSGGDTCNSLVGGGAFPYIVDPQLLCAEMDMVLHGRHV        529
TcCLB.507641.190 AVPEKTVEALGKLAMEAFNDCN----------------EATVDPQLICAELAMVLHQLHI        505
LbrM.35.2090 AVAPKTIEALGKLSSEAFEAFS----------------DITLDPQLLCAELAMVLHQDHI        523
LtaP36.1880 AVAPKTIEALGKLSSEAFETVS----------------DITLDPQLLCAELAMVLHQDHI        523
LmjF.36.1900 AVAPKTIEALGKLSSEAFEAVS----------------DITLDPQLLCAELAMVLHQDHI        523
LmxM.36.1900 AVAPKTIEALGKLSSEAFEAVS----------------DITLDPQLLCAELAMVLHQDHI        523
LdBPK.36.2.001980 AVAPKTIEALGKLSSEAFEAVS----------------DITLDPQLLCAELAMVLHQDHI        523
LINF_360025400 AVAPKTIEALGKLSSEAFEAVS----------------DITLDPQLLCAELAMVLHQDHI        523
 .. ..:::***:: ***: :****:***: **** *:

Tb927.10.6330 CEAIDIILQHNWNIGSICALCGETRGQSPLCQASGAAHYVGTASLGRVLVTITECAGSEV        589
TcCLB.507641.190 DEAMDAAVAHLRDVTALCIFCGLGRGASTLCEVNGTMHMTGHASVGRVLTTLAECAGIPS        565
LbrM.35.2090 AEAMDSAVEYFRDATSKCPHCAAARSSSLLCPVNGTVHVAGQSSASRVLSTLSECAGAKA        583
LtaP36.1880 AEAMDSAVEYFRDVRSKCPYCAAARSSSLLCPVNGTVHVAGQSSVIRVLSTLSECAGTKA        583
LmjF.36.1900 AEAMDSAVEYFRDVRSRCPCCAAARSSSLLCPVNGTVHVAGQSSVSRVLSTLSECAGAKA        583
LmxM.36.1900 AEAMDSAVEYFRDVRSKCPYCAAARSSSLLCPVNGTVHVAGQSSVSRVLSTLSECAGAKA        583
LdBPK.36.2.001980 AEAMDSAVEYFRDVRSRCPYCAAARSSSLLCPVNSTVHVAGQASVSRVLSTLSECAGAKA        583
LINF_360025400 AEAMDSAVEYFRDVRSRCPYCAAARSSSLLCPVNSTVHVAGQASVSRVLSTLSECAGAKA        583
 **:* : : : : * *. *. * ** ...: * .* :* *** *::****

Tb927.10.6330 VREKLKSLMCDSSRRLDSAVHLLLFHIFAHAGPHRKELYLEIEPYIRSTLVEVLNKS--R        647
TcCLB.507641.190 VEGKLIQLLRDPATQMDSAVHFLLFHILSHAGQHRHTLFQAVEPYIRSTLTTLLSADRAS        625
LbrM.35.2090 VEERLISYLRDPTLQMESSVHFLIYHIIANGGQHRNTLFVAVEPYVRSTLLALVSADRSG        643
LtaP36.1880 VEEKLISYLRDPALQMESAVHYLIYHIIANGGQHRNTLFVAVEPYVRSTLLALVSADRSG        643
LmjF.36.1900 VEEKLISYLRDPALQMESAVHYLIYHIIANGGQHRNTLFVAVEPYVRSTLLALVSADRSG        643
LmxM.36.1900 VEEKLISYLRDPALQMESAVHYLIYHIVANGGQHRNTLFVAVEPYVRSTLLALVSADRSG        643
LdBPK.36.2.001980 VEEKLIGYLRDPALQMESAVHYLIYHIIANGGQHRNTLFVAVEPYVRSTLLALVSADRSG        643
LINF_360025400 VEEKLIGYLRDPALQMESAVHYLIYHIIANGGQHRNTLFVAVEPYVRSTLLALVSADRSG        643
 *. :* : * : :::*:** *::**.::.* **: *: :***:**** ::. .

Tb927.10.6330 AFGVLDSEERASLLTLHAKLVILLGNAVDRSHVDALLQALSCIQIHSNHDALVLWYLANL        707
TcCLB.507641.190 SAGLVPSQQKANVLMLHVKIVILLANSIEPSYVESILRVFCEVRLRNNHDALALWYMANL        685
LbrM.35.2090 TRGLVSSTLKANVLMLHVKLVTLLSSSIDPSYLESILKVFSELRVRNNHDALALWYMGNV        703
LtaP36.1880 VRGLVDSTLKANVLMLHVKLVTLLASSIDPSYLESILKVFSELRVRNNHDALALWYMGNV        703
LmjF.36.1900 VRGLVDSTLKANVLMLHVKLVTLLASSIDPSYLESILKVFSELRVRNNHDALALWYMGNV        703
LmxM.36.1900 VRGLVDSTLKANVLMLHVKLVTLLASSIDPSYLESILKVFSELRLRNNHDALALWYMGNV        703
LdBPK.36.2.001980 VRGLVDSTLKANVLMLHVKLVTLLASSIDPSYLESILKVFSELRVRNNHDALALWYMGNV        703
LINF_360025400 VRGLVDSTLKANVLMLHVKLVTLLASSIDPSYLESILKVFSELRVRNNHDALALWYMGNV        703
 *:: * :*.:* **.*:* **..::: *:::::*:.:. ::::.*****.***:.*:

Tb927.10.6330 LLRRGGSNVDLLPTAPDENNYCVEYPDCAPTCKEAADNAQLLLKIIHRAHYFSPQMRKLV        767
TcCLB.507641.190 LLRQGKGNVELLPTDPAENNYSVNYPSCAPMCNTTADNAQLLLKIIDRSHSFSDEMRKLV        745
LbrM.35.2090 LLRQLRSNLELLPTDPQENNYCVAFPGCAPASATTANNAQLVLKLLHRAHSFSPEMHKLV        763
LtaP36.1880 LLRSCRGNLELLPTDPQENNYCVAFPGCAPASVTTADNAQLVLKLLHRAHSFSPEMHKLV        763
LmjF.36.1900 LLRSCRGNLELLPTDPQENNYCVAFPGCAPASATTADNAQLVLKLLHRAHSFSPEMHKLV        763
LmxM.36.1900 LLRSCRGNLELLPTDPQENNYCVAFPGCAPASATTADNAQLVLKLLHRAHSFSPEMHKLV        763
LdBPK.36.2.001980 LLRSCRGNLELLPTDPQENNYCVAFPGCAPASATTADNAQLVLKLLHRAHSFSPEMNKLV        763
LINF_360025400 LLRSCRGNLELLPTDPQENNYCVAFPGCAPASATTADNAQLVLKLLHRAHSFSPEMNKLV        763
 *** .*::**** * ****.* :*.*** . :*:****:**::.*:* ** :*.***

Tb927.10.6330 GCCVCKLIQDFNMQADNIITALLSPFGSVPVSLNSLVEYALPVGANSTFWSFFLRQMKTS        827
TcCLB.507641.190 GCCVCKLIQDFNVQSPNIIGALLSPFGFVPVGLQPLSEYALPVGASSTFWTFFVHQMKTS        805
LbrM.35.2090 GCCVCKLIQEFNMQAPNICSTLLSPFGFLPVGLESLNTFALPVGAGSTFWSFFLQQMRSS        823
LtaP36.1880 GCCVCKLIQDFNIQAPNICSTLLSPFGFLPVGLESLNAFALPAGAGSTFWSFFLQQMRSS        823
LmjF.36.1900 GCCVCKLIQDFNMQAPNICSTLLSPFGFFPVGLESLNAFALPAGASSTFWSFFLQQMRSA        823
LmxM.36.1900 GCCVCKLIQDFNMQAPNICSTLLSPFGFFPVGLESLNAFALPAGAGSTFWSFFLQQMRSS        823
LdBPK.36.2.001980 GCCVCKLIQDFNMQAPNICSTLLSPFGFFPVGLESLNAFALPAGAGSTFWSFFLQQMRSS        823
LINF_360025400 GCCVCKLIQDFNMQAPNICSTLLSPFGFFPVGLESLNAFALPAGAGSTFWSFFLQQMRSS        823
 *********:**:*: ** :****** .**.*: * :***.**.****:**::**:::

Tb927.10.6330 APARTALLASFVKSVTRRFSVASPVSCMPITGEETTAELFAVMAYETVRRCPPLARVVLH        887
TcCLB.507641.190 APARVAFMTALAKSISQRFRIAAPADAVCIDGTEATGHLFAVMMYEAMKRNPPLTRVVLH        865
LbrM.35.2090 APARTAFMATLAKSLSRRFRIASPIEALAPYGVEPTGHLFVIMVYEAMKRNPPLARVLLY        883
LtaP36.1880 APARTAFMATLAKSLSRRFRIASPMDALAPYGVEPTGHLFVVMVYEAMKRNPPLARVLLY        883
LmjF.36.1900 APARTAFMATLAKSLSRRFRIASPRDALAPHGVEPTGHLFVIMVYEAMKRNPPLARVLLY        883
LmxM.36.1900 APARTAFMATLAKSLSRRFRIASPMDALAPYGVEPTGHLFVIMVYEAMKRNPPLARVLLY        883
LdBPK.36.2.001980 APARTAFMATLAKSLSRRFRIASPRDALAPHGVEPTGHLFVIMVYEAMKRNPPLARVLLY        883
LINF_360025400 APARTAFMATLAKSLSRRFRIASPRDALAPYGVEPTGHLFVIMVYEAMKRNPPLARVLLY        883
 ****.*:::::.**:::** :*:* ..: * * *..**.:* **:::* ***:**:*:

Tb927.10.6330 LLTSWVKQTRSTPGRFVSLVYTSLQMVMVIFRRSVGADVAEVGAETRQDAHHFGEAVSKA        947
TcCLB.507641.190 MISQWVKQQNHPPGRFACLLYICTQLLTVVVQRGTGLDAIELAAETRQDMHQFADAVTKT        925
LbrM.35.2090 MVSHWTKQAGHPPGKFACLVYACVQLITVVVDRAEGAAAAEVEAETPQDRQQFDDAVKKA        943
LtaP36.1880 MVSHWMKQAGHPPGKFACLVYVCVQLITVVVKRAEGPAAAEVEAETPQDRQQFDDAVKKA        943
LmjF.36.1900 MVSHWMKQAGHPPGKLACLVYVCVQLITVVVDRAEGPAAAEVEAETPQDRQQFDDAVKKA        943
LmxM.36.1900 MVSHWMKQAGHPPGKLACLVYVCVQLITVVVDRAEGPAAAEVEAETPQDRQQFDDAVKKA        943
LdBPK.36.2.001980 MVSHWMKQAGHPPGKLACLVYVCVQLITVVVDRAEGPAAAEVEAETPQDRQQFDDAVKKA        943
LINF_360025400 MVSHWMKQAGHPPGKLACLVYVCVQLITVVVDRAEGPAAAEVEAETPQDRQQFDDAVKKA        943
 ::: * ** **::..*:* . *:: *:. *. * . *: *** ** ::* :**.*:

Tb927.10.6330 VQEIKMQVGRINDMAREIRDENVVFFRLLVRLLKRTKFVVKETVGESCDEIADMLGAGDS        1007
TcCLB.507641.190 TTIVRGQLPRLARLGPLVRQENIWFYQVLHRLHHKTRRLVEETTGEKCDEGS--SVFDEG        983
LbrM.35.2090 VRVLKSQQARLDRLEPTARRENVEFFHLLRRLQRRVRRTVAAASGEIVVGDEAAEEYNDY        1003
LtaP36.1880 AQVLKSQQARLDRLAPTARRENVEFFHLLRRLQRRVRRTVAVASGELVVGDEAAEEYSDH        1003
LmjF.36.1900 ARVLKSQQARLDRLAPTARRENVEFFHLLRRLQRRVRRTVAAASGEIVVGDEAAEEDDDH        1003
LmxM.36.1900 ARVLKSQQARLDRLAPTARRENVEFFHLLRRLQRRVRRTVATASGEIVVGDEAAEEYDDH        1003
LdBPK.36.2.001980 ARVLKSQQTRLDKLAPTARRDNIEFFHLLRRLQRRVRCTVAAASGEIVVGDEAAEECDDH        1003
LINF_360025400 ARVLKSQQARLDKLAPTARRDNIEFFHLLRRLQRRVRCTVAAASGEIAVGDEAAEECDDH        1003
 . :: * *: : * :*: *:::* ** ::.: * : ** .:

Tb927.10.6330 LDNEEEEGAGVSVEVCDVAQPDGTPYQGGELFSYSLEELQRPSDDNVFDDLTMSHGD---        1064
TcCLB.507641.190 FNTTMNK--------TAELTG-GASVGTNVQEAYLLDSLHQPDDNTVFEDYMEGDDTMSP        1034
LbrM.35.2090 -DGAVD----------GSLDARRARQDGTTDAVYAMQELQNAADNSVFDDYADDGNHEDD        1052
LtaP36.1880 -DDTVN----------ASSASGYARQDSVTDAVCAMQELQNAADNSVFDDYADGGSHEDD        1052
LmjF.36.1900 -DDAVD----------GSSAGGHVRQDSITDAVCAMQELQNAADNSVFDDYADDVDHEDD        1052
LmxM.36.1900 -DDAVD----------DSSAGGHVRQDSITDAVCAMQELQNAADNSVFDDYADDVDQEDD        1052
LdBPK.36.2.001980 -DDAVD----------ASRAGGHVRQDSITDAVCAMQELQNAADNSVFDDYADDVDHEDD        1052
LINF_360025400 -DDAVD----------ASSAGGHVRQDSITDAVCAMQELQNAADNSVFDDYADDVDHEDD        1052
 : : . ::.*:. *:.**:* . .

Tb927.10.6330 ---------FDAVPAPIPLHLQRRNEQKVEVTLTRRPHAN---TTFSSQLQDTVTSSADS        1112
TcCLB.507641.190 AQPSLAQREKQAVYDPL---LQEE-QRK--RQKEEST-----------ASAPVLRAHKSS        1077
LbrM.35.2090 GAYGNDEGASEA-ASPN---LGRPAQADSNVGRGERTAAPMRMRHLPQDITSILRSHAES        1108
LtaP36.1880 DAYGNDEGAYDA--SSG---LGRYAQTESSVRRAEGPPAPLPTRHLPQGITSILRSNAQR        1107
LmjF.36.1900 GAYGNNEGACDA-ASPG---IGRSAQTGGSRDRAEGPSAPMRIRHLPQGITSILRSPARS        1108
LmxM.36.1900 GAYGNDEGACDA-ASPG---LRRSAQTEGSGHRAEGPPAPMQIRHLPQGITSILRSPAQR        1108
LdBPK.36.2.001980 GAYGNDDGACDA-ASPG---LGRSAQTEGSGDRAEGPPAPMRIRHLPQGITSILRSPARS        1108
LINF_360025400 GAYGNDDGACDA-ASPG---LGRSAQTEGSGDRAEGPPAPMRIRHLPQGITSILRSPARS        1108
 :* : . : . : :

Tb927.10.6330 LSPCSSGGSCGLPPAFD-SGTGAADASGRKQGGITPLSKAQERLNESYRQAEGAETIL--        1169
TcCLB.507641.190 LSSKRDQSTMT---SSAGSVGSVRERHPRREVSFVSVDDAEDATKNMNEDPEG-------        1127
LbrM.35.2090 SANQSGRDAAGIEKRPTASVKTFCEANNQTDAGVAPHDGNDSDAEMLNRNGEAGHSSAVA        1168
LtaP36.1880 SPNQIDRGAAGVGKGTMTSGNIYHEGKGQTEVEDVPLDAGGDDVEICSRDDDATHDCKLG        1167
LmjF.36.1900 SSNKSDWGATGAEKGLTTSVNTCRETNRQIDVEDVPHDADGDDAEMRGRDGKTAHSCALG        1168
LmxM.36.1900 SPNKSDRGAAGVEKGSTTSVNMYREANRRTDVEGVPHGADGDDAEMRSRDGEAAHSCALG        1168
LdBPK.36.2.001980 SPDKSDRGAASVETGSTTSVNTYREANRQIDVEDVPHDADGADAEMRSHHGKAAHSCALG        1168
LINF_360025400 SPDKSDRGAASVETGSTTSVNTYREANRQIDVEDVPHDADGADAEMRSRHGKAAHSCALG        1168
 . .: * : : : . . : .. .



Tb927.10.6330 -----------------------TGRAPT-----------------VSKGIQTSPQE--G        1187
TcCLB.507641.190 ------------------ALPLPIAHSTQSAVGNNTAAKDHAYHSLVSRGVQTSYQESRG        1169
LbrM.35.2090 REPRTRSTSRGVQTDVSLTSPAPPGHASQRSVGTSP-------------------LQPAG        1209
LtaP36.1880 VEPRTRSTSRGVQTDVSVTSRALPGDAAQRSVGTSP-------------------IQPVG        1208
LmjF.36.1900 VEPRTRSTSRGVQTDASLNSPALPGHAPQRNVGTSP-------------------IQPAG        1209
LmxM.36.1900 VEPRTRSTSRGVQTDVPLASPALPGNAPQRSVGTSP-------------------IQPAG        1209
LdBPK.36.2.001980 AEPRTRSTSRGVQTDTSLTSPALSGHAPQRSVGTSP-------------------IQPAG        1209
LINF_360025400 AEPRTRSTSRGVQTDTSLTSPALSGHAPQRSVGTSP-------------------IQPAG        1209
 . : : *



Tb927.10.6330 QVKR---------INPATSPNGQLSNVAA--STFPEAVLVES--NPGRSKRFSEMLKEAS        1234
TcCLB.507641.190 VSSSV-VDNSSGRENE--------------SASTMNAGVTQNEM--RRYRQQEPSFIDTK        1212
LbrM.35.2090 TLSHVSVTRRDAPQQPCRAPAEVGSAPAAAPAHTPSSSLYQPQRSHTRPPEADGMLNECS        1269
LtaP36.1880 TSSHISVTRRDNTQQPCHAPAEAVAAPAQS---PSSSSLCQPQRSHTRPPETDNVLSEGT        1265
LmjF.36.1900 TSSHISVTRRNGTQLPCRAPADVGSAR------TPSSPLHQPQRSHTRPPEADGVLSEGT        1263
LmxM.36.1900 TSSQISVTRRDGTQLPCRTPADVGSAH------TPSSSLYQPQRSHTRPPEADGMLSEGT        1263
LdBPK.36.2.001980 TSSHILVTRRDGIQLPCRAPADAGSAH------TLSSSLYQPQRSHTRPPEADSVLSEGT        1263
LINF_360025400 TSSHILVTRRDGIQLPCRAPADAGSAH------TLSSSLYQPQRSHTRPPEADSVLSEGT        1263
 . .: : : * . . : : .



Tb927.10.6330 TAIGDIGANSTWHE--MSVFSEGDEEPIDDTTVPP-LQGTNSANCVTVPSALALQFFATY        1291
TcCLB.507641.190 N--NNDDANNSWREPDLEAENEGDEEPIDNLALPILQASTPHVDGVAIPSGLVLEYLATH        1270
LbrM.35.2090 Q--TPAQRGSTWREPDLADYVDGDTTPIDDYTGVPQLQATATNDGIVLPSGMVLEYLRTH        1327
LtaP36.1880 R--TPAQRGSTWREPDLADYVDGDTTPIDDYTDVPRLQATTTSDGIVLPSGMVLEYLRTH        1323
LmjF.36.1900 R--TPAQRGSTWREPDLVDYVDGDTTPIDDYTGVPRLQATTTSDGIVLPSGMVLEYLRTH        1321
LmxM.36.1900 R--TPAQRGSTWREPDLADYVDGDTTPIDDFTGVPRLQATTTSDGIVLPSGMVLEYLRTH        1321
LdBPK.36.2.001980 R--TPAQRGSTWREPDLADYVDGDTTPIDDYTGVPRLQATTTSDGIVLPSGMVLEYLRTH        1321
LINF_360025400 R--TPAQRGSTWREPDLADYVDGDTTPIDDYTGVPRLQATTTSDGIVLPSGMVLEYLRTH        1321
 ..:*:* : :** ***: : .* : :.:**.:.*::: *:

Tb927.10.6330 QTAGDVFDELQRQDERRHYGECAGPPARRQVLATEGERRGDLAVYGFDRGTASQHNLVST        1351
TcCLB.507641.190 QGAKSVLHELQQFEKELQATRAQA---------------AEVVSYGANNYDEPHQGRVFT        1315
LbrM.35.2090 QGMDSLRHELSQFDQQWMMQQV-----------------AEYVSNNGGVVGA---AGPSS        1367
LtaP36.1880 QGMDSLQHELRQFDQQWMVQQV-----------------AEYVSQNGGMVCA---AGPST        1363
LmjF.36.1900 QGMDSLQHELRQFDQQWMVQQV-----------------AEYVSQNGGMVGA---AGPSS        1361
LmxM.36.1900 QGMDSLQHELKQFDQQWMVQQV-----------------AEYVSQNGGMVGA---AGPSS        1361
LdBPK.36.2.001980 QGMDSLQHELRQFDQQWMMQQV-----------------AEYVSQNGGMVGA---AGPSS        1361
LINF_360025400 QGMDSLQHELRQFDQQWMMQQV-----------------AEYVSQNGGMVGA---AGPSS        1361
 * .: .** : ::. . .: . . . :

Tb927.10.6330 AAVAPHAAILPVVVEGRANNYDLPHSSRPQ-VPGRLVVAHVNMVNTADRKRAQDGGEAEG        1410
TcCLB.507641.190 ATASPHTTILPVTMERRPNNYSVPHATRAP-APSKTVMARLTMIQASERQ-------GQH        1367
LbrM.35.2090 ALRGGVPSLQLVTVESRANNYSRPHADPAELGPSHTVRTEVRMVGPATSYSRPPTQQGHA        1427
LtaP36.1880 ALRGAVPSVQSVTVESRANNYSRPHTDPTQLAPTRTVCTEVCMVGTATSYSRP-RQEVRG        1422
LmjF.36.1900 ALRGGVSSVQSVTVESRANNYSRPHADPTELAPTRTVRTEVRMIGPATSYSRPPRQEEHG        1421
LmxM.36.1900 TIRGGVSSVQSVTVEGRANNYSRPHADPTELAPTRTVCTEVHMIGPATSYSRPPRQEEHG        1421
LdBPK.36.2.001980 ALRGGVSSVQFVTVEGRANNYSRPHADPTELAPTRTVRTEVRMAGPATSYSRPPRQEEHG        1421
LINF_360025400 ALRGGVSSVQSVTVEGRANNYSRPHADPTELAPTRTVRTEVRMAGPATSYSRPPRQEEHG        1421
 : . :: *.:* * ***. **: * : * :.: * : .

Tb927.10.6330 SAC----RMTAEKK-NRVEGAGAGNPGQLVTYSPQPEQGQNGGHHDD----AK---QCYE        1458
TcCLB.507641.190 EVQ---ASIQDENEASVVKKR---RV-----------ED------------ERRGAELSR        1398
LbrM.35.2090 RVVAAAPGLREEEEVDVVDGE---HP--IRAVSGPPGDGNRAGCAGSDEANKRRRVEAGE        1482
LtaP36.1880 CAVEAAPGLHEEEEVDIVDGE---HL--IRAVSSLPDD----------EATKRRRVEATE        1467
LmjF.36.1900 PVVAAAPGLPEEGEVDVVDGE---HP--IRAVSGPPDESDLAGRAGDNDTTKRRRVEATE        1476
LmxM.36.1900 RVVAAAPGLPEEEEVNVVDGE---HP--IRAVSGPPDDSDLAGRAGDDEATKRRRVEATG        1476
LdBPK.36.2.001980 PVVAAAPGLPEEEEVDVVDGE---HP--IRAVSGPPDDSDFAGGAGDDETTKRRRVEATE        1476
LINF_360025400 PVVAAAPGLPEEEEVDVVDGE---HP--IRAVSGPPDDSDFAGGAGDDETTKRRRVEATE        1476
 . : * : . *. . : : :



Tb927.10.6330 NRGATTTLL-------------------PQQETSAALRELEAL-----------------        1482
TcCLB.507641.190 NDAKKAMLQTPAA--KMGTGAQFFLHQS----VSSVMKDLGGMRRQVEAMNSV-------        1445
LbrM.35.2090 GNP-TTPLPPPVSPTGAFRGRNFFLNQCTQQEVSSTLQDIHYLQRQQQASMSALTEAQRA        1541
LtaP36.1880 GNT-TTPLPPPVSPSSAFRGRNFFLNQHTQQDVGSTLQDIHYLQKRQQANISALAKAQST        1526
LmjF.36.1900 GSA-TTPLPPPVSPTSAFRGRNFFLNQHTQQEVDSTLQDIRYLQRRQQTNMSALAKAQSA        1535
LmxM.36.1900 GNA-TTPLPPPVSPVSAFRGRNFFLNQHTQQEVGSTLQDIHYLQRRQQANMSALAKAQSA        1535
LdBPK.36.2.001980 GNA-TTPLPPPVSPTSAFRGRNFFLNQHTQQEVSSTLQDIHYLQRRQQANMSALAKAQSA        1535
LINF_360025400 GNA-TTPLPPPVSPTSAFRGRNFFLNQHTQQEVSSTLQDIHYLQRRQQANMSALAKAQSA        1535
 . .: * ..:.:::: :

Tb927.10.6330 ------LGHSGATSVSPTFGG---EVATTTSGGVAMFPQGVVPRFFVEQGTGTTTLELRQ        1533
TcCLB.507641.190 --------------MTPN----QGGTIGVPLNQVTPYGQMVLPECFVEQNNDTAVREIRQ        1487
LbrM.35.2090 GETVEAADDDEVPRKTLHQGQSSTGVASEGVPPATPYGQVILPTWIVEQRNDTAIRELRQ        1601
LtaP36.1880 TETAELAGDDEAPRKTPHQRQTPTDVAGEGVPPTTPYGQVILPTWVVEQRNDTAIRELRQ        1586
LmjF.36.1900 AETVESAGDDGAPRKTPHQGQSSTGVAGEGVPPTTPYGQLILPTWIVEQRNDTAVRELRQ        1595
LmxM.36.1900 AETAESAGDDEAPRKTPHQGQSSTGVAGEGVPPTTPYGQVILPTWIVEQRNDTAIRELRQ        1595
LdBPK.36.2.001980 AETAESTGDDEAPRKTPHQGQSSTGVAGEGVPPTTPYGQVILPTWIVEQRNDTAIRELRQ        1595
LINF_360025400 AETAESTGDDEAPRKTPHQGQSSTGVAGEGVPPTTPYGQVILPTWIVEQRNDTAIRELRQ        1595
 : . .: : * ::* .*** ..*: *:**

Tb927.10.6330 AMGARDPNGVSSLTNNERRVRGNGIMGANVDGEVGNTWRSDVTAPPVPEYARDPQYSLEI        1593
TcCLB.507641.190 VMGTHNPNDSRLSTGGRRKIRGTGGSIIGE-LENSAAWWDEKSSAPMPQFATDPQYSMEL        1546
LbrM.35.2090 VMGAHNPNDVRLSASAGKRSRIHGSGTGDG-SGNSAAWWAEMSSAPMPNYAADPQYSMEL        1660
LtaP36.1880 VMGAHNPNDVRLSTSAGKRSRIHGSGTGGG-SGNSTAWWAEMSSAPMPNYASDPQYSMEL        1645
LmjF.36.1900 VMGAHNPNDSRLSTSAGKRSRIHGSGAGDG-SGNSAAWWAEMSSAPMPNYAADPQYSMEL        1654
LmxM.36.1900 VMGAHNPNDSRLSTSAGKRSRIRGSGTGDG-SGNSAAWWAEMSSAPMPNYAADPQYSMEL        1654
LdBPK.36.2.001980 VMGAHNPNDSRLSTSAGKRSRIHGSGTGDG-SGNSAAWWAEMSSAPMPNYAADPQYSMEL        1654
LINF_360025400 VMGAHNPNDSRLSTSAGKRSRIHGSGTGDG-SGNSAAWWAEMSSAPMPNYAADPQYSMEL        1654
 .**:::**. :. :: * * . . :* : :: *:*::* *****:*:

Tb927.10.6330 V        1594
TcCLB.507641.190 F        1547
LbrM.35.2090 F        1661
LtaP36.1880 F        1646
LmjF.36.1900 F        1655
LmxM.36.1900 F        1655
LdBPK.36.2.001980 F        1655
LINF_360025400 F        1655
 .

# **KKT2**

LbrM.35.5600 ---------------------------------------MSHFRGSFSRTPPRSGAISMP        21
LtaP36.5470 ---------------------------------------MSHFCSSLSRTPPRGGVVSMP        21
LmxM.36.5350 ---------------------------------------MSHFCGSLSRTPPRGGAISMP        21
LmjF.36.5350 ---------------------------------------MSHFCRSLSRTPPRGGAISMP        21
LINF_360063000 ---------------------------------------MSHFCSSLSRTPPRGGAISMP        21
LdBPK.36.2.005580 ---------------------------------------MSHLCSSLSRTPPRGGAISMP        21
Tb927.11.10520 -------------------------------MFNVSPASRDRVRSESQRTPRPRSSLSMP        29
TvY486_1111400 -------------------------------MTSPPLAGSNSADDSLVKPASSRGAVSIP        29
TcCLB.510285.70 MSLLEKKQDNSGGSSNSNSSSSGGDVKSRKDNDGKTEEGGE-EKDGRLRTPRSYAMLSIP        59
 . : . :*:*



LbrM.35.5600 RDLSQTPAISRLGSTVKTPHIQKCVVDQT------------EDDDHPLEHMTVYFEEEDL        69
LtaP36.5470 RDLSQTPAISRLGSTVKTPHIQKCVVDQT------------EDDDHPLEHMTVHFEEEDL        69
LmxM.36.5350 RDLSQTPAISRLGSTVKTPHIQKCVVDQA------------EDDDHPLEHMTVYFEEEEL        69
LmjF.36.5350 RDLSQTPAISRLGSTVKTPHIQKCVVDQT------------EDDDHPLEHMTVYFEEENL        69
LINF_360063000 RDLSQTPAISRLGSTVKTPHIQKCVVDQT------------EDDDHPLEHMTVYFEEEDL        69
LdBPK.36.2.005580 RDLSQTPAISRLGSTVKTPHIQKCVVDQT------------EDDDHPLEHMTVYFEEEDL        69
Tb927.11.10520 RELSYTPAISSIPSSLHTPFIQKCYVQGDNST-----EGQQQQQQQPPDHMTVVFESDNM        84
TvY486_1111400 RELSHTPGIACISSTMRTPSIQRCRVDRGGESDDADSDMQKQREPQPIEQMVVTFESDGL        89
TcCLB.510285.70 RELSHTPAIAGLSPTVRTPFIQRCHLHHGDYTSTD---NKDEHYRQPLEHMTIIFEEDSL        116
 *:** **.*: : :::** **:* :. : :* ::*.: **.: :



LbrM.35.5600 RLVTTGLLGKGGFGKVFDAVSNSGDAYALKVSSKRMSENDWKRLKEEVTLMSHFSRHPNI        129
LtaP36.5470 RLVTTGLLGKGGFGKVFDAVSNSGESYALKVSSKRMSENDWKRLKEEVTLMSHFSRHPNI        129
LmxM.36.5350 RVVTTGLLGKGGFGKVFDAVSNSGEAYALKVSSKRMSENDWKRLKEEVTLMSHFSRHPNI        129
LmjF.36.5350 RLVTTGLLGKGGFGKVFDAVSNSGEAYALKVSSKRMSENDWKRLKEEVTLMSHFSRHPNI        129
LINF_360063000 RLVTTGLLGKGGFGKVFDAVSNSGEAYALKVSSKRMSENDWKRLKEEVTLMSHFSRHPNI        129
LdBPK.36.2.005580 RLVTTGLLGKGGFGKVFDAVSNSGEAYALKVSSKRMSENDWKRLKEEVTLMSHFSRHPNI        129
Tb927.11.10520 QLTTTSLLGKGGFGRVYVAQSSGGELCALKVSSKQMTDGDWERLRKEVALMSHFSQHPNV        144
TvY486_1111400 QLTTASLLGKGGFGHVYAAYSNGGELYAMKVSSKKMSENDWERLRKEVTLMSHFSRHPNV        149
TcCLB.510285.70 QLTTTSLLGKGGFGRVYAARSNGGELYALKVSAKKLTEGDWARLLKEVELMNHFSRHPNV        176
 ::.*:.********:*: * *..*: *:***:*::::.** ** :** **.***:***:

LbrM.35.5600 VKFYAAGKDEDRAYVVMERCAGKSLHDIIASRSLDVPEILWIGWALISTISYIHSKGCIH        189
LtaP36.5470 VKFYAAGRDQDRAYVVMERCAGMSLHDVIASRSLDVPEILWIGWALVNTISYIHSKGCIH        189
LmxM.36.5350 VKFYGAGRDEDRAYVVMERCAGKSLHDVIASRSLDVPEILWIGWALVNTISYIHSKGCIH        189
LmjF.36.5350 VKFYAAGRDEDRAYVVMERCAGKSLHDVIASRSLDVPEILWIGWALVNTISYIHSKGCIH        189
LINF_360063000 VKFYAAGRDEDRAYVVMERCAGKSLHDVIASRGLDVPEILWIGWALVNTISYIHSKGCIH        189
LdBPK.36.2.005580 VKFYAAGRDEDRAYVVMERCAGKSLHDVIASRGLDVPEILWIGWALVNTISYIHSKGCIH        189
Tb927.11.10520 VKLIAAGRDRNFAYVAMECCASRSLHDIINKHGLEVPEILWVGYALIDTIAFLHAKGCIH        204
TvY486_1111400 VKMIAAGCDSERAYVVMECCASRSLHDIIAQCGLDVPEILWIGWALVDTVAFLHAKNCIH        209
TcCLB.510285.70 VKFIAAGRDEETAYVVMECCASRSLHDVIANHGLDVPEILWVGWALVDTIAFMHSKGCIH        236
 **: .** * : ***.** **. ****:* . .*:******:*:**:.*::::*:*.***

LbrM.35.5600 RDLKPQNLLFDTEGNLKITDFGLSSRISEAHPRKTVAGTAMYMAPEMAVEVYKRMTQNSD        249
LtaP36.5470 RDLKPQNLLFDNNGNLKITDFGLSSRISEAHPRKTVAGTAMYMAPEMATEVYKRLTKNSD        249
LmxM.36.5350 RDLKPQNLLFDNEGNLKITDFGLSSRISEAHPRKTVAGTAMYMAPEMATEVYKRMTKNSE        249
LmjF.36.5350 RDLKPQNLLFDSEGSLKITDFGLSSRISEAHPRKTVAGTAMYMAPEMATEVYKRMTKNSD        249
LINF_360063000 RDLKPQNLLFDHEGNLKITDFGLSSRISEAHPRKTVAGTAMYMAPEMATEVYKRMTKNSD        249
LdBPK.36.2.005580 RDLKPQNLLFDHEGNLKITDFGLSSRISEAHPRKTVAGTAMYMAPEMATEVYKRMTKNSD        249
Tb927.11.10520 RDLKPQNLLFDFDGNLKISDFGLSSNVTESEPRKTVAGTAMYMAPEIAGAVYKRMTNDNS        264
TvY486_1111400 RDLKPQNLLFDFSGNLKITDFGLSSSIVDAQPRKTVAGTAMYMAPEIADVVYTKMTNPDK        269
TcCLB.510285.70 RDLKPQNLLFDFDGNLKITDFGLSSRIAEAQPRKTVAGTAMYMAPEIAEAVYKRMSHSTH        296
 *********** .*.***:****** : ::.***************:* **.::::

LbrM.35.5600 APSLSYGKEVDTWSIGVVLYVLLTRMNPYLEAMEQKGMHQLNKGHKSLALFNAVAGAAWS        309
LtaP36.5470 APSLSYGKEVDTWSIGVVLYVLLTRMNPYLEAIEHKGMRQLDKEHKSLALFNAVAGAAWS        309
LmxM.36.5350 APSLSYGKEVDTWSIGVVLYVLLTRMNPYLEAIEQKGMRQLDKEHKSLALFNAVAGAAWS        309
LmjF.36.5350 APSLSYGKEVDTWSIGVVLYVLLTRMNPYLEAIEQKGMRQLDKEHKSLVLFNAVAGAAWS        309
LINF_360063000 APSLSYGKEVDTWSIGVVLYVLLTRMNPYLEAIEQKGMRQLDKEHKSLALFNAVAGAAWS        309
LdBPK.36.2.005580 APSLSYGKEVDTWSIGVVLYVLLTRMNPYLEAIEQKGMRQLDKEHKSLALFNAVAGAAWS        309
Tb927.11.10520 SSSLRYGQEVDTWSIGVVLYVMLTRMNPYAQALENEGAHEMNKTQKTLTLFSAVAGAAWH        324
TvY486_1111400 KNSLQYGQEVDTWSIGVVLYVMLTRMNPYVQAMETRCAHTMDKTQKTLTLFNAVADAAWQ        329
TcCLB.510285.70 QPPLQYGQEVDTWSIGVVIYVMLTRMNPYMEAMEKRGTREMDKTQKTLSLFNAVADAAWE        356
 * **:**********:**:******* :*:* . : ::* :*:* **.***.***

LbrM.35.5600 WPREWKGDPQLCRLVERVLHREASRRATLMEVLEDSVWNRRPLSCPLSLLQKLNLLEPSP        369
LtaP36.5470 WPKEWRGDPQLCRLVERVLHCEPSRRATLMEVLEDSVWNRRPLSCPLSLLQKLNLLEPSP        369
LmxM.36.5350 WPREWRGDPQLCGLVERMLHREPSRRATLMEVLEDSVWNRRPLSCPLSLLQKLNLLEPSP        369
LmjF.36.5350 WPREWRGDPQLCRLVERVLHREPSRRATLMEVLEDSVWNRRPLSCPLSLLQKLNLLEPSP        369
LINF_360063000 WPREWRGDPQLCRLVERVLHREPSQRATLMEVLEDSVWNRRPLSCPLSLLQKLNLLEPSP        369
LdBPK.36.2.005580 WPREWRGDPQLCRLVERVLHREPSQRATLMEVLEDSVWNRRPLSCPLSLLQKLNLLEPSP        369
Tb927.11.10520 WPAGWRGDKELSDVVNQILHRNPAQRATLQDILQHPVWDRRPLSCPLTLLQKLNLVERRP        384
TvY486_1111400 WPKGWSGDAELCEIVNRTLHRDPAKRATLEELQQHTVWNRRPLSCPLSLLQKLNLLGKPS        389
TcCLB.510285.70 WPTGWSGDPELCDLVNWALHRDTKKRATLQEVLQHPVWNRRPLSCPLSLLYKLNLLERPS        416
 ** * ** :*. :*: ** : :**** :: :. **:********:** ****:

LbrM.35.5600 SGALP--------LNNLAENLQF-RPKRTTEAVLREGLERVEATEQRGRAQVELEYYETY        420
LtaP36.5470 ASGLP--------LNHLAENLQF-RPKRSAEAVLREGRERIEATEQRGRAQLELEYYESF        420
LmxM.36.5350 SSGLP--------LNNLAENLQF-RPKRSAEAVLREGLERVEATEQRGRAQLELEYYETY        420
LmjF.36.5350 SSGLP--------LNNLAENLQF-RPKRSAEAVLREGLERIEATEQRGRAQLELEYYETY        420
LINF_360063000 SSGLP--------LNNLAENLQF-RPKRSAEAVLREGLERIEATEQRGRAQLELEYYETY        420
LdBPK.36.2.005580 SSGLP--------LNNLAENLQF-RPKRSAEAVLREGLERIEATEQRGRAQLELEYYETY        420
Tb927.11.10520 FTRT---GAVGRSCSRAPEGMQPQYATKTAEVVLLEGLNQVIQVEESTRTQLVLESNEVI        441
TvY486_1111400 SSRS---SVTRRSTSRVQEHAHLPVARRTPSAVIEEALTRVVTTERRARSQLALEHHETL        446
TcCLB.510285.70 SSSVSRHGSKRRSLTRLADNLPIPTGKRTADDVLAEGMRRVVLTEQRARANLVLEHHETL        476
 .. : :: . *: *. :: .*. *::: ** *

LbrM.35.5600 NVLWSLLTLARAEVDTRADILQSEVVQRGKLRNQFLTRQSTRDRGGSVSLVSEVADCETT        480
LtaP36.5470 NVLWSLLTLGRAEGDARADILQSEEVQRGKLRNQSMSRRSARGRCGSVSLVSEAADGETV        480
LmxM.36.5350 NVLWSLLTLARAEEDARADILQSEEVQRGKLRNQSLARQSARRRCGSVSLVSEVADREEA        480
LmjF.36.5350 NVLWSLLTLARAEEDARVDILQSEEVQRGKVRNQFLARQSARGRCGSVSLVSEAADREVA        480
LINF_360063000 NVLWCLLTLARAEEDARADILQSEEVQRGKLRNQSLARQSARGRCGSVSLVSEVADREVA        480
LdBPK.36.2.005580 NVLWCLLTLARAEEDARADILQSEEVQRGKLRNQSLARQSARGRCGSVSLVSEVADREVA        480
Tb927.11.10520 NLIFGTLKLHASEANGRRFIVIDEKGCRKNIEDMLLTTRPVRCRSHELVSTAVCAKQPKR        501
TvY486_1111400 QIIVEMLRLSTDELDCRRNIEVEEKRHRLTIVNHRTMHKPKRRSVNNPTTSSLPRTCAVE        506
TcCLB.510285.70 RVMLELLKLSRAEADVRRSICEDEQRYRTVIQNQRVTQRSRRRRSEALVSSFSAATVPTE        536
 .:: * * * : * * .* * : : : *

LbrM.35.5600 TPRASRSV---RR---------SVSLTEEERGRIVRSSPVQYAVVYPGRDTATRWNLRAV        528
LtaP36.5470 APRTSRSV---RR---------SVSLTEEERGRIVRSSPVQYAVVYPGRDTATRWNLRAV        528
LmxM.36.5350 APRTSRSV---RR---------SVSLTEQERGRLVRSSPVQYAVVYPGRDTATRWNLRAV        528
LmjF.36.5350 ASRTSRSV---RR---------SVSLTEQERGRLVRSSPVQYAVVYPGRDTATRWNLRAV        528
LINF_360063000 ASRTSRSV---RR---------SVSLTEQEQGRLVRSSPVQYAVVYPGRDTATRWNLRAV        528
LdBPK.36.2.005580 ASRTSRSV---RR---------SVSLTEQEQGRLVRSSPVQYAVVYPGRDTATRWNLRAV        528
Tb927.11.10520 GTRVGSSLRPQRESSVLLAGPLAF-GQTQDAGEIVRAASDRYAVVFSGRETSTRWSLRNV        560
TvY486_1111400 GSEAVGRSRVSRQPSVSLVSPSSPPRVKEEECEIVRATPDKFAVVYPGRESSTRWSLRPV        566
TcCLB.510285.70 AGPVTRSSRSHRQSSVVLVAPAA--STKEESGRIVRASPDKYAMVYPGRDTATRWSLRPV        594
 . *. : :: .:**:: ::*:*: **:::***.** *



LbrM.35.5600 VSLPRDMTDEIEREFKCMNGHLMTKLTSMPHGYNGFDCNVCDRGILKITGESPAFRCYKC        588
LtaP36.5470 VSLPRDMTDEIEREFKCMNGHVMTKLTSMPHGYNGFDCNVCDRGILKITGESPAFRCYKC        588
LmxM.36.5350 VSLPRDMTDEIEREFKCMNGHVMTKLTSMPHGYNGFDCNVCDRGILTITAESPAFRCYKC        588
LmjF.36.5350 VSLPRDMTAEIEREFKCMNGHVMTKLTSMPHGYNGFDCNVCDRGILKITGESPAFRCYKC        588
LINF_360063000 VSLPRDMTAEIEREFKCMNGHVMTKLTSMPHGYNGFDCNVCDRGILKITGESPAFRCYKC        588
LdBPK.36.2.005580 VSLPRDMTAEIEREFKCMNGHVMTKLTSMPHGYNGFDCNVCDRGILKITGESPAFRCYKC        588
Tb927.11.10520 ISLPRELNAAIE-EVTCINKHQMKKLIKIPLGYVGFDCNVCDSAIDDISIEKPVFRCHKC        619
TvY486_1111400 VSLPRDLNPEIE-SFKCMNNHIMTKLTKMPNAYVGFDCNVCDREILKITPETPAFRCYKC        625
TcCLB.510285.70 ISLPRELNSEIE-EFRCLNHHIMTKLTAMPHGYNGFDCNVCDREILKISPETPAFRCYKC        653
 :****::. ** .. *:* * *.** :* .* ******** * *: *.*.***:**



LbrM.35.5600 DYDVCMKCAYSGKFKDVSFVCVTCAKRFTSTAKLQGHSLRCRGPSESPSPRRSSRMNTML        648
LtaP36.5470 DYDVCMKCAYSGKFKDVNFVCVTCAKRFTSTAKLQGHSLRCRGPSESPSPRRSSRMNTML        648
LmxM.36.5350 DYDVCMKCAYSGKFKDVNFVCVTCAKRFTSTAKLQGHSLRCRGPSESPSPRRSSRMNTML        648
LmjF.36.5350 DYDVCMKCAYSGKFKDVNFVCVTCAKRFTSTAKLQGHTLRCRGPSESPSPRRSSRMHTML        648
LINF_360063000 DYDVCMKCAYSGKFKDVNFVCVTCAKRFTSTAKLQGHTLRCRGPSESPSPRRSSRMNTML        648
LdBPK.36.2.005580 DYDVCMKCAYSGKFKDVNFVCVTCAKRFTSTAKLQGHTLRCRGPSESPSPRRSSRMNTML        648
Tb927.11.10520 DYDLCMNCAYEGKIKDVNFVCVSCMKKFASSSKLEAHSAKCRGPSMSPSARCSSRRNTML        679
TvY486_1111400 DYDLCLKCAYNHRLKDVSFVCVSCAKKFISAAKLQAHSLQCRGPSVSASPRRCSRVNTML        685
TcCLB.510285.70 DYDLCMRCAYQGRLKDVNFVCVSCAKKFTSSAKLQAHSLQCRGPSWSPSPRRSSRMNTML        713
 ***:*:.***. ::***.****:* *:* *::**:.*: :***** * * * .** :***

LbrM.35.5600 WDE--PKRPSLLEVQLPEASQSESKLHASRCRSGRRTYNRTSTGGRISIGDPNVHSVVDF        706
LtaP36.5470 WEE--PKRPSLLEVQLPEAPQSEPKLGASRCRSGRPTYNRTSTGGRISIGDPNAHSVVDF        706
LmxM.36.5350 WDE--PKRPSLLEVQLPEAPQSERKLRASRCRSGRPTYNRTSTGGRISIGDSNAHSVVDF        706
LmjF.36.5350 WDE--PKRPSLLEVQLPEVPQSEPKLRDSLCRSGRPTYNRTSTGGRISIGDPNAHSVVDF        706
LINF_360063000 WDE--PKRPSLLEVQLPEAPQSEPKLRASRCRSGRPTYNRTSTGGRISIGDPNAHSVVDF        706
LdBPK.36.2.005580 WDE--PKRPSLLEVQLPEAPQSEPKLRASRCRSGRPTYNRTSTGGRISIGDPNAHSVVDF        706
Tb927.11.10520 WEQLENEGGSLLDIRLPTETK----PRRSARTSCRTSGGRESSGGRISIGDSYVPGPEDL        735
TvY486_1111400 WEEAEMVGGSLLDVRLPNSAAAAPGDRRSTRTSGLSSFMRMSSGGLISTGDSHVALPEDV        745
TcCLB.510285.70 WEEAEPRRGSLLEVRLPDDPK----PRASGRPSGRSSTGRASSGGRISIGDSHVASPEDV        769
 *:: ***:::** * * : * *:** ** ** . *.

LbrM.35.5600 DAMVAEHREADFPDVSACASATGRELSQQ-RKLAGNRRGRPSTMSSGSLSLDLPPQVQVP        765
LtaP36.5470 DAMVAEHREADFPEVSARMSITGRESSQK-RVRAGSQRGRPSTSSSGSLSLDLPPQVQLP        765
LmxM.36.5350 DAMVASHREADFPKVSTRASATGRESSQR-RERTGSGRGRPSTSSSGSLSLDLPPQVQVP        765
LmjF.36.5350 DAMVAEHREADFPKVSARAPATGRESSQK-RERAGSRRGRPSTSSSGSLSLDLPPQVQVP        765
LINF_360063000 DAMVAEHREADFPKVSARASATGRESSQK-RERAGSRRGRPSTSSSGSLSLDLPPQVQVP        765
LdBPK.36.2.005580 DAMVAEHREADFPKVSARASATGRESSQK-RERAGSRRGRPSTSSSGSLSLDLPPQVQVP        765
Tb927.11.10520 GTMVVAHRDANFPEMPKFSTQSDSKAS-----SPFGEKEGADKNYRQSFSFELPPQVRLS        790
TvY486_1111400 GKMVVQHRDASFPDVPNFSPPEVVVESRKGATS-SGVR-KDATRVSRSVSFELPPEVRFP        803
TcCLB.510285.70 GTMVVQHRDASFPEMPFTRRAGSG----GGRRSTPARV-HGCEDVNSSFSFELPPEIRLS        824
 . **. **:*.**.: *.*::***:::.

LbrM.35.5600 SKGSRAPVQPRSSAELRDIMEEVEQQKQAMRRDPLL---YGPATPPQYNSNGEIIGIAAR        822
LtaP36.5470 SKASCTQVQPRSSAELRDIMEEVRQQKQILPRDPLL---SGPATPPQYNSNGEIIGIAAR        822
LmxM.36.5350 SKESRPQVQPRSSAELRDIMEEVEQRKQALPRDPLL---SAPATPPQYNCNGEIIGISAR        822
LmjF.36.5350 SKESRAQVEPRSSAELRDIMEEVEQRKQALPRDPLL---SGPATPPQYNCNGEIIGIAAR        822
LINF_360063000 SKESRAQVQPRSSAELRDIMEEVEQRKQALPRDPLL---SGPATPPQYNCNGEIIGIAAR        822
LdBPK.36.2.005580 SKESRAQVQPRSSAELRDIMEEVEQRKQALPRDPLL---SGPATPPQYNCNGEIIGIAAR        822
Tb927.11.10520 KHRRDKGAEPRSSEELREIVDELTPTPKRVRTEARQNSC--EPYAPQVNDAGLVVGIAAR        848
TvY486_1111400 KQDREDKVQPRDSAELKEILEADQPPLKKRRSQSLQ-KDDNNVFSYEVNSSGTIIGIAAR        862
TcCLB.510285.70 RLKKGERIQPRTSAEMQEVMDVDSPPLKKERKEARGGGQEKDTPPYKVNESGNIVGIAAQ        884
 :** * *:::::: : : : * * ::**:*:



LbrM.35.5600 RRAESVEM---------TRAEVITIRAEVANRPHELQRQPRVPRSASSSRLENAPQSLHK        873
LtaP36.5470 RRAESLEV---------ARAEVITIRAEVADRPRELQHQPRVPRSASSTRVEKGLRSPHK        873
LmxM.36.5350 RRAESLEM---------ARAEVITIRAEVADRPRELQHQPRVPRSASSSRAEKGLPSPHK        873
LmjF.36.5350 RRAESLEM---------ARAEVITIRAEVADRPRELQHQPRVPRSASLSRAEKGLQSPHK        873
LINF_360063000 RRAESLEM---------ARAEVITIRAEVADRPRELQHQPRVPRSASSSRAEKGLQGSHK        873
LdBPK.36.2.005580 RRAESLEM---------ARAEVITIRAEVADRPRELQHQPRVPRSASSSRAEKGLQGSHK        873
Tb927.11.10520 SRVEREGSLRGEQQ----SRNVVVIRADNAAKPQDLRKQSQVPRSASSSQPGSRMESQAI        904
TvY486_1111400 HRVAREGSLLSTEKRQGGPTNVVVIRAENAQKPEEKSRQTRVPRSASASRVAVSAAPSAT        922
TcCLB.510285.70 HRAAREASLQQQQKQQGQ--GVIVIRADAAAKPPEMSRQPQVPRSVSSSRSAGAVSSVAK        942
 *. *:.***: * :* : :* :****.* ::



LbrM.35.5600 RRREEWQQPVHAPSPSGAVNRVAVEE----HVAKHAVLPPQVPRGKGQQPRAPSVSGHTA        929
LtaP36.5470 RCREEWQHPVHTSSSSGTAKRAAVEE----QIVKHTAVPPQVPRGQAQQPRAPSVSGHTA        929
LmxM.36.5350 RRREEWQQPAHAPSPSGTAKRAAVEE----HVVKQAIMPPQVPRGRAQQPRAPSVSGHTA        929
LmjF.36.5350 RRREEWQQPVHAPSSSGTAKRAAVEE----HVVKHAIMPPEVPRGRAHQQRAPSVSGHTA        929
LINF_360063000 RRREEWKQSAHAPSPSGTAKRAAVEE----HVAKHAIMPPQVPRGRAQQSRAPSVSGHTA        929
LdBPK.36.2.005580 RRREEWKQSARAPSPSGTAKRAAVEE----HVAKHAIMPPQVPRGRAQQSRAPSVSGHTA        929
Tb927.11.10520 DTLSKPAPKN-----VAVREYR--SAS--------GQRDPLVP--R---TSLPSAQTGEI        944
TvY486_1111400 HSTSKEDA----------AHRT-------------AASVP-----P---TRQSSARTSAL        951
TcCLB.510285.70 NVAPKPIEPH-----LAKVTRTAIPAPTPTNTTAPAAPMPNMPKKG---RQPSAGGMVTL        994
 : * :



LbrM.35.5600 QGGPPLPRRGPAAPSPAATLKTHLSAFQIPAAIPPKNFTSIRQSSYSMANTMAATYSASA        989
LtaP36.5470 QGGPPLPRRGPAAPSPAAALKAHLSALQVPAAIPPKNFSSILQSRYSMTNAMAPTHNTST        989
LmxM.36.5350 QGGPPLPRRGPAAPSPAAALKAHLSPFQAPAAIPPKNFASILQSRYSMTNAMAPTCNTST        989
LmjF.36.5350 QGGPPLPRRGPAAPSPAAALKAHLGVFQAPAAIPPKNFASILPSRYSMTNAMAPTCNIST        989
LINF_360063000 QGGPPLPRRGPAAPFPAAALKAHLGAFQVPAAIPPKNFASILPSRYSMTNAMAPTCNTST        989
LdBPK.36.2.005580 QGGPPLPRRGPAAPFPAAALKAHLGAFQVPAAIPPKNFASILPSRYSMTNAMAPTCNTST        989
Tb927.11.10520 FGAPPLPRIVTSGRSSVPRS--------MSYTLPSAAGVARRQTSQPLVNLLGGV-----        991
TvY486_1111400 CGGPPLPRRTPGASSPVQKI------------VPTSNFVVPTQPLQPPPSTTERGRVKAV        999
TcCLB.510285.70 HGGPPLPRRGPGMPQNTPYL--------VPQAVPPPNFPVPRQPSHPLVQSGVE------        1040
 *.***** . . :* .

LbrM.35.5600 TE-----PADRAGAANAALG---YGAASTRSHALSRPNGAFLALPREEHSRRQFLDDFLS        1041
LtaP36.5470 -R-----LTDGAGTATAPLG---QRGVPTYSHALSRPNGAFLALPREERNRQQFLDDFLS        1040
LmxM.36.5350 TR-----PAGGAGAATAALG---QGGAPTYSHALSRPNGAFLALPREERNRQQFLDDFLS        1041
LmjF.36.5350 TR-----PADGAGAATVALG---QGGAPTYSHVLGRPNGAFLALPREERNRQQFLDDFLS        1041
LINF_360063000 TR-----PADGAGAAAADLG---RGGAPTYSHVLGRPNGAFLALPREERNRQQFLDDFLS        1041
LdBPK.36.2.005580 TR-----PADGAGAAAADLG---RGGAPTYSHVLGRPNGAFLALPREERNRQQFLDDFLS        1041
Tb927.11.10520 -PV----GAYGGGSFNPGERNAS-ISNTNRTVGPGQGGTAYLALPRDKQNRDRFVDDFLS        1045
TvY486_1111400 TSVSDYLGTAGRGADSVSANQVNIASCVPAVGGVGGGGKAYLALPRDEHNRIRFVEDFLS        1059
TcCLB.510285.70 ------------------------ARQVVGGHRVSGGNSAFLALPREEQNRQRFVDDFLS        1076
 . . *:*****:::.* :*::****

LbrM.35.5600 GGWVRFYSFTNEDTVVMYYSLQPGRYGAMFPTEAGVGTAVLDVYAKLVLYVPCMNNESTN        1101
LtaP36.5470 GGWVRFYSFTNEDTVVMYYSLQPGRYGAMFPTEAGVGTAVLDVYSKLVLYVPCMNNESTN        1100
LmxM.36.5350 GGWVRFYSFTNEDTVVMYYSLQPGRYGAMFPTEAGVGTAVLDVYSKLVLYVPCMNNESTN        1101
LmjF.36.5350 GGWVRFYSFTNEDTIVMYYSLQPGRYGAMFPTEAGVGTAVLDVYSKLILYVPCMNNESTN        1101
LINF_360063000 GGWVRFYSFTNEDTVVMYYSLQPGRYGAMFPTEAGVGTAVLDVYSKLVLYVPCMNNESTN        1101
LdBPK.36.2.005580 GGWVRFYSFTNEDTVVMYYSLQPGRYGAMFPTEAGVGTAVLDVYSKLVLYVPCMNNESTN        1101
Tb927.11.10520 GAWVRVYSFIGSEVVVMYYSVQPGRYGALFPTEEGAATAVLDIHSKLVLYVPRMDKDTVT        1105
TvY486_1111400 GGWVRFYSFTKDDVVVMYYCLQPGRYGAMFATEAGFGTAVVDVYSKLVLYVPCVNNDGTN        1119
TcCLB.510285.70 GGWVRFYSFTNEETVVMYYCAQPGRYGAMFPTEAGVGTAVVDVHSRLVLYVPCMNNESTN        1136
 *.***.*** .:.:****. *******:* ** * .***:*::::*:**** :::: ..

LbrM.35.5600 RSQPHPHVQTFYDEEARILSLTEAQRYLGGVLRCITGFVDEFSRLKSEGLTPAAVHAAYI        1161
LtaP36.5470 RNQPHPHVQTFYDEEARILTLPEAQRYLGGVLRCITGFVDEFSRLKAEGLTPAAVHAAYI        1160
LmxM.36.5350 RSQPHPHVQTFYDEEARILSLPEAQRYLGGVLRCITGFVDEFSRLKAEGLTPAAVHAAYI        1161
LmjF.36.5350 RSQPHPHVQTFYDEEARILSLPEAQRYLGGVLRCITGFVDEFTRLKAEGLTPAAVHAAYI        1161
LINF_360063000 RSQPHPHVQTFYDEEARILSLPEAQRYLGGVLRCITGFVDEFSRLKAEGLTPAAVHAAYI        1161
LdBPK.36.2.005580 RSQPHPHVQTFYDEEARILSLPEAQRYLGGVLRCITGFVDEFSRLKAEGLTPAAVHAAYI        1161
Tb927.11.10520 RTQCHPNVLSFFQDEIRILPATSAEKTLGGVLRGIMGFVSELTKCRGEGEKYAAAQSAYI        1165
TvY486_1111400 RSQPHPHVQTFFEEEVRLLSISEAKRYLDNVLNNIMDFVREITRLRAEGLTPAAVHAAYI        1179
TcCLB.510285.70 RSQPHPHVQTFYDEDVRLLSVSEAQRHLGGVLESIMGFVNEVARLRAEGLTPAAVHAAYI        1196
 *.* **:* :*:::: *:* .*:: *..**. * .** *.:: :.** . **.::***

LbrM.35.5600 HHRSMSHVPRDTKFVYIRKVFPDPSGSFTLFRLSNLRSQVVC-NAMVDIRWQSDRRHNVG        1220
LtaP36.5470 HHRSMTHVPRDTKFVYIRKVFPDPSGSFTLFRLSNLRSQVVC-NAMVDIRWQSDRRHNVG        1219
LmxM.36.5350 HHRSMSHVPRDTKFVYIRKVFPDPAGSFTLFRLSNLRSQVVC-NAMVDIRWQSDRRHNVG        1220
LmjF.36.5350 HHRSMSHVPRDTKFVYIRKVFPDPSGSFTLFRLSNLRSQVVC-NAMVDIRWQSDRRHNVG        1220
LINF_360063000 HHRSMSHVPRDTKFVYIRKVFPDPSGSFTLFRLSNLRSQVVC-NAMVDIRWQSDRRHNVG        1220
LdBPK.36.2.005580 HHRSMSHVPRDTKFVYIRKVFPDPSGSFTLFRLSNLRSQVVC-NAMVDIRWQSDRRHNVG        1220
Tb927.11.10520 HQREKGAVPAGTKFAYVRKAFPDPAGSFVLFRLSNLRSQVVFNNALLDIRWQSDKNHNVG        1225
TvY486_1111400 YQRDKNSVPPNTKFVYVRKVFPDPSGSFTLFRLSNLRSQVVC-NTLMDIRWQSDRRNNVG        1238
TcCLB.510285.70 HQRDKSSVPKDTKFVYVRKVFPDPSGSFTLFRLSNLRSQVVC-NTLMDIRWQSDRRHNVG        1255
 ::*. ** .***.*:**.****:***.************ *:::*******:.:***

LbrM.35.5600 QKYYINADGTAEPFLVDQTGILSQLETVLNNSFRR        1255
LtaP36.5470 QKYYINADGTAEPFLVDNTGILSQLETVLNNNFRR        1254
LmxM.36.5350 QKYYINADGTAEPFLVDQTGILSQLETVLNNNFRR        1255
LmjF.36.5350 QKYYINADGTAEPFLVDQTGILSQLETVLNNNFRR        1255
LINF_360063000 QKYYINADGTAEPFLVDQTGILSQLETVLNNNFRR        1255
LdBPK.36.2.005580 QKYYINADGTAEPFLVDQTGILSQLETVLNNNFRR        1255
Tb927.11.10520 QKYYVRPNGEAGPFTAEHSGILNHVNLVMRNVYRK        1260
TvY486_1111400 QKYYVLADGTTEPFVVDHTGILSQVERVLCNNFRR        1273
TcCLB.510285.70 QKYYVLADGTAEPFVVDHTGILTQVETVLSNNFRR        1290
 ****: :* : ** .:::***.::: *: * :*:

# **KKT3**

LbrM.34.4040 -MLGSVDAIDYDGDRVRKVVLRFPALRSGDSESVKEVWPCERIGQGSFGTVYRAVSADYP        59
LtaP35.4080 MMLGTVDAIDYDGDRLHKVVLRFPAVRSGESETLKEVWPCERIGQGSFGTVYRAVSSDYP        60
LmjF.35.4050 -MLGTVDAIDYDGDRLHKVVLRFPAVRSGESEIVKEVWPCERIGQGSFGTVYRAVSSGYP        59
LmxM.34.4050 -MLGTVDAIDYDGDRLHKVVLRFPAVRSGDSEIVKEVWPCERIGQGSFGTVYRAVSSDYP        59
LINF_350046100 -MLGTVDAIDYDGDRLHKVVLRFPAVRSGESEIVKEVWPCERIGQGSFGTVYRAVSSDYP        59
LdBPK.35.2.004110 -MLGTVDAIDYDGDRLHKVVLRFPAVRSGESEIVKEVWPCERIGQGSFGTVYRAVSSDYP        59
TvY486_0904950 -MLGTVEKITHDEGLVRLVEVRLPFRGSSTG-QLLSLHLFERLGQGSFGSVYRACCDGYP        58
Tb927.9.10920 -MIGTVEQILYEEGCVRAVRVRLPSRDSSQG-KCLSLEACERIGQGSFGTVYRASCDEYP        58
TcCLB.508461.230 -MLGSVETISYDGEFVRLVELRLPLRGSTQG-QLLSIRTCERIGQGSFGTVYRAVCDGYP        58
 *:*:*: * :: :: * :*:* * . .: **:******:**** . **

LbrM.34.4040 RLALKISTGKGMRLRQELDVLSRVCTKGRLLLPRFEFGALNKTADLIVIGMELCVPSTLH        119
LtaP35.4080 RLALKISTGKSTRLRQELDVLSRVCTKGRLLLPRFEFGALNKTADLIVIGMELCVPSTLH        120
LmjF.35.4050 RLALKISTGKSTRLRQELDVLSRVCTKGRLLLPRFEFGALNKTADLIVIGMELCVPSTLH        119
LmxM.34.4050 RLALKISTGKSTRLRQELDVLSRVCTKGRLLLPRFEFGALNKTADLIVIGMELCVPSTLH        119
LINF_350046100 RLALKISTGKSTRLRQELDVLSRVCTKGRLLLPRFEFGALNKTADLIVIGMELCVPSTLH        119
LdBPK.35.2.004110 RLALKISTGKSTRLRQELDVLSRVCTKGRLLLPRFEFGALNKTADLIVIGMELCVPSTLH        119
TvY486_0904950 HLALKIATGKSARLREELAVLSKVCTKGKLLLPRFEFGAINKAGDLIAVGMELCVPCTLH        118
Tb927.9.10920 RLALKITTGKVTRLKQELEVLGRVCTKGKLLLPRFLFGALNKSGDLMAVGMELCFPHTLH        118
TcCLB.508461.230 KLALKIATGKAARLRQELDVLVKVCTKGKLLLPRFEFGAINKTGDLIVVGMELCVPSTLH        118
 :*****:*** **::** ** :*****:****** ***:**:.**:.:*****.* ***

LbrM.34.4040 DLLLSTRITSEAEMLFMAHQAVQAVSYVHMEGCIHRDVKLQNFVFDLDGNLKLIDFGLAC        179
LtaP35.4080 DLLLSTRITSEAEMLFMAHQVVEAVSYVHAEGCIHRDIKLQNFVFDLDGNLKLIDFGLAC        180
LmjF.35.4050 DLLLSTRITSEAEMLFMAHQAAQAVAYVHAEGCIHRDIKLQNFVFDLDGNLKLIDFGLAC        179
LmxM.34.4050 DLLLSTRITSEAEMLFMAHQAVQAVSYVHAEGCIHRDIKLQNFVFDLDGNLKLIDFGLAC        179
LINF_350046100 DLLLSTRITSEAEMLFMAHQAVQAVSYVHAEGCIHRDIKLQNFVFDLDGNLKLIDFGLAC        179
LdBPK.35.2.004110 DLLLSTRITSEAEMLFMAHQAVQAVSYVHAEGCIHRDIKLQNFVFDLDGNLKLIDFGLAC        179
TvY486_0904950 DVLLSTRLTNEADMLFIAHQVVQAVAYVHEHQCIHRDIKLQNFVFDLDGNLKLIDFGLAS        178
Tb927.9.10920 DFLLSKCLTDEADKLFVAYQVVQAVAYVHEQHCIHRDVKLQNFVFDLDGNLKLIDFGLAT        178
TcCLB.508461.230 DLLLVTRLTNEADMLFLAYQVLQAVACVHEQQCIHRDVKLQNFVFDLDGNLKLIDFGLAS        178
 *.** . :*.**: **:*:*. :**: ** . *****:*********************

LbrM.34.4040 NSLKPPAGDVVAGTVSFMSPEMAHNALHKDKRVSVGVAADVWSLGIVLFSIFTQRNPYPS        239
LtaP35.4080 NSLKPPAGDVVAGTVSFMSPEMAHNALHKDRRVSVGVAADVWSLGIVLFSIFTQRNPYPS        240
LmjF.35.4050 NSLKPPAGDVVAGTVSFMSPEMAHNALHKDRRVSVGVAADVWSLGIVLFSIFTQRNPYPA        239
LmxM.34.4050 NSLKPPAGDVVAGTVSFMSPEMAHNALHKDRRVSVGVAADVWSLGIVLFSIFTQRNPYPA        239
LINF_350046100 NSLKPPAGDVVAGTVSFMSPEMAHNALHKDRRVSVGVAADVWSLGIVLFSIFTQRNPYPA        239
LdBPK.35.2.004110 NSLKPPAGDVVAGTVSFMSPEMAHNALHKDRRVSVGVAADVWSLGIVLFSIFTQRNPYPA        239
TvY486_0904950 TNWNPPAGDVVAGTVSFMAPEMAHNALYRDKRVSVGAPADVWSVGIVLYSIFMQRNPYPP        238
Tb927.9.10920 SVWNPPPGDVVAGTIAFMAPEMAHNALHRDQRVSVGAAADVWSVGMVLFSIFAQRNPYAS        238
TcCLB.508461.230 SAWNPPPGDVVAGTVSFMAPEMAHNALHRDQRVSVGAPADVWSAGIVLFSIFTQRNPYPP        238
 . :** *******::**:********::*:*****. ***** *:**:*** *****

LbrM.34.4040 METPAQAADGFSSGTKNGGAAGTAAAGVSGPGETMSGVEGARGNSVSWQQRMNERLLRRV        299
LtaP35.4080 QETPAQAAGGAS--------GGTCAAGVAGPVDAPNGVEGEKGNGLSQQHRMNERLLRRV        292
LmjF.35.4050 P-----AAGSTP--------GGADAAGVTGHGDTTHGAEGEKGNGLSQQHRMNERLLRRV        286
LmxM.34.4050 PASPAPVAGSTS--------GGASAAGLTGHGDTTHGAEGEKGNSLNQQHRMNERLLRRV        291
LINF_350046100 PETPAPAAGSTP--------GGAGAAGVTGRGDITHGAEGEKGNDLSQQHRMNERLLRRV        291
LdBPK.35.2.004110 PETPAPAAGSAP--------GGAGAAGVTGRGDITHGAEGEKGNDLSQQHRMNERLLRRV        291
TvY486_0904950 VTATGYTSCTRHTGKSEEAVSGAAA--------NHN---NNATGVSNSLAKENVELLHRV        287
Tb927.9.10920 TGAPRMTERTADTN---MHNTAAIP--------TDA---SPNDKQSHEIPKENAELLRRV        284
TcCLB.508461.230 SIASITSPHSDEDE-------------------DGD---GDGAAVARKLNRENLRLLRRV        276
 . : * .**:**

LbrM.34.4040 AAGDWHWPVGVTVSQDLKQLVNSILVTNPEERPSVETILANKVWNLRRRYPPAAVAAFLG        359
LtaP35.4080 AAGDWQWPVGVTVSQDLKQLVNSILVVNPEERPSVSTILANKLWNLRRRYPPAAVAAFLG        352
LmjF.35.4050 AAGDWQWPVGVTVSQDLKQLVNSILVVNPEERPHVNTILENKLWNLRRRYPPAAVAAFLG        346
LmxM.34.4050 AAGDWQWPVGVTVSQDLKQLVNSILVVSPEGRPSVSTILENKLWNLRRRYPPAAVAAFLG        351
LINF_350046100 AAGDWQWPVGVTVSQDLKQLVNSILVVNPEERPSVSTILENKLWNLRRRYPPAAVAAFLG        351
LdBPK.35.2.004110 AAGDWQWPVGVTVSQDLKQLVNSILVVNPEERPSVSTILENKLWNLRRRYPPAAVAAFLG        351
TvY486_0904950 AAGEWR-----------------------------------TEWCSRRRSPPTAITVYLG        312
Tb927.9.10920 AAAEWSWPSGCSVSRKLRGLVDFVLVPDPQNRPDINALLMRPEWGDRRRATPRVVTTFLG        344
TcCLB.508461.230 ATAKWRWPVGSSVSNELRQLVEFILVTDPVKRPDISTILRKPIWNLRRRAPPTAVTAFLG        336
 *:..* * *** * .::.:**

LbrM.34.4040 VQDDFLLSHDEAHLMRAVEERSAGVTASLLNSRLHSPASTSSEGNGEANPQHSNNDSVRS        419
LtaP35.4080 VQDDFLLSHDEAHLMRAVEERSAGVAASLLNSRLHSPASSSSEDNGETDAQHSSSGSVRN        412
LmjF.35.4050 VQDDFLLSHDEAHLLRAVEERSAGVTASLLNSRLHSPTSASNEDNSETDAQHSSSGSARD        406
LmxM.34.4050 VQDDFLLSHDEAHLMRAVEERSAGVTASLLNSRLHSPASASSEDNGETDAQHSSSGSARN        411
LINF_350046100 VQDDFLLSHDEAHLMRAVEERSAGVAASLRNSRLHSPASASNEDNGETDAQHSSSGSARN        411
LdBPK.35.2.004110 VQDDFLLSHDEAHLMRAVEERSAGVAASLRNSRLHSPASASNEDNGETDAQHSSSGSARN        411
TvY486_0904950 VHDDFLLSHDEAHLLRAVKQRSANVTASLMEGHVDVLTGDET------------------        354
Tb927.9.10920 VEDDFLLSHDESHLLRAVEQRSADVNASLTESRIRGSDEEET------------------        386
TcCLB.508461.230 VQDELLLSHDETHLLRAVEQRSADVTASLMGSRANSLDGDGDE-----------------        379
 *.*::******:**:***::***.* *** .:



LbrM.34.4040 GGPRTPDTTTPPARGSLKVVQRCGEGGVDGAVAVQVYDVRASTRKRGKPIREISVVMAAE        479
LtaP35.4080 GGLHTSGATTSPARSSLKVVQRCGEAGIDGAVTVQVYDVRASTRKRSKPIREISVVMAEE        472
LmjF.35.4050 GGLHASGTTTSSPRGSLKVVQRSGEGGIDGAVTVQVYDVRASARKRSKPIREISVVMAEE        466
LmxM.34.4050 VGLHTSGATTSPARNSLKVVQRCGEGGIDGAVTVQVYDVRASTRKRSKPIREISVVMAEE        471
LINF_350046100 GGLNTSGATTSPARSSLKVVQRCGEGGIDGAVTVQVYDVRASTRKRSKPIREISVVMAEE        471
LdBPK.35.2.004110 GGLNTSGATTSPARSSLKVVQRCGEGGIDGAVTVQVYDVRASTRKRSKPIREISVVMAEE        471
TvY486_0904950 ---------EEGRRSSLKFVAK-EELTVGGVPTHQVYDVRGER-RGKKPIRELSVVISEE        403
Tb927.9.10920 ---------EEGHRSSLKFVAR-EELSVGGVPIHQVYDVRPDP-KVKKPIREISSVIAEE        435
TcCLB.508461.230 ---------EAERRSSLKFVAR-EQTAVGGVPTHQVYDIRRES-RQKRPLREISVVIAEE        428
 *.***.* : : :.*. ****:* . : :*:**:* *:: *



LbrM.34.4040 TAKTRR---SKSARRAKGVVSAPSSCGASRAASAEGDRRTASLAGTALQPSATHTCINSG        536
LtaP35.4080 TAKTRR---SKSTRRAKSAMSAASSCVGSRAASAEHDRRIAPSLGSRLQSSAAHMCANTG        529
LmjF.35.4050 TAKTRR---SKSARRAKSAVSAPSSRVGSRAASAENVRRIAPPAGTRLQSSSAHACADSG        523
LmxM.34.4050 TAKMRR---SKSARRAKSAVSAPTSRVSSRAASAENGRRRAPLAGARLESSAAHTCVNSD        528
LINF_350046100 TAKTRR---SKSARRATGAVSAPSSRVVSRAASTEYSRRIAPPAGARLQSSAAHTCANSG        528
LdBPK.35.2.004110 TAKTRR---SKSARRATGAVSAPSSRVVSRAASTEYSRRIAPPAGARLQSSAAHTCANSG        528
TvY486_0904950 IANSQRHKRSKSARDGSASTSRATSRALSRARGAVVDPSRAELSPVRP--LISTPHV--A        459
Tb927.9.10920 TERSLKRSRSRSARAAASSASRANSRVNARSR---------TVTP--------VPCT---        475
TcCLB.508461.230 TAKGLRRRQPKRARGDVSSVSRGNSCAGSRINSHANSR---ATSPARQ--CSVHECVQSL        483
 . : : :* . * .* :*

LbrM.34.4040 DDEDE----GEVMNRGASPSKHHNRGVLPVRLQDTLDTADTTAAEQRDNLWCSKTSVESS        592
LtaP35.4080 DDDCEAG--AEAGNSGTSTLQHHSRGVSPVRLQDALDTADSVAAGQADSVCHPKALVEPS        587
LmjF.35.4050 DDEGE----AEAVNRGACTSQRHSRGMSPVRLQDALETAGSVAAGQRDSVGHPKAWVEPS        579
LmxM.34.4050 DDEGEAE--AEVVNRGASTSQHHSRGVSPVRLQDALDTAGSVAAGQGDSVGHPTVLVEPS        586
LINF_350046100 DDEGEAE--AEAVNRGTSTSQRHSRGMSPVRLQDALETAGSVAAGQRDSVGHPKALVEPS        586
LdBPK.35.2.004110 DDEGEAEAEAEAVNRGTSTSQRHSRGMSPVRLQDALETAGSVAAGQRDSVGHPKALVEPS        588
TvY486_0904950 CDAGGAATFSSGPTTEECTLRNH-GGELL--LVGDVAVE---GGGREDAV----------        503
Tb927.9.10920 TEGRGNADFPTGSAAASLVLLEA-EGKEE--TKTGLNI--------GQRS----------        514
TcCLB.508461.230 VKGKKNGKMSRTIPQASSVIFDD-EEEEG--VQREV----------EQMV----------        520
 . : :



LbrM.34.4040 CATPPLFASD--------------------QIRPGLQRSGSVELLEDAEAPTVVRVKPSS        632
LtaP35.4080 CGTPLVLEGDKPQPVIGSASCTSTVGRANEEMRSSVQRSGSVELLECSAAPTAASAMSTS        647
LmjF.35.4050 CATPPLLPRDKQQPVSEPASSASTVDRAREHMRPGIQRSGSVELLKDAEAPTEASAMPTS        639
LmxM.34.4050 CATPPLLTRDTQQPVSEPASRGSTASREREHMRSGIQRSGSVELLEDAEAPTEANAMSTS        646
LINF_350046100 CATPPLLTSGKQQPLS-------------EHMRPDIQRSGSVELLEDAEAPTEASAMPTS        633
LdBPK.35.2.004110 CATPPLLTSGKQQPLS-------------EHMRPDIQRSGSVELLEDAEAPTEASAMPTS        635
TvY486_0904950 ------VT--ENQPRSRS---------CGEVDIN----SGTVDERVCGSLPT-PREVMNL        541
Tb927.9.10920 ------PQ--GRQPRSNNVDRAKGINLVGKRDPKPKSANGTVQRS--------TSPIEHT        558
TcCLB.508461.230 --------------------TKKEKPFVATQRIQKKQRNGGVCSHQCLRSSRIGDEAENT        560
 .* *

LbrM.34.4040 HNRAASGSRRKRDTSLRQAFSPTLKGGLRGTSGGVPGSTGTTTSAQVLVSQLTSRTASPP        692
LtaP35.4080 QKRFAPGGKKRRDTSLRQPSSSILKGGICDLSTAAPNNKTTAASVQVSTSLLASRIESPL        707
LmjF.35.4050 HKRAASGGKRSRNASLRQPSSLILKGGTRDLSSDAPDSTATTASAQVPASLLASRTTSSL        699
LmxM.34.4050 HNRAASGGKKRRDTSLRQPSSLILKGGIRDLSADAPRSTATTASARVSASLLASRITSPL        706
LINF_350046100 HKRAASSGKKRRDASLRQPSSLILKGSTRDLSADAPRSTTTAASTQASTSLLASRTTLPL        693
LdBPK.35.2.004110 HKRAASSGKKRRDASLRQPSSLILKGSTRDLSADAPRSTTTAASTQASTSLLASRTTLPL        695
TvY486_0904950 ATEYRAKGRSKSSVRSRSA-----------------------------------------        560
Tb927.9.10920 VDTKRGGGNNSHGVGARRGSTVT----------------------------------ARK        584
TcCLB.508461.230 KGLLRGG-INGSAVDISSGAVLE----------------------------------LPK        585
 .



LbrM.34.4040 SAMNPSPSSSRQASLRRQGSSSATTVSSAPGRAGHKGSSLAMKR--AQRVALELSLNVIG        750
LtaP35.4080 STGDPSPSSSRRASLKRQTPAGTAAISSAQERVGHKGSLPVMKR--ARRVALELELDVIW        765
LmjF.35.4050 SAVNPSPSSSRQALLRRQASASVAAVSSAQGRAGHRGSPPVMKR--AQRVALELGLDVIW        757
LmxM.34.4050 SAVNPSPSSSRQGSLRRQASASAAAISSAQWRAGHKGSPPVMKR--AQRVALELGLDVIW        764
LINF_350046100 SAVNPSPSSSRQASLRRQASASAAAVSSAQGCAGHRGSSPVMKR--AQRVALELGLDVIW        751
LdBPK.35.2.004110 SAVNPSPSSSRQASLRRQASASVAAVSSAQGCAGHRGSSPVMKR--AQRVALELGLDVIW        753
TvY486_0904950 -----------------------------SGPTTHKVSASHI----PQAPEVIFYVLETV        587
Tb927.9.10920 -----------------------------------K------KGNGAEV------VSGFV        597
TcCLB.508461.230 CQM----------GL----------TKAVDGETRTKKVPIHVHCDRKEQVSVWIDDMQLV        625
 : .

LbrM.34.4040 QDEADHRRTLSAMLLIEHAWLLASFRLTIEEDQERYSITWLAEEQEKSAAHPHRFKEVLR        810
LtaP35.4080 QDEADQRRALSAMLLIEHGWLLASFRLTIQEDQERYSITWLAEEQEKSAAHPHRFKEVMQ        825
LmjF.35.4050 QDEADHRRALSAMLLVEHAWLLASFRLTIEEDQERYSITWLAEEQEKSAAHPHRFKEVMQ        817
LmxM.34.4050 QDEADHRRTLSAMLLIEHAWLLASFRLTIEEDQERYNITWLAEEQEKSAAHPHRFKEVMQ        824
LINF_350046100 HDEADHRRALSAMLLIEHAWLLASFRLTIEEDQERYSITWLAEEQEKSAAHPHRFKEVMQ        811
LdBPK.35.2.004110 HDEADHRRALSAMLLIEHAWLLASFRLTIEEDQERYSITWLAEEQEKSAAHPHRFKEVMQ        813
TvY486_0904950 KTESDIREVVESLLLTQHECVVRGIQLVMEEMTERGNMQWLEKEQRKSAPHPHRFKGTLG        647
Tb927.9.10920 TMENAVRNVKTSLLLLEHSCMVTRLSTVVTELFDREHMVWLEKEQRKSATHPHTFREMGR        657
TcCLB.508461.230 AMEKSQRELAEKRIFLELECLFSALRLTTEENQAWFDMVWLAEEQRKSAAHPHRFKETTR        685
 * *. :: : :. : . * : ** :**.*** *** *:

LbrM.34.4040 VMGKKYQYGFVCDLCDYEFLPAGPEEKDLHFFHCPCGRDLCPDCYTAYQQQCTCSCCRVV        870
LtaP35.4080 VMSKKYQYGFVCDMCDYEFLPSGPEEKDLHFFHCPCGRDLCPDCYTAYQRQCTCSCCRTV        885
LmjF.35.4050 VMSKKYQYGFVCDMCDYEFLPTGPGEKNLHFFHCPCGRDLCPDCYTTYQRQCTCSCCRAV        877
LmxM.34.4050 VISKKYQYGFVCDMCDYEFLPTGPGEKDLHFFHCPCGRDLCPDCYTAYQRQCTCSCCRVV        884
LINF_350046100 VMSKKYQYGFVCDMCDYEFLPTGPGEKDLHFFHCPCGRDLCPDCYTAYQRQCTCSCCRAV        871
LdBPK.35.2.004110 VMSKKYQYGFVCDMCDYEFLPTGPGEKDLHFFHCPCGRDLCPDCYTAYQRQCTCSCCRAV        873
TvY486_0904950 S-VKKYRYGFVCDMCDYDYLPDG---STLYFFHCTCGRDMCVDCHKTYAESCRCRVCGQI        703
Tb927.9.10920 A-NKKYRYGFVCDVCCFEFEPVG---SSMYFFHCQCGRDMCPKCYEEYANNYTCDACGRE        713
TcCLB.508461.230 V-SKKYQYGFVCDMCDYEFLPAG---KTIHFFHCTCGRDLCVDCHRVYSKQCTCAECGAI        741
 ***:******:* ::: * * . ::**** ****:* .*: * .. * *

LbrM.34.4040 HPNSCVLREHLFRTGGTQYYIGSRTGSAVAQAGGVRCSFQAAVKHDEEKEGRDETSVPPE        930
LtaP35.4080 HSNSCVLREHLLQTGGSEYYSASREANGVTRADAVRGSAQATASLNKETASRDEASAPPQ        945
LmjF.35.4050 HSNSCVLREHLLRTGGGQYYSGSRKTNAAARADAVCGSFQAAAILDEEAESGDEASAPPE        937
LmxM.34.4050 HSNSCVLREHLLRTGGTQYYSGSRKMNAAARADAVRGSFQAASSLNEETASGHEASAPPE        944
LINF_350046100 HSNSCVLREHLLLTGGTQYYSGSRKTNAAARADAVRGSFQAAASLNEEAESGDEASAPPE        931
LdBPK.35.2.004110 HSNSCVLREHLLLTGGTQYYSGSRKTNAAARADAVRGSFQAAASLNEEAESGDEASAAPE        933
TvY486_0904950 LPNKISLREHQASKNCVESSTPPSKA--------PGASANQRSLMAGS-------KRPR-        747
Tb927.9.10920 FASSGALRRHS----CSCVKRLNDAATV----NKPRGRSQG-R---------RSRSVPTE        755
TcCLB.508461.230 YENSVALRGHHRKGGCKAVVKNRRSSVA----YEGQVSANG-PLMRRKT-AAQTHDMPRG        795
 .. ** * : .

LbrM.34.4040 LPRRRGRPPKQDKNHSPVKQKGNRAAKDTNHRRRGTQAAVDVSVGEVDEMAQLHLPRISI        990
LtaP35.4080 PPRRRGRPPKQDKNCAAM-KKGSRAAKDSSRRRRDAQDTLDVTVNEAHEVAQINPPRISI        1004
LmjF.35.4050 LPRRRGRPPKQDKNRSAVKEKGSRAAKDSSRRRRGAQDTLDVNVDEAHEVAQINPPRISI        997
LmxM.34.4050 LPRRRGRPPKQDKTRSAVKQMGSRAAKDSSRRRRGAQDTLDVSVNEVHEVAQINPPRISI        1004
LINF_350046100 PPRRRGRPPKQDKNRSAVKQKGSRAAKDSSRRRRGAQDTLDVSVDDAHEVEQINLPRISI        991
LdBPK.35.2.004110 PPRRRGRPPKQDKNRSAVKQKGSRAAKDSSRRRRGAQDTLDVSVDDAHELEQINLPRISI        993
TvY486_0904950 ---------R-------VTPKRVSAANDRKRKAA-AMP---------------AAVPVSA        775
Tb927.9.10920 A-ARTGVKSR-------LEVKPVSPKRGRPKRRA-SVPALERK-----RSREPAAEVVGI        801
TcCLB.508461.230 ATVATGSKA---------TTKAAAAA-------K-SFPRS-------------EAVRMGA        825
 : :.



LbrM.34.4040 AAMQQQEKRG-------RGAGAAGKVAPPHRPEDVVVKQRPVESVPEGPWRPFARYKKDR        1043
LtaP35.4080 AAMQQQEERGGNGLHRGGEAAVIGAEAAPQRPEDMEVKQRPVESVPEGPWRPFARFKRDH        1064
LmjF.35.4050 AAMQQKEERSSNGSHRGGGTAAVGVAARPQRPEDVEVKQRPVESVPEGPWQPFARFKKDR        1057
LmxM.34.4050 AAMQQQEDRSRNGSHRGRGTADVGAAAPPQRPEDVEVKQRPVESVPEGPWRPFARFKKDR        1064
LINF_350046100 AAMQQQEERSSNGSHRGGGTAAVGVAPRPQRPEDVEVKQRPVESVPEGPWRPFARFKKDR        1051
LdBPK.35.2.004110 AAMQQQEERSSNGSHRGGGTAAVGVAPRPQRPEDVEVKQRPVESVPEGPWRPFARFKKDR        1053
TvY486_0904950 TRK--------------RSTQAGGRNQ--LENETPS------------------------        795
Tb927.9.10920 VAR--------------RKAERLSMSK--SEVVPPPRRFEGIPRTLSGEWKPMERLSVGS        845
TcCLB.508461.230 VNT--------------VKKEAVAKEK--T---ASSLFSRPINTPPEGQWKPMERLSVSH        866
 . .



LbrM.34.4040 RDEEARQPTPEERDALLNGEWIRHFYLFPHAEPEHDGASGTRADEEEEEPYAFVYHAQPG        1103
LtaP35.4080 RNEVAGQPTPEERDALLNGEWIRHFYLFPHTESDSGAASGTWTE-GEEEPYAFVYHAQPG        1123
LmjF.35.4050 RDEVARQPTPEERDALLNGEWIRHFYLFPHAESERDAASGTCAE-GEEEPYAFVYHAQPG        1116
LmxM.34.4050 RDEVARQPTPEERDALLNGEWIRHFYLFPHAEREHGAASGTWAE-GEEEPYAFVYHAQPG        1123
LINF_350046100 RDEVAQQPTPEERDALLNGEWIRHFYLFPQAEPERVAASGTWAE-GEEEPYAFVYHAQPG        1110
LdBPK.35.2.004110 RDEVAQQPTPEERDALLNGEWIRHFYLFPQAEPERVAASGTWAE-GEEEPYAFVYHAQPG        1112
TvY486_0904950 ------------------------------------------------------------        795
Tb927.9.10920 R---SVPPTPEERHMLLNGDWIRYYHFYPMEE-------------EGGDSVAVTYHIQPG        889
TcCLB.508461.230 K---PIHPTPEEREVLLNGDWIRHFHLFPTEE-------------E-AEPVAFTYHIQPG        909

LbrM.34.4040 RTGAIFLTSDFSMHSAVFSMLERQFFVVNQVDTLEGADSTHATSLLKAKAHPELRIAFHA        1163
LtaP35.4080 RTGAIFLTSDFPMHSAIFSMLERQFFVVNQVDTVEGADSTRATSLLKAKGHPELRIAFHA        1183
LmjF.35.4050 RTGAIFLTSDFPMHSAVFSMLERQFFVVNQVDTVEGADSTCATSLLKAKGHPELRIAFHA        1176
LmxM.34.4050 RTGAIFLTSDFPMHSAVFSMLERQFFVVNQVDTVEGADSTRATSLLKAKGHPELRIAFHA        1183
LINF_350046100 RTGAIFLTSDFPMHSAVFSMLERQFFVVNQVDTVEGVDSTRATSLLKAKGHPELRIAFHA        1170
LdBPK.35.2.004110 RTGAIFLTSDFPMHSAVFSMLERQFFVVNQVDTVEGVDSTRATSLLKAKGHPELRIAFHA        1172
TvY486_0904950 ------------------------------------------------------------        795
Tb927.9.10920 RTGVTFFNHSFSVHSAVLSVLEHIVYVVDRVDIEEDNDVARILSLAQALNE--EKKIYDV        947
TcCLB.508461.230 RTGAIFLNHKFSMHSAVISVLERQMLIVDYVDTFENSDAARVVSLGNAKCL--AESAYNL        967

LbrM.34.4040 LQDIVAYDTNMMKQQRAPGTVSVYQAPRSAYSCNGEPFLYVRWFRFNENRTLSAFLLSNG        1223
LtaP35.4080 LQDIVAYDTNMMKQQRTPGTVSVYQAPRSAYSCNGEAFLYVRWFRFNENRTLSAFLLSNG        1243
LmjF.35.4050 LQDIVAYDTNMMKQQRTPGTVSVYQAPRSAYSCNGEPFLYVRWFRFNENRTLSAFLLSNG        1236
LmxM.34.4050 LQDIVAYDTNMMKQQRTPGTVSVYQAPRSAYSCNGEPFLYVRWFRFNENRTLSAFLLSNG        1243
LINF_350046100 LQDIVAYDTNMMKQQRTPGTVSVYQAPRSAYSCNGEPFLYVRWFRFNENRTLSAFLLSNG        1230
LdBPK.35.2.004110 LQDIVAYDTNMMKQQRTPGTVSVYQAPRSAYSCNGEPFLYVRWFRFNENRTLSAFLLSNG        1232
TvY486_0904950 ------------------------------------------------------------        795
Tb927.9.10920 LQLVETHDTHMLKQRRSPGIMSVYCPPQTAFQCNGDPFVFVRWYRFHMENSMSGFMLSNG        1007
TcCLB.508461.230 LQRVVAYDCNMMKQHRTPGTISIYRTAQTAIRCQGEPFVYVRWFRFDAERSLCAFLLSNG        1027

LbrM.34.4040 AVQVFVNNEYELRWFDESRKFLIRYNGVCELVDDGTFALAPAINHLLYDSFDV        1276
LtaP35.4080 AVQVFVNNEYELRWFDESRKFLVRYNGVCELVDDGTFALAPAINHLLYDSFDA        1296
LmjF.35.4050 AVQVFVNNEYELRWFDESRKFLIRYNGVCELVDDGTFALAPGINHLLYDSFDA        1289
LmxM.34.4050 AVQVFVNNEYELRWFDESRKFLIRYNGVCELVDDGTFALAPGINHLLYDSFDA        1296
LINF_350046100 AVQVFVNNEYELRWFDESRKFLIRYNGVCELVDDGTFALAPGINHLLYDSFDA        1283
LdBPK.35.2.004110 AVQVFVNNEYELRWFDESRKFLIRYNGVCELVDDGTFALAPGINHLLYDSFDA        1285
TvY486_0904950 -----------------------------------------------------        795
Tb927.9.10920 AVQVFVGGKYELRWLDDNRKFIVRSNGVCEVLDEEKFPLSEELNQMLYGGV--        1058
TcCLB.508461.230 AVQVLVGDQYELRWFDENRKFLLRSNGVCEMVDDSTFALAPDVSRLLYDDF--        1078

# **KKT4**

 LbrM.10.0320 MNADAQELVRQLTEKPEVLESMQHLISLLRANPQQCPGTSNDGSRSNVETTRPER-----        55
LtaP10.0290 MSTDAQELVRQLTENPEVLEGMQHMISLLRANPPHSVGSKNGESHSSGEINRAER-----        55
LmxM.10.0300 MSTDAQELVRQLTENPEVLESMQHMISLLRANPPRISGSNNGGGLGNAETNGPER-----        55
LmjF.10.0300 MSTNAQELVRQLTENPEVLESMQHMISLLRANPPRISGSSNGGGLGNVETNGPDR-----        55
LdBPK.10.2.000320 MSTNAQELVRQLTENPEVLESMQHMISLLRANPPRISGSSNGGGLGSVETNGPER-----        55
LINF_100008400 MSTNAQELVRQLTENPEVLESMQHMISLLRANPPRISGSSNGGGLGSVETNGPER-----        55
Tb927.8.3680 MGDNPLSVLQQLASNPQLITQMQGILALLSPGHGDGATGA-----------RPGGVGLEQ        49
TcBrA4_0020080 MEPNVLSVLQQLTSNPQLVGQLQSLLTVLSNVEQDGTGGG-------AMAARPAATTLGG        53
TvY486_0803080 MEPNVLSVVQQLTSNPQLVTQLQSILALLTPTQS-GSGSS-------VQPDGEASQRMAA        52
 * : .:::**:.:*::: :* ::::*

LbrM.10.0320 --GAQQWG-----RPPRS-GCAANVDYGRHQTTNMRKLHSSDGAGRSANSPSA---SSLT        104
LtaP10.0290 --GAQQRV-----RPPRS-GYGTDVEYGHHQPINRRKLHSSDDTGNRANSLSA---LSLT        104
LmxM.10.0300 --GAPQCV-----RPPRR-GYGADVDCDHHQPTTRRKLRSSDGTAHSATSLSA---SSLT        104
LmjF.10.0300 --GAQQCV-----RPPRG-GYGTDVDYGHHEPTARRKLHSSDGTGHSATSLSA---SSLT        104
LdBPK.10.2.000320 --GAQQCV-----RPPRS-EYGTDADYGHHRPTTRRKLHSSDGTGHSATSLSA---SSLT        104
LINF_100008400 --GAQQCV-----RPPRS-EYGTDADYGHHRPTTRRKLHSSDGTGHSATSLSA---SSLT        104
Tb927.8.3680 --IAALCPQ------PRTLQNTATTDMGAASSLMAGGLNVEPPRQQ-TRWPSPV----AT        96
TcBrA4_0020080 IPSPALA------SVPSSVH----------STQKRQGMSAAPRSAS-TQTASAARHARLV        96
TvY486_0803080 MGSSSLCSSVATAAPPPSVKPHT-------SVSHRQHFDLGPHNGH-HRALSR----GFV        100
 * : * .



LbrM.10.0320 QETHSFYSDDRVYARSTVNGHNGATGGAASPTPSFATTGLRGAPQGPTAASRRGLRHSPL        164
LtaP10.0290 QETHSFYSDERVGAHNTVSGHNGATVGASSPTPTFAPTGSRATPQVVTAPSRYAPRPPSL        164
LmxM.10.0300 QEAHSFYGDDRVGARTTVSDHNGTTGGASSPTPSFVSTGSRAAPQVVTAASRHAPRRSSL        164
LmjF.10.0300 QEAHSFYSDDRVGARTTVSGHNGATGGASSPTPTFATTGSRAAPQVVTAASRLAPRRSSL        164
LdBPK.10.2.000320 QEAHSFYSDDRVGARTTVSGHNAATGGASSPTPTFVATGSRAAPQVVAAASRLAPRRSSL        164
LINF_100008400 QEAHSFYGDDRVGARTTVSGHNAATGGASSPTPTFVATGSRAAPQVVAAASRLAPRRSSL        164
Tb927.8.3680 ASESSNL-------QSRG------------------DSKSDVGKYGVVSVERY----ERL        127
TcBrA4_0020080 EANNSQL----------S------------------SGVSEATAYEGVSLQRY----EKL        124
TvY486_0803080 QRPSSSISSRHSTSRNPI------------------EEGNDLNNFDATLLDRY----EQL        138
 * . .* *



LbrM.10.0320 LSTPHEHRSVTAPDEQLMATATKLTDAQRRIAELEKELQHTTQRVDQLSDVVQRQKDELQ        224
LtaP10.0290 LSTPHEHRPATASEEQLRATTSKLTEAQRRIAELEKELQRTTQRVDQLSDVVQRQKDELQ        224
LmxM.10.0300 LPSPHEHRPTTAPDEQLMATANKLTEAQRRIAELEKELQRTTQRVDQLSDVVQRQKDELQ        224
LmjF.10.0300 LSAPREHRPATAPDEQLISAANKLTEAQRRIAELEKELQRTTQRVDELSNVVQRQKDELQ        224
LdBPK.10.2.000320 LSTPHEHRPATAPDEQLMATANRLTEAQRRIAELEKELQRTTKRVDQLSDVVQRQKDELQ        224
LINF_100008400 LSTPHEHRPATAPDEQLMATANRLTEAQRRIAELEKELQRTTQRVDQLSDVVQRQKDELQ        224
Tb927.8.3680 MA---RYKELEKQSHRRQGKRSEPVVDTQRVLDLEEEVARLKRTIGHLQGVVEEKESALE        184
TcBrA4_0020080 VK---EYRRLEEELEQKK---HEASDASQRVRQLERETTRLMRRVEQLVSAVEGQKQKLD        178
TvY486_0803080 VV---HCRNLEQQLETC-----DVTNARRRVTQLEEEVKALTQRVEQLTGIVEQQKRLIK        190
 : . : . :*: :**.* : : .* . *: :: :.



LbrM.10.0320 TAQDRHLLEMEELRHSYNAVIQRKDEVQGEALRQLLKSRQLMVSAAKYEAVVTAKKSQAQ        284
LtaP10.0290 DERDRHVLEMEETRNAYSAAINRKDEVQEEALRQLLKSRQLMVSAAKYEAVVATKKFHAQ        284
LmxM.10.0300 AAKDRHALEMEETRHAYNAVIHRKDEVQEEALRQLLKSRQLMVSAARYEAVVAAKKLHAQ        284
LmjF.10.0300 ATKDRHTLEMEETRHSYNAVIHRKDEVQEEALRQLLKSRQLMVSAAKYEAVVAAKKFPAQ        284
LdBPK.10.2.000320 ATKDRHALEMEETRHAYNAVIHRKDEVQEEALRQLLKSRQLMVSAAKYEAVVAAKKFHAQ        284
LINF_100008400 ATKDRHALEMEETRHAYNAVIHRKDEVQEEALRQLLKSRQLMVSAAKYEAVVAAKKFHAQ        284
Tb927.8.3680 KHATQHNLEVHEMKKNYELKIKSLTQTHEAAVRKLVSAQELVTAARNYQTAVCANNVGGG        244
TcBrA4_0020080 ETEAKHKLELAEIENRHELEIQSKMSSHEEALRRLMDARRLMAAAVQYEQTLKSPAAERQ        238
TvY486_0803080 DREGTHSLEIAEMKHQHEATIQKIAAEHDEVLRQVIHAQQLTAAAMHYSSVVKDDKGKCG        250
 * **: * .: :. *: : .:*::: ::.* .:* .*. .:

LbrM.10.0320 QLAKENNHGVDDGMGSIKVLAGMQTAYSDNECDTRPGLAQRQTSVNAQHSSVLGYRSSTA        344
LtaP10.0290 QLEKENNTGGDDGMGGAIGLAGKQVSLSANDRGTHAGLAVSQTSVNARHSPTLGYGSGTT        344
LmxM.10.0300 RLEKENNTGADDAMGSPKGLAGVQASANPNERGTHPGLAPSQTSVNARHSSTLGYGSGTT        344
LmjF.10.0300 QLEKENNTGADDGMGSAKGLAGVQASSNPNEHGTHPGLAPSQTSVNARHSSTLGHGSGAT        344
LdBPK.10.2.000320 QLEKENNTGADDGTGSAKGLAGVQASSNPNEHGTHPGLAPSQTSVNARHSSTLGHGSGAT        344
LINF_100008400 QLEKENNTGADDGTGSAKGLAGVQASSNPNEHGTHPGLAPSQTSVNARHSSTLGHGSGAT        344
Tb927.8.3680 NSVSTT-------SGQP--L--------SNTVNHTRGLTTTSSGSGPNQPYTLPHPDGNA        287
TcBrA4_0020080 VSTV----------ATP--I-------------NSRGLQSRPGSSNKQ-------QQQRP        266
TvY486_0803080 EGGA----------E--------------------NGNKTTLHSSNRS--------EARV        272
 * . . .



LbrM.10.0320 TKYSSALKRERQNDDEDVVTDVDLVVDAYEPGDARYGATTQQRPPAKRSTADSWRLQGST        404
LtaP10.0290 AKHSSALKRDRENGEEYCDDGAGAAIGAAEPGELRYRELAHQRPPVKRTTLDTSRLQGSA        404
LmxM.10.0300 AKYSSALKRDRQNDEGDLVDDAGVETGAHEPGEARYGEAAHHHPPVKRTTLDTSRLQGSA        404
LmjF.10.0300 AKYNRALKRDRQNDEEDFVDDAGVASGAHEPGEARYGEAAHQRPPMKRTTLDTSHLQGSA        404
LdBPK.10.2.000320 AKYNSALKRDRQNDEEDFVDGAGVATGAQEPGEARYGEATHQRPPMKRTALDTSHLQGSA        404
LINF_100008400 AKYNSALKRDRQNDEEDFVDGAGVATGAQEPGEARYGEATHQRPPMKRTALDTSHLQGSA        404
Tb927.8.3680 -----------------WMSATT-----SDDRSA--PVTTKNSHSVKRER----------        313
TcBrA4_0020080 -----------------WGNEKGAKREAEAEEKS--AFVSQHSVPMKRER----------        297
TvY486_0803080 -----------------LSAANG-------PSGK--RLVASNATSMKQTR----------        296
 : : *:

LbrM.10.0320 DRAVQGRRGVAATNAETSPAYITTPTLCGKTSTALVETRTQPRSARKRRTPRTPSLTNAD        464
LtaP10.0290 DSAVQGRREVAVSKVETSPAYITTPTSAGKGSTALVDTRTQSRSARKRRTPRTPSLTNAD        464
LmxM.10.0300 DRVVQGRRGVAATKAETSPAYITTPTPAGKASTALVGTRTQSSSARKRRTPRTPSRTNAE        464
LmjF.10.0300 DRAVQGRRGVAATKAETSPAYITTPTPAGKASTALVDPRTQSRSARKRRTPRTPSRTNAE        464
LdBPK.10.2.000320 DRAVEGRRGVVATKAETSPAYITTPTPVSKASTALVDPRTESRSARKRRTPRTPSRTNAE        464
LINF_100008400 DRAVEGRRGVVATKAETSPAYITTPTPVGKASTALVDPRTQSRSARKRRTPRTPSRTNAE        464
Tb927.8.3680 ----------------------------------EGTVSTTPTRPLKKRNPRTPSYTVAD        339
TcBrA4_0020080 ----------------------------------DGASRSLSAVYRKKRTPRTPSLTAAD        323
TvY486_0803080 ----------------------------------------------------PTSVSDAD        304
 * : *:

LbrM.10.0320 RLAGSVAG---NSARLQ----QRLPGTA--SLKIESPTPVASTAWTADRSLTSSHT----        511
LtaP10.0290 RLAGSVLE---NGIRRQ----QCLPGTA--SLKIESPTPVVSTAWTADRSLTSSRT----        511
LmxM.10.0300 RIAGSVAE---NRIRSQ----QRLPGTT--SLKIESPTPVVSTAWTADRSLTGSRT----        511
LmjF.10.0300 RLAGSTAE---NRIRSQ----QRLPGTT--SLKIESPTPVVSTAWTADRSLTGSRT----        511
LdBPK.10.2.000320 RLAGSMAE---NRIRSQ----QRLPGTA--SLKIESPTPVVSTAWTADRSLTGSCT----        511
LINF_100008400 RLAGSMAE---NRIRSQ----QRLPGTA--SLKIESPTPVVSTAWTADRSLTGSCT----        511
Tb927.8.3680 RISETDEYVKK--------------GSPVVKKELNSPTAEAQQTCLDGMGYQPAVGSNIL        385
TcBrA4_0020080 RV--L-ATTDTNSAATAAPHSKTKSHSAKVERELPHGPADTTAAFVED-RYLVKA-RNPS        378
TvY486_0803080 QFRVVKPTSDDNDVGVRWPKSV-KRERDEVGRRIPKKTYSAGSSWT--------------        349
 :. .: . :



LbrM.10.0320 -PPPHSSGGMNAASEAVIKPHHLSQ-QQSQQPSSTK-----------PPLTQRAAGRLPP        558
LtaP10.0290 -PPPSTA-GTCTVSEAVTKHHQLHPQQP---VPSTR-----------PPLAQRAAGRLPP        555
LmxM.10.0300 -PPPSSA-GVCTVSEAVTKHHQLYPQQQVHQVPSTR-----------PPLMQRAAGRLPP        558
LmjF.10.0300 -PPPSSP-GMHTVSEAVTKHHQLHPQQHVHQAPYTR-----------PPLMQRAAGRLPP        558
LdBPK.10.2.000320 -PPPSSA-GMYTVSEAVTKHHQLHPQQHVQQAPSTR-----------PPLMQRAAGRLPP        558
LINF_100008400 -PPPSSA-GMYTVSEAVTKHHQLHPQQHVQQAPSTR-----------PPLMQRAAGRLPP        558
Tb927.8.3680 RAQTSYTSAACTLVEAVSNSRQQ---FQQQRSESVQMQ----PRSGAVALRQRTGGLS--        436
TcBrA4_0020080 SSTVSPGPATSTITEAMARHHHQQKQQQQQRDATNNVESKFVAQYGAAVLPPAPSLPSPP        438
TvY486_0803080 -------AAHNTLRDVPSRHFDL-----AQDDANSEADAKA---------SAIATLPLPR        388
 . : :. . . .



LbrM.10.0320 AHH---------------------------RAAAAPTAVPNTRSGTSSIASGGPTRSPSP        591
LtaP10.0290 APH---------------------------RTAAARAAVPNTRSGTSSIASGGPTRSPSP        588
LmxM.10.0300 APH---------------------------RTAAASTAVPNTRSGTSSIASGGPTRSPSP        591
LmjF.10.0300 APH---------------------------RTVAAPTAVPNTRSGTSSIASGGPTRSPSP        591
LdBPK.10.2.000320 APH---------------------------RTAAAPTAVPNTRSGTSSIASGGPTRSPSP        591
LINF_100008400 APH---------------------------RTAAAPTAVPNTRSGTSSIASGGPTRSPSP        591
Tb927.8.3680 PVA--SSSGSAL-------MATPSSR--------GLRRPFRPVSGASS-AVGGSTRSPSP        478
TcBrA4_0020080 PLPLPTTTKTSL----ATASRTPRQRRPPHAPLQQQHSRSGQRSGTSS-VVGGPTRSPSP        493
TvY486_0803080 PLSSGTTPPRALLPTAGGLYRTPRLR----GQRLQLQGRGGMRSGTSS-VVGGPTRSPSP        443
 **:** . ** ******

LbrM.10.0320 VNPKRGSTLPRRFIFTGLKDHEPQRLASAIAAVGDDAAALASDLDEPPPSSTTHIVLRGT        651
LtaP10.0290 VNPKRGAVLPRRFIFTGLKDNEPQRLASAIAAIGDDAAALASDLDEPPPTSTTHIVLRGT        648
LmxM.10.0300 VNPKRGAMLPRRFIFTGLKDHEPQRLVSAIAAVGEDAAALASDLDEPPPSSTTHIVLRGT        651
LmjF.10.0300 VNPKRGAMLPRRFIFTGLKDHEPQRLVSAIAAVGDDAAALASDLDEPPPSSTTHIVLRGT        651
LdBPK.10.2.000320 VNPKRGAMLPRRFIFTGLKDHEPQRLVSAIAAVGDDAAALASDLDEPPPSSTTHVVLRGT        651
LINF_100008400 VNPKRGAMLPRRFIFTGLKDHEPQRLVSAIAAVGDDAAALASDLDEPPPSSTTHVVLRGT        651
Tb927.8.3680 VDPKRGAVQPRYFITTSLTEKERNSVMEAIQKLGQRAVLVDNKVDEILPLNTTHIVLRGP        538
TcBrA4_0020080 VNPKRGAEQPRCFVVTSLTEEERARVKAAVAAIGQHGVVLESDYEDPPPFTATHIVVRGP        553
TvY486_0803080 INPRRGAEQLRVFVLTSVSEEERERIKFAIEAIGQRASILDSSPDDLPPVTMTHIVLRGP        503
 ::*:**: * *: *.:.:.* : *: :*: . : .. :: * . **:*:**

LbrM.10.0320 PRSVKALCGVVSGKWLVSPEYVYNSQQSGFWLDELEEGGLRIFPPPLKCQRFLLTVEHPS        711
LtaP10.0290 PRSVKALCGVVSGKWLVSPEYVYNSHQSGFWLDELEEGGLRIFPPPLKCQRFLLTVEHPS        708
LmxM.10.0300 PRSVKALCGVVSGKWLVSPEYVYNSQQSGFWLDELEEGGLRIFPPPLKCQRFLLTVEHPG        711
LmjF.10.0300 PRSVKALCGVVSGKWLVSPEYVYNSQESGFWLDELEEGGLRIFPPPLKCQRFLLTVEHPS        711
LdBPK.10.2.000320 PRSVKALCGVVSGKWLVSPEYVYNSQQSGFWLDELEEGGLRIFPPPLKCQRFLLTVEHPS        711
LINF_100008400 PRSVKALCGVVSGKWLVSPEYVYNSQQSGFWLDELEEGGLRIFPPPLKCQRFLLTVEHPS        711
Tb927.8.3680 PRSVKALCGVVSSKWLVQPSYVFDSLGAGFWLDEEVEGGLRYFPPPLRCQRFLLTMPEGV        598
TcBrA4_0020080 PRSMKALCGVVGSKWLVQPEYIYASRDAGFWLDEYEEGGMRCFPPPLKCQRFLLTMPEGI        613
TvY486_0803080 PRSTKALCGVVAAKWLVQPEYVYASQEAGFWLDEYEEGGFRCFPPPLKCQRFLLTLPDNI        563
 *** *******..****.*.*:: * :****** ***:* *****:*******: .

LbrM.10.0320 IRAKLAQVIEYGGGEVLPSGSGKHGASAGSTVAQDVIVITSGDDLLRYATQDRV-        765
LtaP10.0290 IRAKLAQVIEYGGGEVLPSGSDQRGPGASGTVAQDVIVITSGDDLLRYATQDRV-        762
LmxM.10.0300 IRAKLAQVIEYGGGEVLASGSDKRGPGAGDTVAQDVVVITSGDDLLRYATQDRV-        765
LmjF.10.0300 IRAKLAQVIEYGGGEVLPSGSYKRGPSAGDTVAQDVIVITSGDDLLRYATQDRV-        765
LdBPK.10.2.000320 IRAKLAQVIEYGGGEVLPSGSDKRGPSAGDNVAQDVIVITSGDDLLRYATQDRV-        765
LINF_100008400 IRAKLAQVIEYGGGEVLPSGSDKRGPSAGDNVAQDVIVITSGDDLLRYATQDRV-        765
Tb927.8.3680 VKTMLQRVVEFGGGEVVGTKRNG------SSNDQDVVVVSSGDELLRFAISRD--        645
TcBrA4_0020080 VKEKLEQVIEYGGGEVVRPARDG------RSYDQGVVVIASGDDLLHFATRIE--        660
TvY486_0803080 VRDKLVQVIEYGGGEVIQKDKSR------RGHDQGVVVISSGDELLRFATRQGNL        612
 :: * :*:*:*****: *.*:*::***:**::*

# **KKT7**

Tb927.11.1030 -MSGYGESMHSLREPVSPCPRRRSLASTPLERQRSYSRGGMVGSPVPDDMSFTLSPLPYF        59
LbrM.27.0520 -MTDVASSHRPPSHQVSPVPRRQ-LGVLP-VNQRSYSRVGSKGM-IGDDSPL-MSPLPYY        55
LtaP27.0430 -MTEVTSSLRPPSRQGSPVPRRQ-LGILP-VNQRSYSRVGSKGM-IGDESPL-MSPLPYY        55
LmjF.27.0430 -MTDVTSSLRPSSRQGSPVPRRQ-LGILP-VSQRSYSRVGSKGM-IGDDSPL-MSPLPYY        55
LmxM.27.0430 -MTDVTSSLRPSSRQGSPVPRRQ-LGILP-VNQRSYSRVGSKGM-IGDDSPL-MSPLPYY        55
LINF_270009400 -MTDVTSSLRPSSRQGSPVPRRQ-LGILP-VNQRSYSRVGSKGM-IGDDSPL-MSPLPYY        55
LdBPK.27.2.000440 -MTDVTSSLRPSSRQGSPVPRRQ-LGILP-VNQRSYSRVGSKGM-IGDDSPL-MSPLPYY        55
TcCLB.506925.490 MTEVCGEETLVPSYPVSPRPRRQPMASLS-TNQRSYSRMSSKGI-FGDESPLLLSPLPKY        58
TvY486_1100920 -MSTHDKEVMVAGYTTSPRPRRQPLTPLL-RNQRSYSRLSSKGI-FGDDSPLLLSPLPRY        57
 .. ** ***: : ****** . * . *: : :**** :



Tb927.11.1030 PKRTRSVTFEESDEVPVREERPYYSWDDRHGLSDK---RG--------------------        96
LbrM.27.0520 PR-RRSVTFAGD--QSVREERPNYNVAYSASAPVSPVRQGTPPPISILKPKSSFPVIE--        110
LtaP27.0430 PR-RRSVTFAGD--QSVREERPNYNAAYSASAPISPARHGSPPPVSILKPNSSFPVAD--        110
LmjF.27.0430 PR-RRSVTFAGD--QSVREERPNYNAAYSTSAPVSPARRGSPPPVSILKLNSSFPAAE--        110
LmxM.27.0430 PR-RRSVTFAGD--QSVREERPNYNAAYSASAPVSPARHGSPPPVSILKSNLSFPAAE--        110
LINF_270009400 PR-RRSVTFAGD--QSVREERPNYNAAYSASAPVSPARHGSPPPVSILKPNSSFPAAE--        110
LdBPK.27.2.000440 PR-RRSVTFAGD--QSVREERPNYNAAYSASAPVSPARHGSPPPVSILKPNSSFPAAE--        110
TcCLB.506925.490 PNRRRSVTFADETEATVREERPYYTACETGNTPGK---HL-------SESPFRRPIIKTT        108
TvY486_1100920 PNRRRSVTFADEAETTVREERPYYTMNDLETPTKE---HE-------TYSSRKWQW----        103
 *. ***** . ****** *. . :



Tb927.11.1030 FDDNGAAVAVRDGGEECHYARSGAAGRREEGDQRYRPFVVAGTTFKIPRSRSQSRQHREA        156
LbrM.27.0520 AEDSGAAPAYHA-----------AAATVS-G-VFDRK-DPSRNSPVPARSRSTSRQRLAV        156
LtaP27.0430 EEDSGAAPAYQA-----------AAATVS-G-VFDHK-DFARNSPVPVRGRSNSRQRLAA        156
LmjF.27.0430 EEDSGAAPAYQA-----------AAATVS-G-VLDRQ-DRARNSPVPVRGRSNSRQRLAA        156
LmxM.27.0430 EEDSGAAPAYQA-----------AAATVS-G-VLDRK-DRARNSPVPVRGRSNSRQRLAA        156
LINF_270009400 EEDSGAAPAYQA-----------AAATVG-G-VLDRK-DRARNSPVPVRGRSNSRQRLAA        156
LdBPK.27.2.000440 EEDSGAAPAYQA-----------AAATVG-G-VLDRK-DRARNSPVPVRGRSNSRQRLAA        156
TcCLB.506925.490 SRTNNATDAVVA----------VNMNRHN-D-TTSHAPVAPATSPTIKRGRSSSRQRRAA        156
TvY486_1100920 --DNGAGKPLAE---------QGECPDSY-G-NTSRAPVSRPV--TPLRSRSVSRQRRIA        148
 ..* . : *.** ***: .

Tb927.11.1030 EEKLAMSTFSYPEIRKVEDFCTQLLRPLEENEPPQREGYRGAHTNEG--VNAGND-AGQP        213
LbrM.27.0520 RRKEAQLHRSFYDDSFVEEYVLHAKTELEQEEAEQRRVQEQLRAEQESAKRKERRVSEAT        216
LtaP27.0430 RRKEAQLHRSFYDDSFVEEYVLRAKTEQDEEEAEQRRLQEQLRVEQERAKRAERRVSEAT        216
LmjF.27.0430 RRKEAQLHRSFYDDSFVEEYVLRAKTELEQEEAEQRRIQEQLRAEQERAKRAERRTSEAT        216
LmxM.27.0430 RRKEAQLHRSFYDDSFVEEYVLRAKTELEEEEAEQRRMQEQLRAEQERAKRAERRVSEAT        216
LINF_270009400 RRKEAQLHRSFYDDSFVEEYVLRAKTELEQEEAEQRRMQEQLRTEQERAKRAERRVSEAA        216
LdBPK.27.2.000440 RRKEAQLHRSFYDDSFVEEYVLRAKTELEQEEAEQRRMQEQLRTEQERAKRAERRVSEAA        216
TcCLB.506925.490 RRHQAELYHGFYDDSIVEDYVLKAKKEIEGEE-EEKIVEENLKQQEKELAEAEMRAAQAT        215
TvY486_1100920 RRQEAQLHHSFYEDSMVEEYVLKVRKEIEEEE--QRMLEDRMRIQENEKREAEKRVVQAT        206
 ..: * .: : **:: : : :* :: : :: .

Tb927.11.1030 PEITPIRKAKATLKSGLKTDYVSRANCDLQRTVADHLPR--RRGRSNKREASRPKEEESG        271
LbrM.27.0520 EKINALQHAKEVLMAATVRRHPSVTPSPPR-----------APAEKAKRNSSLLREL-QE        264
LtaP27.0430 EKINALQHAKEVLMAATVRRHNSVTPSPQR-----------VPAEKSKRNSSLLREL-EE        264
LmjF.27.0430 EKINALQHAKEVLMAATVRRHTSVTPSPQR-----------APAEKSKRNSSLLREL-EE        264
LmxM.27.0430 EKINALQHAKEVLMAATVRRHTSVTPSPQR-----------APAEKSKRNSSLLREL-EE        264
LINF_270009400 EKINALQHAKEVLMTATVRRHTSVTPSPQR-----------APAEKSKRNSSLLREL-EE        264
LdBPK.27.2.000440 EKINALQHAKEVLMTATVRRHTSVTPSPQR-----------APAEKSKRNSSLLREL-EE        264
TcCLB.506925.490 VKISALQQAKEYLLATANARCFSPACSSSERRDSGFASS-RSCHHSLKKEPSLIQQ-ADG        273
TvY486_1100920 VKMNALQQAKEFLMSSARACCVSPVNAGSGASGSDEAPTAPVCGL--GKGASAARQSLDA        264
 ::. :::** * : * . . : * :: .

Tb927.11.1030 E-----EGYSMEASVAPHEAKQKTTRCVTTATPQCGVGVG-GASRRRTRCESVEEG--VV        323
LbrM.27.0520 D-----PDPEVQAAL----------------KELARNSVAKQQS--RAH-PSTYQRHRSI        300
LtaP27.0430 D-----PDPEVQAAL----------------KELARNSLAKQQS--RIH-SAVHQRRRSI        300
LmjF.27.0430 D-----PDPEVQAAL----------------KELARSSLAKQQS--RVH-SSAHQRRRSI        300
LmxM.27.0430 D-----PDPEVQAAL----------------KELARNSMAKQQS--RVH-SSAHQRRRSI        300
LINF_270009400 D-----PDPEVQAAL----------------KELARNSLAKQQR--RVH-SSAHQRRRSI        300
LdBPK.27.2.000440 D-----PDPEVQAAL----------------KELARNSLAKQQR--RVH-SSAHQRRRSI        300
TcCLB.506925.490 E--VEEVGDIEQQRR---HPPQKPVKHSMPLVTQMDDM-DEKGSGKRIRQVSLKKGGHSL        327
TvY486_1100920 SAAVVDEDSVVSIRM---R--PKTTSCSVPLVTMTEDGADTNESRKRVREKPQAVQESSL        319
 . . . * : :

Tb927.11.1030 HSTTQKCIHVIDIHNAEEEIKSLPTEITATVSCVVKGLLDDL-----SNQSFPIIIKPCK        378
LbrM.27.0520 SIVSADDL--AKSGEIDDD-GNNDTRKRARLEKIVSTLMAKRAQ-SKSKCSVMVIDWSDL        356
LtaP27.0430 SIVSADAL--AKGGEAENE-GDKDTRKRARLEKIVSTLLSKRAK-SKSKRSVMVIDWSDL        356
LmjF.27.0430 SIVSADAL--AKSGEAEDG-DDNDTRKRARLEKIVSTLLAKKAK-SKSKRSVMVIDWSDL        356
LmxM.27.0430 SIVSADAL--AKSGEDEDG-DDNDTRKRARLEKIVSTLLAKKAK-SKSKRSVMVIDWSDL        356
LINF_270009400 SIVSADAL--AKSGEVDDG-DDNDTRKRARLEKIVSTLLAKKAK-SKSKRSVMVIDWSDL        356
LdBPK.27.2.000440 SIVSADAL--AKSGEVDDG-DDNDTRKRARLEKIVSTLLAKKAK-SKSKRSVMVIDWSDL        356
TcCLB.506925.490 PIRHDGQIHTEDDDDDNED-NNMSKRKKTKLERIIARAIEQQAKRRHGKRSVVVIDWDCD        386
TvY486_1100920 SIVKGGAD------SSEDE-GSNMRRQKSRLESIISRIIEQRRNGPRGKHSVVVIDWDSM        372
 . :: . . : :. :: : . .: *. :*



Tb927.11.1030 SGGLKQVEVHLVDGMGDEEHGDWMPQRVV-----------ASAPQNRKRAPQKRAAERSV        427
LbrM.27.0520 DSDADR------DTAATEEDGEETVVAHKRQRGRSAKSRSV-----A------L-GTEAT        398
LtaP27.0430 DSDADG------DTSTTEKDEEETAMNVKRHRGRPAKSHSI-----A------L-GTEAT        398
LmjF.27.0430 DSDADG------DTAITEEDWEETAVGLKRQRGRPAKSRSI-----A------L-GTEAT        398
LmxM.27.0430 DSDADG------NTSTTDEDGEETAVGLKRQRGRPAKSRSI-----A------L-GTEAT        398
LINF_270009400 DSDADG------DTATTEEDGEETAVALKRRRGRPAKSRSI-----A------L-GTEAT        398
LdBPK.27.2.000440 DSDADG------DTATTEEDGEETAVALKRRRGRPAKSRSI-----A------L-GTEAT        398
TcCLB.506925.490 SCGELLASDADVEE--EETDNDDEEVAMTRAAARSCKEPMSKNPSNAASASATA-A----        439
TvY486_1100920 DSQEVSVLPSRDTERAGAHGGADDTLGVVGAGGAACDVTVPLERRSAPRAPKSV-ARKAV        431
 . .

Tb927.11.1030 PSVSCKK---ESSATPLVAPEVVPVAKRSL-PRPAAPPL---IDELNGGEEPVLLRRSGT        480
LbrM.27.0520 LVSSAKSVQ-KASAKRAA-----LSRKR---HVSAEPELGDSLLFEDESEQPILLPRRQH        449
LtaP27.0430 LVSSAKPTQ-KPSTKRAA-----SSRKR---PASAEPELGDSLLFEDEAEQPILLPRRQN        449
LmjF.27.0430 LVSSAKHVQ-KPSTKRAA-----SSRKR---HVSAEPELGDSLLFEDEAEQPILLPRRQN        449
LmxM.27.0430 LVSSAKHVQ-KPSTKRAA-----SSRKR---HVSAEPELGDSLLFEDEAEQPILLPRRQN        449
LINF_270009400 LVSSAKHVQ-RPSTKRAA-----SSRKR---HASAEPELGDSLLFEDEAEQPILLPRRQN        449
LdBPK.27.2.000440 LVSSAKHVQ-RPSTKRAA-----SSRKR---HASAEPELGDSLLFEDEAEQPILLPRRQN        449
TcCLB.506925.490 SAASTASRQKKQSTTRHVSV-RAPSRKRGANSASVAPDLGA-DSLLLEEDQPVLLRRPAT        497
TvY486_1100920 PHLSAPPERRPASACR-GAS-RCASSAKGVNANARLPSETVEFFPLEDEDQPILLRRPKR        489
 * *: : * ::*:** *



Tb927.11.1030 MQKRPARSVSYISVDTDDMAEANETS--ATVRRPQ---SAPKSTTTTTRRCRKASATAVE        535
LbrM.27.0520 TRPTPTRSISYIDMEGDEDLLRDSSSVERVVRRPPRPTRAP-----AT---------R--        493
LtaP27.0430 KRPAPTRSVSYIDMEGDDDLLRDASSVERVVRRPPRATRAPAIRAPAT---------R--        498
LmjF.27.0430 TRPAPTRSISHIEMGVDDDLLRNVASVERVVRRPPRATRAP-----VT---------R--        493
LmxM.27.0430 TRPAPTRSISYIEMGGDDDLLRDASSVERVVRRPPRATRAP-----AT---------R--        493
LINF_270009400 TRPPPTRSISYIEMGSGDDLLRDAASVERVVRRPPRATRAP-----VT---------R--        493
LdBPK.27.2.000440 TRPPPTRSISYIEMGSGDDLLRDAASVERVVRRPPRATRAP-----VT---------R--        493
TcCLB.506925.490 RRRAPTRSISYMSPEAEDDSVPDHAV--DVSRKPPRRTPAPKAAGRGS---------KAP        546
TvY486_1100920 KEIVATRSISNISSDVDV-TLPQAPV--SVSTSL-RRAPRRKQNSSKQ---------AVP        536
 . :**:* :. : .

Tb927.11.1030 APTCHPPLSNVTHSSPIPP--------DPFLATEEDLESI-------VEEPVTLRRNGHL        580
LbrM.27.0520 --QPRSRLASTLA-------RRGAEDVSSFTGTATCRGRVLQPPALTAGEPTV------A        538
LtaP27.0430 --QRRGRLASSNT-------HEGAEDLSSFTGTTVSRGRAQPPAAAAAGDSTS------A        543
LmjF.27.0430 --QRRIRLASTST-------REGAEDMSSFTDSTAWRGRAPQPPAATTGGATG------V        538
LmxM.27.0430 --QRRGRLASTST-------REGAEVMSSFTGTTALRGRASQPPAAPTGGPTG------V        538
LINF_270009400 --QRRGRLASTST-------REGAEDMSSFTDATAWRGRAPQPPAATTGGPTG------V        538
LdBPK.27.2.000440 --QRRGRLASTST-------REGAEDMSSFTDATAWRGRAPQPPAATTGGPTG------V        538
TcCLB.506925.490 APATGTALSPHRFPSSVPPSVAVAHVSQSFTSARHNSHANVEPLDDPPLPPVNSRRRCRT        606
TvY486_1100920 LANPSDCLASAAY--------------DPFIED----ESQFTPHEVTAAKPS--ARSQRK        576
 *: . *

Tb927.11.1030 NKPTSDWNSFANDDSDVLDNFLVKFVPEN---AGLMLARAN------ICTRKGRRKTRGA        631
LbrM.27.0520 PPRRRYRSAPRADPNDPMAVFFEAAFPSPSKFDEMMMQAGGLPETRRGGGGGGRGQGRHP        598
LtaP27.0430 PPRRRRGSVLRADPNDPMAVFFEAAFPSPSKFDEMMMQAGGLPETRRGG-GGGRGQGRHP        602
LmjF.27.0430 PPCRRRGSAPRADPNDPMAVFFEAAFPSPSKFDEMMMQAGGLPETRRGGGGGGRGQGRHP        598
LmxM.27.0430 PPRRRRGSVQRADPNDPMAVFFEAAFPSPSKFDEMMMQAGGLPETRRGGGGGGRGQGRHP        598
LINF_270009400 PPRRRRGSAPRADPNDPMAVFFEAAFPSPSKFDEMMMQAGGLPETRRGGGGGGRGQGRHP        598
LdBPK.27.2.000440 PPRRRRGSAPRADPNDPMAVFFEAAFPSPSKFDEMMMQAGGLPETRRGGGGGGRGQGRHP        598
TcCLB.506925.490 TSAASRPPATVTSSDDPMAVFFSASFPSPSKFEEMMLAAGGLQETRKVGRG----HPRQP        662
TvY486_1100920 VTSSRQRQVPIMSPNDPMSVFFSADFPSPSKFDEMMLAAGAAPDGRRVQGG----NRRGP        632
 . .* : *: .*. :*: . : *

Tb927.11.1030 ALSLPPSIGGRTR        644
LbrM.27.0520 NLVLPSSIGRRR-        610
LtaP27.0430 NLVLPSSIGRRR-        614
LmjF.27.0430 NLVLPSSIGRRR-        610
LmxM.27.0430 NLVLPSSIGRRR-        610
LINF_270009400 NLVLPSSIGRRR-        610
LdBPK.27.2.000440 NLVLPSSIGRRR-        610
TcCLB.506925.490 ALLLPDSILRRR-        674
TvY486_1100920 TLLLPTSIARNR-        644
 * ** ** .

# **KKT9**

Note: The *L. tarentolae* protein sequence for KKT9 was unusual on TriTrypDB but was not edited to keep consistency.

Tb927.8.1150 ------------------------------------------------------------        0
TcCLB.506401.160 ------------------------------------------------------------        0
LbrM.02.0590 ------------------------------------------------------------        0
LtaP02.0530 MQQHKPSTTTCRSSWWLEHVLAYGRERRTRVFLTCCRGGRGRRFNLEGAAQLRGRSSRSL        60
LmxM.02.0610 ------------------------------------------------------------        0
LdBPK.02.2.000580 ------------------------------------------------------------        0
LINF_020011400 ------------------------------------------------------------        0
LmjF.02.0610 ------------------------------------------------------------        0

Tb927.8.1150 ------------------------------------------------------------        0
TcCLB.506401.160 ------------------------------------------------------------        0
LbrM.02.0590 ------------------------------------------------------------        0
LtaP02.0530 RRRTERRGRAADLRTYVRACSRSNSLLAVRLGFFPLPHVKVTINEELERAHDDSDRYLSS        120
LmxM.02.0610 ------------------------------------------------------------        0
LdBPK.02.2.000580 ------------------------------------------------------------        0
LINF_020011400 ------------------------------------------------------------        0
LmjF.02.0610 ------------------------------------------------------------        0

Tb927.8.1150 ------------------------------------------------------------        0
TcCLB.506401.160 ------------------------------------------------------------        0
LbrM.02.0590 ------------------------------------------------------------        0
LtaP02.0530 DVLRRTLGVPLLSPIPHCRRLRPIPSCFVPTASLVHVVSRSHQVVWGRRCDIATLTCTLA        180
LmxM.02.0610 ------------------------------------------------------------        0
LdBPK.02.2.000580 ------------------------------------------------------------        0
LINF_020011400 ------------------------------------------------------------        0
LmjF.02.0610 ------------------------------------------------------------        0

Tb927.8.1150 ------------------------------------------------------------        0
TcCLB.506401.160 ------------------------------------------------------------        0
LbrM.02.0590 ------------------------------------------------------------        0
LtaP02.0530 GWCVMRVPKHALPAPMYPPLGQQHLWELAVGSWSFXXXXXXXXXXXXXXXXXXXXXXXXX        240
LmxM.02.0610 ------------------------------------------------------------        0
LdBPK.02.2.000580 ------------------------------------------------------------        0
LINF_020011400 ------------------------------------------------------------        0
LmjF.02.0610 ------------------------------------------------------------        0

Tb927.8.1150 ------------------------------------------------------------        0
TcCLB.506401.160 ------------------------------------------------------------        0
LbrM.02.0590 ------------------------------------------------------------        0
LtaP02.0530 XXXXXXXXXXXXXXXXXXXXXXXXXXXXXXXXXXXXXXXXXXXXXXXXXXXXXXXXXXXX        300
LmxM.02.0610 ------------------------------------------------------------        0
LdBPK.02.2.000580 ------------------------------------------------------------        0
LINF_020011400 ------------------------------------------------------------        0
LmjF.02.0610 ------------------------------------------------------------        0

Tb927.8.1150 ------------------------------------------------------------        0
TcCLB.506401.160 ------------------------------------------------------------        0
LbrM.02.0590 ------------------------------------------------------------        0
LtaP02.0530 XXXXXXXXXXXXXXXXXXXXXXXXXXXXXXXXXXXXXXXXQQHTPTPASFFLPYIRRAAP        360
LmxM.02.0610 ------------------------------------------------------------        0
LdBPK.02.2.000580 ------------------------------------------------------------        0
LINF_020011400 ------------------------------------------------------------        0
LmjF.02.0610 ------------------------------------------------------------        0

Tb927.8.1150 ---------------------------------------MSSVLTRSFSSCAGKLEETRE        21
TcCLB.506401.160 ---------------------------------------MSEGLLTQWRDRVVEAETEIS        21
LbrM.02.0590 ---------------------------------------MSEELILQWTKRVSDAEEELS        21
LtaP02.0530 LHPALPVAHTFKGPRVQHPILRPLWWFRSCCDSLSVQAAMSEELIVQWAKRVSDAEEELK        420
LmxM.02.0610 ---------------------------------------MSEELIVQWAKRVSDAEEELH        21
LdBPK.02.2.000580 ---------------------------------------MSEELILQWAKRVSDAEEELH        21
LINF_020011400 ---------------------------------------MSEELILQWAKRVSDAEEELH        21
LmjF.02.0610 ---------------------------------------MSEELILQWAKRVSDAEEELH        21
 **. * .: . . . *

Tb927.8.1150 LIKRDLETARKHRIELRKTIDDDLKRVEKERAELLGIVEEQEAKINRSVREFEEVRSRRE        81
TcCLB.506401.160 VVQEDISALLGRRKEIKQDISRYDRLVQREKQEMLKRLEEVEGQARELEERCQKTVQQKA        81
LbrM.02.0590 RMEEDINAVKQQRKSLKADIHKYEDVVASEKCDLLTSLERVEAQAKDLGAQCEETLTEKD        81
LtaP02.0530 RMEEDINAVKQKRKLLKTDIRKYEDVVASEKCDLLTSLEMVEAQAKDLGAQCEATLTEKD        480
LmxM.02.0610 RMEEDINAVKQQRKLLKTEIRKYEDVVASEKCDLLTSLERVEAQAKDLGAQCEATLTEKD        81
LdBPK.02.2.000580 RMEEDINAVKQKRKLLKTDIRKYEDVVASEKCDLLTSLERVEAQAKDLGAQCEATLTEKD        81
LINF_020011400 RMEEDINAVKQKRKLLKTDIRKYEDVVASEKCDLLTSLERVEAQAKDLGAQCEATLTEKD        81
LmjF.02.0610 RMEEDINAVKQQRKLLKADIRKYEDVVASEKCDLLTSLERVEAQAKDLGAQCEATLTEKD        81
 ::.*:.: :* :: * * *: ::* :* *.: . . : . .:

Tb927.8.1150 WLKKEHDEAVKRYVKMSDTVTFIKEGDKRLQDRTDFEAVLEEENTTWADRERQLIASVST        141
TcCLB.506401.160 RVEEMYVQTLDNYESMHDTVKEIQAEEQRLQEREDVETVLQRESLAWAEEEHGLRTKLNA        141
LbrM.02.0590 CAEREYMATLDAYENLHFLVREIKDAEEHLQSRDDLETRLQRESVIWAEEEHALRRTLHQ        141
LtaP02.0530 RVEAEYMATLDAYENLHFLVREIKDAEEHLQSRDDLEACLQRESVAWAEEEHALRRKLHQ        540
LmxM.02.0610 RVEAEYMATLDAYENLHFLVREIKDAEEHLQSRDDLEVCLQRESITWAEEEHALRRKLHQ        141
LdBPK.02.2.000580 RVEAEYMATLDAYENLHFLVREIKDAEEHLQSRDDLEACLQRESVTWAEEEHALRRKLHQ        141
LINF_020011400 RVEAEYMATLDAYENLHFLVREIKDAEEHLQSRDDLEACLQRESVTWAEEEHALRRKLHQ        141
LmjF.02.0610 RVEAEYMATLDAYENLHFLVREIKDAEEHLQSRDDLEACLQRESVTWAEEEHTLRRKLHE        141
 : : ::. * .: * *: :::**.* *.*. *:.*. **:.*: * .:

Tb927.8.1150 HNTTLKQARKERRKEVETLETELAEMTKKLEQERIARREDLQQESQG---LLRSRRGTPQ        198
TcCLB.506401.160 LQSALKGKRKQRHEEVTALDTELAAAERQLLQVRHERQKDMQAQTPQLQVAKHSRHGTPL        201
LbrM.02.0590 LQQQQTQTRRAQEAELRELEAQLLNVEQRQRDERAGRCG--AAAQAARRVVRSSRQSTPA        199
LtaP02.0530 LQQQQAQARRAQETELRELEAQLSNVEQRQRDERAGRCV--EVAQAARRVVRNSRQPTPA        598
LmxM.02.0610 LQQQQAQARRAQEAELRELEAQLSNVEQRQRDERAGRCV--EVAQAARRVVQSSRQPTPA        199
LdBPK.02.2.000580 LQQQQAQARRAQEAEVRELEAQLSNVEQRQRDERAGRCV--EVAQAARRVVHSSRQPTPA        199
LINF_020011400 LQQQQAQARRAQEAEVRELEAQLSNVEQRQRDERAGRCV--EVAQAARRVVHSSRQPTPA        199
LmjF.02.0610 LQQQQAQARRAQEAEVRELEAQLSNVEQRQRDERAGRCV--EVAQAARRVVHSSRQPTPA        199
 : *: :. *: *:::* :: : * * **: **



Tb927.8.1150 PPSQRPTVAAVHTQAFEPLEANRKEQQFIAINKSSCLPTR--QIRSCFKNNNSTGGNR-F        255
TcCLB.506401.160 ESAQFAVA--------AAADANDKEQQFITANASTSVPTR--QLRSCLKNTSATTDSYSF        251
LbrM.02.0590 SPKS---------------VVPAEEDAFVRANTGGAMPTRSAPLRSCLKMPVSGHTSGN-        243
LtaP02.0530 SPKP---------------VTSAEEDAFVRVNAGGAMPIRSAPLKSCLKTPISGGTGGN-        642
LmxM.02.0610 SPKP---------------VTSAEEDAFVSANAGGAMPIRSAPLKSCLKTPVSGSTSGS-        243
LdBPK.02.2.000580 SPKP---------------VTSAEEDAFVSANAGGALPIRSAPLKSCLKTPVSGSTGSN-        243
LINF_020011400 SPKP---------------VTSAEEDAFVSANAGGALPIRSAPLKSCLKTPVSGSTGSN-        243
LmjF.02.0610 SPKP---------------VTSAEEDAFVSANAGGAMPIRSAPLKSCLKTPVNGSTSGN-        243
 . :*: *: * . .:* * ::**:* .

Tb927.8.1150 SNE-LETGRNTVANRRRSTNGSGSTGQGVSQHFHSAPVSRATSQSLVSGDSENIVKI---        311
TcCLB.506401.160 SDTKTQPGNGSI--NKNSTGSSSVCHTVPATNFKSAPVSRAVSQALVAGDDTV------L        303
LbrM.02.0590 ----------------------AAATAPRPPTAASAPVSRCASQSLVSDSAASGVGRQGI        281
LtaP02.0530 ----------------------AATTTPHPTTAASAPVSRCASQSLVSDSAASGVQRQGV        680
LmxM.02.0610 ----------------------TATTAPRPTTAASAPVSRCASQSLVSDAAVSGVQRQGV        281
LdBPK.02.2.000580 ----------------------AATTAPRPTTAASAPVSRCASQSLVSDAAASGVQRQGV        281
LINF_020011400 ----------------------AATTAPRPTTAASAPVSRCASQSLVSDAAASGVQRQGV        281
LmjF.02.0610 ----------------------AAPTAPRHTTAASAPVSRCASQSLVSDAAASGVQRQGV        281
 ******..**:**:.



Tb927.8.1150 --------RGAASQRAFSYADTKRVAGRKHEL--LRDSTNI-        342
TcCLB.506401.160 GGVDS---THVTPHRAYSYASGKKAGSRKREIILLGDATNR-        341
LbrM.02.0590 DGGDRPSLQVLPEMRAYSYGAPAKPGARKREF--LGDTTNNQ        321
LtaP02.0530 DGVDQSSLRLLQEMRAYSYGAPCKAGARKREF--LGDATNHP        720
LmxM.02.0610 DGGDQPSLRLLQEMRAYSYGAPCKAGARKREF--LGDATNSQ        321
LdBPK.02.2.000580 DGGDQPSLRLLQEMRAYSYGAPCKAGARKREF--LGDATNNQ        321
LINF_020011400 DGGDQPSLRLLQEMRAYSYGAPCKAGARKREF--LGDATNNQ        321
LmjF.02.0610 DGGDQPSLRLLQEMRAYSYGAPCKAGARKREF--LGDATNNQ        321
 **:**. : ..**:*: * *:**


# **KKT17**

Tb927.3.2330 MKSRQPTKNCINMHDIVAQPLDAVVGAINATLRRPEELDDAEVRSIFEETSFILLRAEEA        60
LbrM.25.1800 -MPRQPTKNRINMHDIVEQPLSAIPDAVDAVLRRPEPLDELEVQSIFFESSYILLRAEEA        59
LtaP25.2340 -MPRQPTKNRINMHDIVEQPLSAIPDAVDAVLRRPEPLDELEVQSIFFESSYILLRAEEA        59
LmjF.25.2220 -MPRQPTKNRINMHDIVEQPLSAIPDAVDAVLRRPEPLDELEVQSIFFESSYILLRAEEA        59
LmxM.25.2220 -MPRQPTKNRINMHDIVEQPLTAIPDAVDAVLRRPEPLDELEVQSIFFESSYILLRAEEA        59
LdBPK.25.2.002320 -MPRQPTKNRINMHDIVEQPLSVIPDAVDAVLRRPEPLDELEVQSIFFESSYILLRAEEA        59
LINF_250029300 -MPRQPTKNRINMHDIVEQPLSAIPDAVDAVLRRPEPLDELEVQSIFFESSYILLRAEEA        59
TcCLB.508479.240 MKSRQPTKNRINMHDIIATPLGALPGAVDAILRRPENLDDVEVQSIFKEASFILLRAEEA        60
TvY486_0301690 --MPRPARNSINMHDIIAQPLGAITGAVNAILHRQVDLDDAEVESIFDEAPFLLLRAKEA        58
 :*::* ******: ** .: .*::* *:* **: **.*** *: ::****:**

Tb927.3.2330 LMASEEAAFRALFTLLNKGFAHSAESFGKGVEVFSSQVMLLEVLESIFHRAVKLQVFPLQ        120
LbrM.25.1800 LSAKDGNAFCALFKIFSEGFAHSETSFEEGIRTVVQNDSLLQTMHSIFKVVVEQEALPLH        119
LtaP25.2340 LSAKDGNAFCALFKIFSEGFAHSEASFEEGIRTMVQNESLLQTMYTIFKVVVEQEALPLH        119
LmjF.25.2220 LSAKDGNAFCALFKIFSEGFAHSESSFEKGIRTMVQNESLLQTMQAIFKVVVEQEALPLH        119
LmxM.25.2220 LSAKDGNAFCALFKIFSEGFAHSEASFEEGIRTMVENESLLQTMHAIFKVVVEQEALPLH        119
LdBPK.25.2.002320 LSAKDGNAFCALFKIFSEGFAHSEASFEEGIRTMVQNESLLQTMHAIFKVVVEQEALPLH        119
LINF_250029300 LSAKDGNAFCALFKIFSEGFAHSEASFEEGIRTMVQNESLLQTMHAIFKVVVEQEALPLH        119
TcCLB.508479.240 LASREADAFCVLFKLFSEGLAHSAASFGKGIATFGENGALLQTLRDIFRVAVEQRAFPVQ        120
TvY486_0301690 MAACDADAFCALFKLLLEGFSHSEIFLIKGVETFSTQHTLLQTLSEIFTEAVTHRRFPLQ        118
 : : : ** .**.:: :*::** : :*: .. : **:.: ** .* . :*::

Tb927.3.2330 GEAVAALLYTVGNLCDGDGTKDSCGRLFIGGLANILVHMYSQDVEEAHQHFVTQRAAVGA        180
LbrM.25.1800 SAGTVELIQVLSSLCDGAALKDKCGAVFMRGFAKVLQRAYS-NAEEARRHFHVQRGTATA        178
LtaP25.2340 SAGTVELIQVLSSLCDGAALKDKCGAVFMRGFAKILQRVYS-NAEEARRHFHVQRGTATA        178
LmjF.25.2220 SAGTVELIQVLSSLCDGAALKDKCGAVFMRGFAKVLQRVYS-NTEEARRHFHVQRGTTTA        178
LmxM.25.2220 SAGTVELIQVLSSLCDGAALKDRCGALFMRGFAKVLQRVYS-NTEEARRHFHVQRGTATA        178
LdBPK.25.2.002320 SAGTVELIHVLSSLFDGAALKEKCGAVFMRGFAKVLQRVYS-NAEEARRHFHLQRGTATA        178
LINF_250029300 SAGTVELIHVLSSLFDGAALKEKCGAVFMRGFAKVLQRVYS-NAEEARRHFHLQRGTATA        178
TcCLB.508479.240 CPAVIELVQVVGSLCDGSCIKDTCGALFMEGLVYMLDDMYRRSIEEAHAGFLAQRAAATA        180
TvY486_0301690 SEAVGELLRVIGSLCDGS-IRNVCAALFMEGLASVLSDMYRENLEEARACFQTQRAAAAS        177
 .. *: .:..* ** :: *. :*: *:. :* * . ***: * **.:. :

Tb927.3.2330 MIKLMKRSPRNRKLITSWDFLANCCALSVDPFFQLQCIELLYRVSRSNKSIFKQMKSRLS        240
LbrM.25.1800 LINLVKGSKQNKQRLASWKFVADCCAASVDVFFQLQCVELLFRVSRQNKDVFSHLGGSLP        238
LtaP25.2340 LINLVKGSKQNKQRLASWKFVADCCAASVDVFFQLQCAELLFRVSRQNKDVFSHLGGSLP        238
LmjF.25.2220 LINLVKGSKQNKQRLASWKFVADCCAASVDVFFQLQCVELLFRVSRQNKDVFSHLGGSLP        238
LmxM.25.2220 LINLVKGSKQNKQRLASWKFVADCCAASVDVFFQLQCVELLFRVSRQNKDVFSHLGGSLP        238
LdBPK.25.2.002320 LINLVKGSKQNKQRLASWKFVADCCAASVDVFFQLQCVELLFRVSRQNKDVFSHLGGSLP        238
LINF_250029300 LINLVKGSKQNKQRLASWKFVADCCAASVDVFFQLQCVELLFRVSRQNKDVFSHLGGSLP        238
TcCLB.508479.240 LINLVKGSKQNKQRITSWDFLADCCALSVDVFFQLQCIELLFRLSRHNKSLLPHICSRLR        240
TvY486_0301690 LIQLVKGSKQNKQRILSWEFLAKCCALSVDFFFQLQCVELFFRVSWHNQALLTNLGTHLH        237
 :*:*:* * :*:: : **.*:*.*** *** ****** **::*:* *: :: :: *

Tb927.3.2330 TEVIEKLASLTNSATLISDMMGVLAVINRDRESLLVFPLGACEVAGVDLNGSMTCYFAPH        300
LbrM.25.1800 LATIEELRALPNDGTLLSRMTLLIEHLNEGRAQVLRYPLKEVVAAETTLTSSTNAYFTPN        298
LtaP25.2340 PATIEELRALPNDGTLLSRMTLLIEHLNEGRAQVLRYPLKEVVAAETTLTSSTNAYFTPN        298
LmjF.25.2220 PATIEELRALPNDGTLLSRMTLLIEHLNEGRAQVLRYPLKEVVAAETTLTSSTNAYFTPN        298
LmxM.25.2220 PATIEELRALPNDGTLLSRMTLLIEHLNEGRAQVLRYPLKEVVAAETTLTSSTNAYFTPN        298
LdBPK.25.2.002320 PATIEELRALPNDGTLLSRMTLLIEHLNEGRAQVLRYPLKEVVAAETTLTSSTNAYFTPN        298
LINF_250029300 PATIEELRALPNDGTLLSRMTLLIEHLNEGRAQVLRYPLKEVVAAETTLTSSTNAYFTPN        298
TcCLB.508479.240 PQVLEDIRQLPNDSTLLTKMVELLHNINAGREDILLFPLSRADVAYTTIMENTFSYFTMN        300
TvY486_0301690 PTVIERIRQLPNDSTLITKMAEVVASINNGREDVLIFPLTSTDVASAKVTGDTVSCFTEK        297
 .:* : * *..**:: * :: :* .* .:* :** .* . : . . *: :

Tb927.3.2330 YFVLIS-SDAVTESTIPYSSIRSAKFVEPSGVAIRVHEFPASLVAGLSRKTPKGDTLTLR        359
LbrM.25.1800 YVIVMVTAANADNITIPYRIIRSITLGRDGRVIVRLEEFPVKLELLLSR-TAGMDTVTFY        357
LtaP25.2340 YVIVMVTAANADNITIPYRIIRSITLGRDGRVIVRLEEFPVKLELLLSH-TTGMDTVTFY        357
LmjF.25.2220 YVIVMVTAANADNITIPYRIIRSITLGRDGRVIVRLEEFPVKLELLLSH-TAGMDTVIFY        357
LmxM.25.2220 YVIVMVTAANADNITIPYRIIRSITLGRDGRVIVRLEEFPVKLELLLSH-TTGMDTVTFY        357
LdBPK.25.2.002320 YVIVMVTAANADNITIPYRIIRSITLGRDGRVIVRLEEFPVKLELLLSH-TTGMDTVTFY        357
LINF_250029300 YVIVMVTAANADNITIPYRIIRSITLGRDGRVIVRLEEFPVKLELLLSH-TTGMDTVTFY        357
TcCLB.508479.240 YFVVLVTSSNADNVTIPYNSIRSVTLGKDGRVVFRLDEFPTKLEALLNR-AAGEDTIVLF        359
TvY486_0301690 YFVVIVMSDNADNITIPYDTIRSVMLRKNGRVTFRLNEFPSKLELLLTR-APGEDTVSLV        356
 *.::: : . : **** *** : . . * .*:.*** .* *.: : **: :

Tb927.3.2330 VSREQLQRFKSSSIHSWITAAMKTREGAHQT--PVGAPGAGNNERWSSGVTPVASDH---        414
LbrM.25.1800 MAQEDLATFKQSPVRQWIVEALQARKEEQHHHLPPQAPRSPDSTAKPAGKVEWATHKPLS        417
LtaP25.2340 MAQEELAAFKQSPVRRWIVEALQVRKEEQRHHRPPQAPRSPDSTAKPVEMTERAAHESQP        417
LmjF.25.2220 MAQEELATFKQSPVRQWIVEALQVRKEEQRHHRPPQAPRSPDSMAKPAEKTEPAAHESLS        417
LmxM.25.2220 MAQEELATFKQSTVRQWIVEALQVRKEEQRHHRPPQAPRSPDSMVKPAEKAEPVAHESLS        417
LdBPK.25.2.002320 MAQEDLATFKQSPVRQWIVEALQVRKEEQHHHRPPQAPRSPDSMAKPAEKTEPAAHESLS        417
LINF_250029300 MAQEDLATFKQSPVRQWIVEALQVRKEEQHHHRPPQATRSPDSMAKPAEKTEPAAHESLS        417
TcCLB.508479.240 MTVDQLALFKESRIRSWIVSALEAKKERKRNGARSGAEAEAVTTS--------ASHN---        408
TvY486_0301690 MDPERLDAFKASSIRSWIVATLDSRRGQRQKSGDENINSDRKSGS----TALLFSRE---        409
 : : * ** * :: **. ::. :. :: . : .



Tb927.3.2330 ------------------PQDGSGRKRYREESVQSAGSGKPGTTSD-------L------        443
LbrM.25.1800 STATAASGAAAAAPCEASGDIDTARKKARLESA--TTA-AVVPYTNQPPIGALLHGVDAL        474
LtaP25.2340 STAPAISDAAAASSHEVSSDMDTTRKKARLEGAT-TCS-ATVPYTNQPSIAALLHVVDAL        475
LmjF.25.2220 STTPA------ASSHEVSDDMVAARKKARLEGGAASAA-AAVPYTNQPPIAAFLHVVDAL        470
LmxM.25.2220 STTPA------ASLHETSDDTVTARKKARLEGAAAS-A-AAVPCTNHPPIAALLHVVDAL        469
LdBPK.25.2.002320 STTPA------ASSHEASDDMATTRKKARLEGSAAP-A-AAVPSTNQPPIAAFLHVVDAL        469
LINF_250029300 STTPA------ASSHEASDDMVTTRKKARLEGSAAP-A-AAVSSTNQPPIAAFLHVVDAL        469
TcCLB.508479.240 --API----------------GGGTKRQRSDSLQLTGGDS-------QVLAVF-------        436
TvY486_0301690 --RQD----------------TSSEKKHRAESVSLVEGNKTTDFGSRQVAPAF-------        444
 *: * :. .



Tb927.3.2330 -KNVGSSEGPTSGIMKHSAEAVKLMSEVFPQKVTSIMSESINKIQEAVDGARVTTDGYRS        502
LbrM.25.1800 VEQATTPNEAQAM-LLQLRRLMEARKEVQRGKSLDDLSDAMQRIQSRVDEARRTAKENRA        533
LtaP25.2340 VEQASTPNEAQAM-LLQLRRLMEARKEMQRGKSMDDLSDAMQHIQSRVDETRHTAKENRM        534
LmjF.25.2220 VEQAATPNEAQAM-LLQLRRLMEARKEMQRGKGMDDLSDAMRHIQNRVDETRRTAEESRT        529
LmxM.25.2220 VEQAATPNEAQAM-LLQLRRLMEARKEMQRGKSMDNLSDAMRHIQNRVDETRRTAKENRT        528
LdBPK.25.2.002320 VEQAATPNEAQAM-LLQLRRLMEVRKEMQRGKSMDDLSDAMRHIQNRVDETRRTAKESRT        528
LINF_250029300 VEQAATPNEAQAM-LLQLRRLMEVRKELQRGKSMDDLSDAMRHIQNRVDETRRTAKESRT        528
TcCLB.508479.240 QKIMKASDENASMVLEKMKQLINSKHECRQEEVAAILKASMDDIQRMVDEGHVANDGVRD        496
TvY486_0301690 NEETGTDKLHES----AAAGVL--GNDGNINKRTAALSATMVNIQHLVDGGRCIINDHRE        498
 : : . : : : : :. :: ** ** : . *

Tb927.3.2330 HVKSAVEGAIQLVEDSLMASHSKAAAAVEKLNHELQERKATDTAFHQRIACIEIAAQQAL        562
LbrM.25.1800 AWHQAMLEELERVEALVVGAQDKAAAGVEQLNDHLKKVKASNQAINERIACMDVELQRTL        593
LtaP25.2340 AWHQAMLDELVRVENLVVGAQDKAAEGVEQLNDHLKKVKTSNQAINERIACMDIELQQTL        594
LmjF.25.2220 AWHQTMWEELERVESVVVGAQDKAAAGVEQLNDHLKKVKTSNQAINERIACMDIELQQTL        589
LmxM.25.2220 AWHQAMLEELERVENVVVGAQDKAAAGVEQLNDHLKKVKASNQAINERIACMDIELQQTL        588
LdBPK.25.2.002320 AWHQAMLEELERVENVVVGAQDKAAAGVEQLNDHLKKVKTSNQAINERIACMDIELQQTL        588
LINF_250029300 AWHQAMLEELERVENGVVGAQDKAAAGVEQLNDHLKKVKTSNQAINERIACMDIELQQTL        588
TcCLB.508479.240 TLRGGVEESIQSIEQRLLDSQTKAAGVVEQLNVALQELKSSNTAIHEQIACIEIILQQSL        556
TvY486_0301690 AFRHGAEKNIQNIESCLQASQEKATLMVERLNDALQKLKDGNAAIHDQLACIEIQLQVTL        558
 : : :* : :: **: **:** *:: * : *:::::**::: * :*

Tb927.3.2330 EESRESEVRSLDSIKKEFEQHAAKYAAALDDELIRLSNPVSTLFNIFNSEGNQSIC        618
LbrM.25.1800 EELREEERRWTSEIHTKCMREAERLEFEVDQQLLNRSRPMALLSDYMRH-------        642
LtaP25.2340 EELREQELRWTNEIHTKCMREAERLEFEVDQHLVSRSRPMALLSDYMRQ-------        643
LmjF.25.2220 EELREEELRWTNEIYTKCMREAERLEFEVDQHLVNRSRPMTVLSDYMRQ-------        638
LmxM.25.2220 EELREEELRWTNEIHTKCMREAERLEFEVDQHLVNRSRPMALLSDYMRQ-------        637
LdBPK.25.2.002320 EELREEELRWTNEIHTKSMREAERLEFEVDQHLVNRSRPMALLSDYMRQ-------        637
LINF_250029300 EELREEELRWTNEIHTKSMREAERLEFEVDQHLVNRSRPMALLSDYMRQ-------        637
TcCLB.508479.240 EVSREEEASMCNSLKADGEESIERIEALLDRQLIGQSNPMSVISEFLRTSTEGSVL        612
TvY486_0301690 EESREKEAKRYDLLRAEGEERIEQLEHMLDSQLMRRSSPLKAISRFLQTDVK----        610
 * **.* . : . . : :* .*: * *: : :.

# **KKT26**

LtaP17.0770 MDTCSAAEPSPSPRLCDAVKAEPNTEDERGQKPQELSRRQDVVSSATTLDFLALWRPSAA        60
LmxM.17.0650 MDTASAAEPSPPPRLRDAVKAEPSTEDERGQQ--QLSRHQDVASSASALDFLALWRPSAA        58
LmjF.17.0650 MDTASAAAPSPPPRLRDAVKAEPSTEDERGQQLQQLSRHQDVASSASALEFLALWRPSAV        60
LdBPK.17.2.000790 MDTASAAEPSPPPRLRDAVKAEPSTENERGQQLQQLSRHQDVASSASALDFLALWRPSAV        60
LINF_170013900 MDTASAAEPSPPPRLRDAVKAEPSTENERGQQLQQLSRHQDVASSASALDFLALWRPSAV        60
Tb927.7.6480 ----------------------------------------------------MNQSPFDH        8
TvY486_0706420 --------------------------------------------------------MSER        4
TcCLB.507979.40 --------------------------------------------------------MDRQ        4

LtaP17.0770 PSSRHADALTNANHREEMKGVPSSSSVPPP-SAQAAPTHAQDPRLGTLSIGSVVFAPYRM        119
LmxM.17.0650 SSSRHAAALTNAKQAEEVKRAQLSSSSPPS-SALDAPTRVCDPRLGTLSIGSVVFAPYRV        117
LmjF.17.0650 SSSRHAAALTSAKQVEEVKRAQLSSSPPPS-SELAAPTHVYDPRLGTLSIGSVVFAPYRV        119
LdBPK.17.2.000790 SSSRHAAALTNAKQVE-VQRAKSSSSPPPS-SELTTPTHVYDPRLGTLSIGSVVFAPYRV        118
LINF_170013900 SSSRHAAALTNAKQVE-VQRAKSSSSPPPS-SELTTPTHVYDPRLGTLSIGSVVFAPYRV        118
Tb927.7.6480 PRSCHGKEV----------GSASDTAA------RSACTRCRGAATHGFAVGETVYAPKR-        51
TvY486_0706420 PRRCVGGAS----------GARQEHQPLFRAGQPPKREQPAQDLRMGIAVGDTVFAPKR-        53
TcCLB.507979.40 TR----GWT----------RERRGRQPSP---GSAATRLMARGLGDGFAVGSVVFAPRR-        46
 :::*..*:** *

LtaP17.0770 CSERPVRGGLSACVTSGTVSSSPVAAAAPHQLVRPTRSPYHLHYALAEVTGIQAMAGTVT        179
LmxM.17.0650 CTERPVRGGLSPCVTGGIVSGSPVTAAAPHQLMPPPPPPYRLHYALAEVTGIQVMAGTVT        177
LmjF.17.0650 CSERPVRGGLSACVTGDTVSGSPVTAAAPHQLMPPPRPPYRLHYALAEVTGIQVMAGTVT        179
LdBPK.17.2.000790 CSERPARGGLSACVAGGTVSGSPVTAAAPHQLMPPPRSPYRLHYALAEVTGIQVMAGTVT        178
LINF_170013900 CSERPARGGLSACVAGGTVSGSPVTAAAPHQLMPPPRSPYRLHYALAEVTGIQVMAGTVT        178
Tb927.7.6480 ------------------------------------SEGKPLRYALAEVTGIQATARTVT        75
TvY486_0706420 ------------------------------------CSNGAHRYIPSEVTGIQASARTAS        77
TcCLB.507979.40 ------------------------------------RRAGALHYALAEVTGIQACAGTAT        70
 :* :******. * *.:

LtaP17.0770 VSFLFTDPGIDDEAVSMYECVPCPPACLSRWLLEEDSRAPQRCSLLERLLHITPAKGPLP        239
LmxM.17.0650 VSFLFTDPGIDDEAVSMYECVPCPPACLSRWLLEENSPAPQRCSLLERVLHVTPPEGPLP        237
LmjF.17.0650 VSFLFTDPGIDDEAVSMYECVPCPPACLSRWLLEENSPSPQHCSLLERVLHVTPPEGPLP        239
LdBPK.17.2.000790 VSFLFTDPGIDDEAVSMYECVPCPPACLSRWLLEESSPAPQRCSLLERVLHVTPPEGPLP        238
LINF_170013900 VSFLFTDPGIDDEAVSMYECVPCPPACLSRWLLEESSPAPQRCSLLERVLHVTPPEGPLP        238
Tb927.7.6480 VSFLGTEPGVDDEAVPFYTVVPSPGEVIPTVDNTNG--GAYFSPFRR--VGKSGAGAG--        129
TvY486_0706420 VSFLLDEPDAEDTIVPFYSLIPCPRSVLAEG-------GI-GGPLPSMPAGENSMQAG--        127
TcCLB.507979.40 VAFVNAEPGVDDGAVPLYVLVPCPWPDFARG-------GV-RCSFAERALRAAARHEP--        120
 *:*: :*. :* * :* :*.* : . :

LtaP17.0770 VNDVYRALHAVSPKLARPPGLSSSLAQRQRQRERCVARLRQLYCLSDDDDEQPQTTTPRN        299
LmxM.17.0650 VNDVYRALHAPSPKLTSAPGLSPLLVQRQRQRERCVGRLRQLYCLSDDDDEHPDTTMARN        297
LmjF.17.0650 VNDIYRALQAASPKLASAPGLSPLLVQRQRQRERCVARLRQLYCLSDDDDEQPHTTMAHN        299
LdBPK.17.2.000790 VNDVYRALHAASPKFASAPGLSPLLVQRQRQRERCVARLRRLYCLSDDDDEQPHTTMARN        298
LINF_170013900 VNDVYRALHAASPKFASAPGLSPLLVQRQRQRERCVARLRRLYCLSDDDDEQPHTTMARN        298
Tb927.7.6480 ------------STLNPPRGKGGKTRVDPAKNSNDNELSGVEVCRRNA--EAP-------        168
TvY486_0706420 ------------A--HGETGSGGKWA-------------HFVREDRAA--EDS-------        151
TcCLB.507979.40 ------------T--LSDTARGQQ-----------------LSCDSDC--GGE-------        140
 . .



LtaP17.0770 SRGVVQGVSEEDMAAKPVTLDPTATEVT-PSSAAWRVLSTAAAGEAASTVSRVGTTTLAG        358
LmxM.17.0650 SRGVAQGASEEDMAAKPVALEPTAATAT-PSSAAWRFLSTGTTDKAALTVGSGGTTTPAA        356
LmjF.17.0650 SRGVAQGASGEDMAAKPVALEPTAATAA-PSSVAWRFLSTATTEKVALTVGSAGTTTLAA        358
LdBPK.17.2.000790 SRGVAQSASGEDMAAKPVALEPTAATAT-PSSAAWRFLSTATTEKAALTVGSAGTTTLAA        357
LINF_170013900 SRGVAQSASGEDMAAKPVALEPTAATAT-PSSAAWRFLSTATTEKAALTVGSAGTTTLAA        357
Tb927.7.6480 ----ERGIP---------TLC--------QLAEAFP--SDV---------------QQRN        190
TvY486_0706420 ----LGAIPN--------TVKTTALESAQPFTEKFN--VKLE--------------RDEE        183
TcCLB.507979.40 ----VGDGPPPSDTAAPPTPPQTPSSSPPPSS-------GEA--------------EDEG        175
 : :

LtaP17.0770 SVATIFARYVAPTSIHVFFSGIFQEVNAYMEHCCNRHCCHSLPDPTTEPLPSPRGGLRRG        418
LmxM.17.0650 SAATMFARYVAPTSVHVFFSGIFQEVNAYMEHHCSRHRRHALTDPTADPLASPSRRLRRG        416
LmjF.17.0650 SAATMFARYVAPTSVHAFFSGIFQEVNACMEHHCSRHRRHSLTDPTTEPLSSLGRRLRRG        418
LdBPK.17.2.000790 SAATIFARYVAPTSVHVFFSSIFQEVNVYMEHHCSRHRRHSLTDPTAEPLSSPSRRLRRG        417
LINF_170013900 SAATMFARYVAPTSVHVFFSSIFQEVNVYMEHHCSRHRRHSLTDPTAEPLSSPSRRLRRG        417
Tb927.7.6480 GWRVLCVPHHHDESFNAFSARAFGAACLTMNGCL--------------------------        224
TvY486_0706420 DLYSVQFSPAQFENADAYYFHAFKTVGRWILGHF--------------------------        217
TcCLB.507979.40 EQGTIRVTHAPPESVGAFFARAFRAVCRAILRRP--------------------------        209
 : . .: * . :

LtaP17.0770 VAAINAAPPPPLVLVIFDSYAMLRRFHVFMTVRGHIVQLLDGNHMGRTLPSP--QTSHAF        476
LmxM.17.0650 VAAANAASPPPLILVIFDSYATLRRFHVFMTVRGHVVQLLDGTHAGRALPSP--QTSHAF        474
LmjF.17.0650 VAAANAASPPPLVLVIFDSYATLRRFHVFMSVRGHVVQLLDGTHVGRALPSP--RTSHAF        476
LdBPK.17.2.000790 VAAVNAASPPPLVLVIFDSYATLRRFHVFMSVRGHVVQLLDGAHVDRALPSP--QTSHAF        475
LINF_170013900 VAAVNAASPPPLVLVIFDSYATLRRFHVFMSVRGHVVQLLDGAHVDRALPSP--QTSHAF        475
Tb927.7.6480 -KSAPRTTGGWVVLSVFTTYDSLHHFDVYMTLRGYRVCLADGTVHPTGFRGGPAVSSIQY        283
TvY486_0706420 -PP-ALVAQWRVVLCVFEAYSTLHRFQVYMSLRGHQLCLVDGA-----------------        258
TcCLB.507979.40 -ADWQPAVQEHVVLCVFDTYDALRRFNVYMAFRGHRLYLIDGVCARAA----------PA        258
 . ::* :* :* *::*.*:*:.**: : * **

LtaP17.0770 GAQCKIWLCMLRDDTGEEGGEEEAKRKYGS-SIARAS-GEESVEAVQAMAVMEAQLRRLL        534
LmxM.17.0650 GAQCKIWLCMLRDDTGEEVVEEEVTKEDGS-SITRASAGEESAEVVQAVALMETQLRCLL        533
LmjF.17.0650 GAQCKIWLCMLRDDTGEESVEEEAKKEDGF-SITRASAGEESVEVAQAVALMEAQLRCLL        535
LdBPK.17.2.000790 GAQCKIWLCMLRDDTGEESVEEEAKKEDGF-SIARASAGEESAEVAQAVALMEAQLRCLL        534
LINF_170013900 GAQCKIWLCMLRDDTGEESVEEEAKKEDGF-SIARASAGEESAEVAQAVALMEAQLRCLL        534
Tb927.7.6480 DERETLWLCILADDIANEGSASALVGEWLPCGVQRQHPNAGKC--GD--NLI--------        331
TvY486_0706420 ------------------------------------------------------------        258
TcCLB.507979.40 AAGDVSWLCLLAEDVRDERAALGLLGGWLPPGRWREGSGALTV-----------------        301

LtaP17.0770 EATPSVDALVSFREHVVE-----NRQSATLHHHTTAEAVLQRLMPYLGARR---LECVLQ        586
LmxM.17.0650 AAAPPVDALVSFKEHVTQQQ-----------QQVTAEAVLQRLIPHLGEDR---LECVLQ        579
LmjF.17.0650 AAAPPVDALVSFKEHVTQQQVTKSRRSATLHQHVTAEAVLQRLMPHLGADR---LECVLQ        592
LdBPK.17.2.000790 EAAPPVDALVSFREHVTQQQVTKSRQSATLHQHVTAEAVLQRLMPHLGAYR---LECVLQ        591
LINF_170013900 AAAPPVDALVSFREHVTQQQVTKSRQSATLHQHVTAEAVLQRLMPNLGAYR---LECVLQ        591
Tb927.7.6480 -----KVGVVQFVEEWKKE----KVESEPWMESDDVERAIRKLLAAVGADAHTLIKCHLP        382
TvY486_0706420 ------------------------------------------------------------        258
TcCLB.507979.40 ------ASAVWFTEDCGDTDC-DERNAEGLSRDGGVDAAMGRLLGAVGG-AGALITCRLS        353

LtaP17.0770 DVPTPPPRPTPVGNNAAASSS---GTAQQQSSTAAATTVVKVSGAVFGDNKILVPCSPEQ        643
LmxM.17.0650 DVPAPPPRPTPVGNNAAASPS---GTTQPPPSTAPATTVVKVSGAVFGDNKILVPCSPEQ        636
LmjF.17.0650 DVPAPPPRPTPVGNNAAASSS---GTTQPPPSTAPATTVVKVSGAVFGDNKILVPCSPEQ        649
LdBPK.17.2.000790 DVPAPPPRPTPVGNNAAASSS---GTTQPPPSTAPATTVVKVSGAVFGDNKILVPCSPEQ        648
LINF_170013900 DVPAPPPRPTPVGNNAAASSS---GTTQPSPSTAPATTVVKVSGAVFGDNKILVPCSPEQ        648
Tb927.7.6480 DHVGE---DELVISSLMSDGSTVIGSKWWKQSR-SSSTVRQRT---RPTFELCLPATSWQ        435
TvY486_0706420 ------------------------------------------------------------        258
TcCLB.507979.40 RGTKD---DELMATSLAATCRRRAP---PRQKL-GAAGPFRRR---DPSMELRVPASTWQ        403

LtaP17.0770 LTLLSTVLASTPVAVCEKAGDGGAPKGDGETAEHRKGSATKKTCRKRQRVMDTADSSSAT        703
LmxM.17.0650 LTLLSTVLASTPVAVCRETGDGGAAEGGGETAGQRKCSPTKKANRKRPRVMGTADSSSEA        696
LmjF.17.0650 LTLLSTVLTSTPVAVSRKTGDGGAAEGGGETAGQRKCSPTKKANRKRPRVTETADSSSAT        709
LdBPK.17.2.000790 LTLLSTVLASTPVAVSQKTGDGGAAEGGGETAGQRKCSPTKKANRKRPRVMETADSSSAT        708
LINF_170013900 LTLLSTVLASTPVAVSQKTGDGGAAEGGGETAGQRKCSPTKKANRKRPRVMETADSSSAT        708
Tb927.7.6480 IDLLRALE----------------PRGGLIKQEDTDFPPP------------SASNAADN        467
TvY486_0706420 ------------------------------------------------------------        258
TcCLB.507979.40 VDLFRLLV----------------ARGGGEEPL------P------------S-------        422

LtaP17.0770 AAARAQALTPALLERISLGSFTDAAAATLFDVFADATAVVKKEPGPYSADLHAAPGDTHC        763
LmxM.17.0650 AAARAQALTPALLERIALGSFTDAAAATLFDVFADATAVVMKAPDPSSADLPGTAGDAHR        756
LmjF.17.0650 AVARAQALTPALLERIALGSFTDAAAATLFDVFADATAVVMRAPGPSSEDLPATAGDAHR        769
LdBPK.17.2.000790 AVARAQALTPALLERIALGSFTDAAAATLFDVFADATAVVMKSPDPSSADLPATAGDAHR        768
LINF_170013900 AVARAQALTPALLERIALGSFTDAAAATLFDVFADATAVVMKSPDPSSADLPATAGDAHR        768
Tb927.7.6480 EYARGRVSSPTPLQAIANGDVFNTALSRAVA-----------------------------        498
TvY486_0706420 ------------------------------------------------------------        258
TcCLB.507979.40 ----AAPLLPQLLQSIAFGNVFDVSLSTALA-----------------------------        449

LtaP17.0770 GSSDGRGAAEVLSSIEDLYEKWCVDSSRFG-EAFPVFAVVYALVADVFASASRFRLSRLR        822
LmxM.17.0650 GSSDGRGAATVLSTIEDLYEKWCVDPSRFG-EAFPVFAAVYALVADVFASVSRFRPGRPR        815
LmjF.17.0650 GSSDGSGAAEVLSSIEDLYEKWCVDPSRFG-EAFPVFAAVYALVADVFASVSRFRADRPR        828
LdBPK.17.2.000790 GSSDGRGAAEVLSSIEDLYEKWCVDPSRFG-EAFPVFAAVYALVADVFASVSRFRPGRPR        827
LINF_170013900 GSSDGRGAAEVLSSIEDLYEKWCVDPSRFG-EAFPVFAAVYALVADVFASVSRFRPGRPR        827
Tb927.7.6480 ---CGE-GA---GLLRDLQRRVVCNAGFLGEEVSPLFCAIHAVLQDVLPQPESGTAVQ--        549
TvY486_0706420 ------------------------------------------------------------        258
TcCLB.507979.40 ---SLPGGL---RQLDGIRERVVRSGAFFGDVAFPALAAARAVLADVLAAPLAERASR--        501

LtaP17.0770 GAGGRGAGIGDDQQRRLSTEPLGSIPSLERVADMKASAVTQGHLPAPLPPSGLPGGRLGA        882
LmxM.17.0650 STSGWGTGIGDDGPRRLSTGPLESAPSLEQAAEMKASTVTQRRLPTPSPPYELPRGRLGA        875
LmjF.17.0650 SASEWDAGIGDDGPRRLSSEPLESAPSLEQATEMKASAVTQRRLPTPSPPNELPRGRLGA        888
LdBPK.17.2.000790 SASEWDASIGDEGPRRLSSGPLESAPSLEQAAE-KASAVTQRRLPTQSPPNELPRGRLGA        886
LINF_170013900 SASEWDASIGDEGPRRLSSGPLESAPSLEQAAEMKASAVTQRRLPTQSPPNELPRGRLGA        887
Tb927.7.6480 ------------------------------------------------------------        549
TvY486_0706420 ------------------------------------------------------------        258
TcCLB.507979.40 ------------------------------------------------------------        501

LtaP17.0770 LFGALPRIALVLPRGNPTHHPNLSTQQYLRTLRAFFSPWAVHEVASPVASV---AVSCAA        939
LmxM.17.0650 LLRALPRIALVLPRGNPTQHPNLGTQQYLRTLRAFFSPWAVHEVSSPAASV---AVSCAA        932
LmjF.17.0650 LLGALPRIALVLPRGNPTQHPNLSTQQYLRTLRAFFSPWAVHEVTSPAASV---AISCAA        945
LdBPK.17.2.000790 LIGALPRIAIVLPRGNPTQHPNLGTQQYLRTLRAFFSPWAVHEVTSPAASV---AVGCAA        943
LINF_170013900 LIGALPRIALVLPRGNPTQHPNLGTQQYLRTLRAFFSPWAVHEVTSPAASV---AVSCAA        944
Tb927.7.6480 ----WKRVVICLPGVSN---RASASVAYRHAIQRFLYPWRLHFLATDEWQRTS-DSATME        601
TvY486_0706420 ------------------------------------------------------------        258
TcCLB.507979.40 ----QRRVVLVLPGAGG---APDDAAAYLHAVQRFLHPWHLFTLAAEKGQRQPCVEALLD        554

LtaP17.0770 PVLWHQRGGLLLLFCDEATTQLGALQDEADVVIACGKAAAAWVAANSATKARSGDSCKVA        999
LmxM.17.0650 PVLWHQRGGLLLLFCDEATTQLGALQDEADVVIACGKAAAAWVAANSATKAGSGDGCKVA        992
LmjF.17.0650 PVLWHQRGGLLLLFCDEATTQLGALQDEADVVIACGKAAAAWVAANSATKASSGDSCNVA        1005
LdBPK.17.2.000790 PVLWHQRGGLLLLFCDEATTQLGALQDEADVVIACGKAAAAWVAANSATKASSGDSCKVA        1003
LINF_170013900 PVLWHQRGGLLLLFCDEATTQLGALQDEADVVIACGKAAAAWVAANSATKASSGDSCKVA        1004
Tb927.7.6480 SLSWLQMGGVMLLSMEEPLCQLSSLKEEADIVIECGKCSATPR-TP-----------RDG        649
TvY486_0706420 ------------------------------------------------------------        258
TcCLB.507979.40 GRAWLENGGVLLLNPEEPLGRLSLLQEEADVVITCGKASAALVAGC-----------CRG        603

LtaP17.0770 VPDQLLFAVISEAEVVAPTDQLTHLWLPITLASQTCIREGHAPVDVSTSADGLDRCSEWP        1059
LmxM.17.0650 AAGPLLFAVISEAEVVAPTDHLTHLWLPITLASPTCMGEGHAPADVSTSADGLERCSEWP        1052
LmjF.17.0650 AAGPLLFAVISEAEVVAPTDQLTHLWLPITLASPTCMGGGHAPADVSTSADGLERCSEWP        1065
LdBPK.17.2.000790 AAGPLLFAVISEAEVVAPTDQLTHLWLPITLASPTCMGGGDAPADVSTSADGLERCSEWP        1063
LINF_170013900 AAGPLLFAVISEAEVVAPTDQLTHLWLPITLASPTCMGGGDAPADVSTSADGLERCSEWP        1064
Tb927.7.6480 VT---HIVLFSEVEIPGGPTPLLSSWRPANKAIDRALEQ---------------------        685
TvY486_0706420 ------------------------------------------------------------        258
TcCLB.507979.40 AP---HIALHSEVELAEGPAHRWTLWRGAAGEADATDAE---------------------        639

LtaP17.0770 AMSKEETWRTELEEMWRLLVATVSEDGSVNVSARPSSPLLRQDHQQHGETLRSAGAAARR        1119
LmxM.17.0650 AMSKEETSRTELEEMWRLLVATVSADGSVDVSARPSSPLLRQDQQQHGQTLRSTGAAARR        1112
LmjF.17.0650 AMSKEETSRTELEEMWKLLVAMVSADGSVNVSARPSSPLLRQDQQQHGQTLRSTGAAARR        1125
LdBPK.17.2.000790 AMSKEETSRTELEEMWRLLVATVSADGSVDVPARPSSPLLRQDQQQHGQTLRSTGVAARR        1123
LINF_170013900 AMSKEETSRTELEEMWRLLVATVSADGSVDVPARPSSPLLRQDQQQHGQTLRSTGVAARR        1124
Tb927.7.6480 -------------AVVRRTMSHTQPSVGTHIFMKAAALIEELDGKENHQTE-------R-        724
TvY486_0706420 ------------------------------------------------------------        258
TcCLB.507979.40 ------------RAVLAAAVAA-GGEGAAPVLATAAALLHRLGRPIEGKSEAA----EV-        681

LtaP17.0770 LHQAEVLPRTLRQAVVLWRHLQTAEA--TMCGRQCQC---CVSSSDVDSEGPWGTLQAVV        1174
LmxM.17.0650 LHQVEVLPRTLRQAVVLWRHLQAADP--NLCGRQRQS---CASPSEFDNEGPWGTLQAVV        1167
LmjF.17.0650 LHQVEVLPRTLRQAVVLWRHLQAAEP--AMCGWQRQR---CASPSELDSEGPWGTLQAVV        1180
LdBPK.17.2.000790 LHQVEVLPRTLRQAVVLWRHLQTAEA--TVCGGQRQS---RASPSELDNEGPWGTLQAVV        1178
LINF_170013900 LHQVEVLPRTLRQAVVLWRHLQTAEA--TVCGGQRQS---RASPSELDNEGPWGTLQAVV        1179
Tb927.7.6480 ---RMLSRKRQTVEDEAWVHLRKFAVHLLVCSCHFQETR---------------------        760
TvY486_0706420 ------------------------------------------------------------        258
TcCLB.507979.40 ---VGGARRWSGQAAGDWAHLRRLAVAIALCGCRPPVPSFCEAAR---------------        723

LtaP17.0770 KEMALSCATATPVRMTDVTLIRSSDSA-        1201
LmxM.17.0650 KEMALSCVTATPVRMTDVTLLRGNDDSA        1195
LmjF.17.0650 KEMALGCVTATPVRMTDVTLLRGSDDSA        1208
LdBPK.17.2.000790 KEMALSCVTATPVRMTDVTLLRGSDDSA        1206
LINF_170013900 KEMALSCVTATPVRMTDVTLLRGSDDSA        1207
Tb927.7.6480 ----------------------------        760
TvY486_0706420 ----------------------------        258
TcCLB.507979.40 ----------------------------        723
