# Supplementary material for: CLK1/CLK2-driven signalling at the Leishmania kinetochore is captured by spatially referenced proximity phosphoproteomics
Source: Commun Biol. 2022 Nov 28;5:1305. doi: 10.1038/s42003-022-04280-1 (PMC9701682; doi:10.1038/s42003-022-04280-1)
Supplement: Supplementary file 11 — reporting summary [file 42003_2022_4280_MOESM11_ESM.pdf]

nature portfolio

Corresponding author(s): Jeremy C Mottram

Last updated by author(s): Nov 14, 2022

Reporting Summary

Nature Portfolio wishes to improve the reproducibility of the work that we publish. This form provides structure for consistency and transparency in reporting. For further information on Nature Portfolio policies, see our Editorial Policies and the Editorial Policy Checklist.

Statistics

For all statistical analyses, confirm that the following items are present in the figure legend, table legend, main text, or Methods section.

n/a	Confirmed	
☐	☒	The exact sample size (*n*) for each experimental group/condition, given as a discrete number and unit of measurement
☐	☒	A statement on whether measurements were taken from distinct samples or whether the same sample was measured repeatedly
☐	☒	The statistical test(s) used AND whether they are one- or two-sided<br>*Only common tests should be described solely by name; describe more complex techniques in the Methods section.*
☐	☒	A description of all covariates tested
☐	☒	A description of any assumptions or corrections, such as tests of normality and adjustment for multiple comparisons
☐	☒	A full description of the statistical parameters including central tendency (e.g. means) or other basic estimates (e.g. regression coefficient) AND variation (e.g. standard deviation) or associated estimates of uncertainty (e.g. confidence intervals)
☐	☒	For null hypothesis testing, the test statistic (e.g. *F*, *t*, *r*) with confidence intervals, effect sizes, degrees of freedom and *P* value noted<br>*Give P values as exact values whenever suitable.*
☒	☐	For Bayesian analysis, information on the choice of priors and Markov chain Monte Carlo settings
☒	☐	For hierarchical and complex designs, identification of the appropriate level for tests and full reporting of outcomes
☒	☐	Estimates of effect sizes (e.g. Cohen's *d*, Pearson's *r*), indicating how they were calculated

Our web collection on statistics for biologists contains articles on many of the points above.

Software and code

Policy information about availability of computer code

Data collection	Positive ESI-MS and MS2 spectra were acquired using Xcalibur software (version 4.0, Thermo).
Data analysis	Mass spectrometry data were analysed with Progenesis QI (Version 2.2., Waters), Mascot Daemon (version 2.6.1, Matrix Science) was used to submit searches to a locally-running copy of the Mascot program (Matrix Science Ltd., version 2.7.0.1). Data were analysed with R version 4.0.1 (2020-06-06)

For manuscripts utilizing custom algorithms or software that are central to the research but not yet described in published literature, software must be made available to editors and reviewers. We strongly encourage code deposition in a community repository (e.g. GitHub). See the Nature Portfolio guidelines for submitting code & software for further information.

Data

Policy information about availability of data

All manuscripts must include a data availability statement. This statement should provide the following information, where applicable:

- Accession codes, unique identifiers, or web links for publicly available datasets
- A description of any restrictions on data availability
- For clinical datasets or third party data, please ensure that the statement adheres to our policy

Mass spectrometry data sets and proteomic identifications are available to download from MassIVE (MSV000087750), [doi:10.25345/C5G543] andProteomeXchange (PXD027080).

Field-specific reporting

Please select the one below that is the best fit for your research. If you are not sure, read the appropriate sections before making your selection.

☒ Life sciences ☐ Behavioural & social sciences ☐ Ecological, evolutionary & environmental sciences

For a reference copy of the document with all sections, see nature.com/documents/nr-reporting-summary-flat.pdf

Life sciences study design

All studies must disclose on these points even when the disclosure is negative.

Sample size	No sample size calculations were performed
Data exclusions	For label free protein quantifications, peptide ions with pearson correlation scores of <=0.4 were excluded from quantification so that only those ions with consistent intensity profiles across samples were retained (outlined in Methods section)
Replication	Essentiality of KKT24 and KKT26 was confirmed with a repeat experiment, mass spectrometry experiments were not repeated.
Randomization	Not relevant as samples allocated to groups based on harvest timepoint in cell cycle
Blinding	Not relevant as samples allocated to groups based on harvest timepoint in cell cycle

Reporting for specific materials, systems and methods

We require information from authors about some types of materials, experimental systems and methods used in many studies. Here, indicate whether each material, system or method listed is relevant to your study. If you are not sure if a list item applies to your research, read the appropriate section before selecting a response.

Materials & experimental systems

n/a	Involved in the study	
☐	☒	Antibodies
☐	☒	Eukaryotic cell lines
☒	☐	Palaeontology and archaeology
☒	☐	Animals and other organisms
☒	☐	Human research participants
☒	☐	Clinical data
☒	☐	Dual use research of concern

Methods

n/a	Involved in the study	
☒	☐	ChIP-seq
☒	☐	Flow cytometry
☒	☐	MRI-based neuroimaging

Antibodies

Antibodies used	anti-myc 4A6 (millipore), anti-HA 2-2.2.14 (invitrogen), anti-mCherry 16D7 (invitrogen), anti-EF1a CBP-KK1 (millipore)
Validation	Validated by manufacturers

Eukaryotic cell lines

Policy information about cell lines

Cell line source(s)	Leishmania mexicana (MNYC/BZ/62/M379)
Authentication	None
Mycoplasma contamination	Not tested
Commonly misidentified lines (See ICLAC register)	None
